# Anomalous diffusion on the servosphere: A potential tool for detecting inherent organismal movement patterns

**DOI:** 10.1371/journal.pone.0177480

**Published:** 2017-06-01

**Authors:** Naohisa Nagaya, Nobuaki Mizumoto, Masato S. Abe, Shigeto Dobata, Ryota Sato, Ryusuke Fujisawa

**Affiliations:** 1 Department of Intelligent Systems, Faculty of Computer Science and Engineering, Kyoto Sangyo University, Motoyama, Kamigamo, Kita-ku, Kyoto-City, Japan; 2 Laboratory of Insect Ecology, Graduate School of Agriculture, Kyoto University, Kitashirakawa-oiwakecho, Sakyo-ku, Kyoto, Japan; 3 National Institute of Informatics, 2-1-2 Hitotsubashi, Chiyoda-ku, Tokyo, Japan; 4 ERATO Kawarabayashi Large Graph Project, Japan Science and Technology Agency, Hitotsubashi 2-1-2, Chiyoda-ku, Tokyo, Japan; 5 Department of Mechanical Engineering, Hachinohe Institute of Technology, Ohbiraki, Myo, Hachinohe, Aomori, Japan; Nanjing University, CHINA

## Abstract

Tracking animal movements such as walking is an essential task for understanding how and why animals move in an environment and respond to external stimuli. Different methods that implemented image analysis and a data logger such as GPS have been used in laboratory experiments and in field studies, respectively. Recently, animal movement patterns without stimuli have attracted an increasing attention in search for common innate characteristics underlying all of their movements. However, it is difficult to track the movements in a vast and homogeneous environment without stimuli because of space constraints in laboratories or environmental heterogeneity in the field, hindering our understanding of inherent movement patterns. Here, we applied an omnidirectional treadmill mechanism, or a servosphere, as a tool for tracking two-dimensional movements of small animals that can provide both a homogenous environment and a virtual infinite space for walking. To validate the use of our tracking system for assessment of the free-walking behavior, we compared walking patterns of individual pillbugs (*Armadillidium vulgare*) on the servosphere with that in two types of experimental flat arenas. Our results revealed that the walking patterns on the servosphere showed similar diffusive characteristics to those observed in the large arena simulating an open space, and we demonstrated that our mechanism provides more robust measurements of diffusive properties compared to a small arena with enclosure. Moreover, we showed that anomalous diffusion properties, including Lévy walk, can be detected from the free-walking behavior on our tracking system. Thus, our novel tracking system is useful to measure inherent movement patterns, which will contribute to the studies of movement ecology, ethology, and behavioral sciences.

## Introduction

The most outstanding feature of animals is that they move around their environments to search for food, mates, and habitats or to avoid predators. The movements can be formed by internal and external factors [1]. The internal factors include spontaneous neuronal activities [2], physiological states and memories. There may still be other factors, and it might be difficult to identify and tell them apart. The external factors are environmental cues such as visual, chemical, tactile, or auditory stimuli. The goal of behavioral ecology and neuroscience is to identify these factors forming animal movements and to understand why and how the animals move. In the past two decades, studies on trajectories of animal movements have reported that animals exhibit scale-free movements (i.e., Lévy walks) or anomalous diffusion, which leads to efficient searches [3]. The Lévy walks are described as a combination of many short movement steps and rare long-distance displacements. The length of these displacements follows power-law probability density function *P*(*x*_i_) ~ *x*_i_^-μ^, where *x*_i_ is the move length (displacement of consistent direction) and μ represents the power-law exponent, which is 1 < μ ≤ 3. However, there is a debate whether the pattern is induced by the internal factors or the external factors [4,5].

Researchers have developed a variety of methods to track animal movements empirically. Image-based tracking is widely employed to analyze free movements of relatively small animals in the laboratory [6,7]. In field studies, small data loggers with GPS are attached to animals to track their movements in the air, on the land, and in the sea [8]. In those methods, however, space constraints caused by the use of wall-bounded arenas (e.g., petri dishes) in the laboratory and a high degree of environmental heterogeneity in the field are inevitable, rendering it difficult to distinguish spontaneous behaviors from the entangling environmental factors or to track the individual movement over a long period of time [9,10]. An ideal experimental setup would be to give animals a vast and homogeneous environment without any stimuli, which is usually unrealistic. Therefore, we need experimental setups that can detect internal factors during free movement.

A possible solution is the use of the locomotion compensator with a sphere on which a test animal is settled. In this system, called omnidirectional treadmill mechanism (OTM), movements of the animal are continuously recorded and compensated in such a way that the animal always locates on the top of the sphere and experiences a virtual unbounded two-dimensional field. The OTM, also known as Kramer-treadmill [11,12], has been used to study animal orientation toward olfactory [13–16], aerial [17], or visual stimuli [18–21]. However, despite its potential, how animals move on the OTM without external factors remains unexplored.

In this study, first, we developed a system named ANTAM by updating the Kramer-treadmill systems. ANTAM was originally developed as a vehicle for insects, mainly ants, placed on the sphere as a cockpit, and here it was used to characterize two-dimensional movements of small animals on OTM with external stimuli restricted as much as possible. Then, we tested the utilities of the ANTAM by comparing the free-walking behavior of individual pillbugs (*Armadillidium vulgare*) between different tracking conditions—our OTM and flat arenas. We also analyzed the trajectories on the servosphere to examine whether individual pillbugs perform scale-free movements, including Lévy walks, without any external cues.

## Materials and methods

### Mechanism of a locomotion compensator used by the servosphere system

The design requirements of ANTAM were as follows: 1) the locomotion compensation system should be omnidirectional; 2) it should track test animals without placing any markers on them or tethering them; 3) the servomotor should have a structure in which mechanical vibration hardly propagates to the sphere; and 4) it should be controlled with a software that works in commercially available laptop PCs.

**Fig 1:**
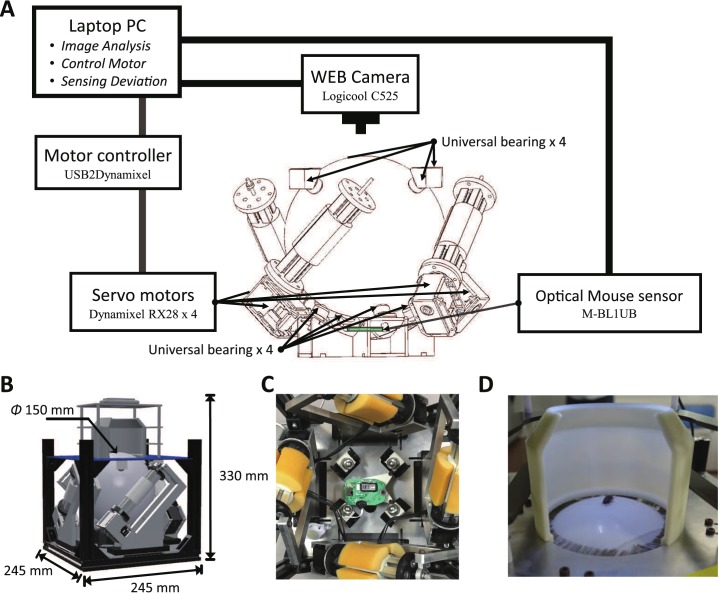
System diagram of the ANTAM (animal tracking device-accompanied mobile suit). (A) System chart with wireframe model of the mechanical part of ANTAM. (B)3D model of the ANTAM (C) The optical mouse sensor is fixed under the sphere, and four universal bearings are arranged around and support the sphere. (D) A photograph of a pillbug walking freely on the ANTAM during moving compensation.

Fig 1A shows a system chart of the ANTAM. The detection of movement of test animals was based on processing of images captured with a web camera (Logicool C525; Logicool Co., Tokyo, Japan), and the locomotion compensation was implemented in the servosphere system by which a spherical ball was circumvolved by servomotors with arbitrary parameters. The measurement of the movement trajectory was performed by an optical mouse sensor (M-BL1UB; Elecom Co., Ltd., Osaka, Japan) fixed under the sphere (Fig 1C).

The test animal on top of the sphere was surrounded by a cylindrical shell created in a 3D printer from ABS resin (Fig 1D). Together with LED arrays placed around the web camera, these devices were intended to control the optical and visual conditions of the test animal. The LED auxiliary lighting can adjust the illuminance within the range from the minimum of 507 lux to the maximum of 1200 lux. The lack of detectable directionality in the trajectories obtained in our experiments suggests that the light distribution from the LEDs was homogenous enough not to give any visual clues (S1 Dataset). The camera was fixed to a camera stand made of four duracon pillars and an acrylic plate cut with a laser cutter; the height from the top plate to the camera was 102 mm. Four servo motors (Dynamixel RX-28; Robotis Co., Ltd., Lake Forest, CA, USA) were arranged around the ball acting as an actuator to rotate the sphere; the fixed part of the motor and the whole frame were made of aluminum. A static electricity removal brush (STAC404; TGK, Tokyo, Japan) was attached to remove static electricity by bringing the bristle tip into contact with the sphere.

### Servosphere mechanism

The sphere was a white hollow acrylic ball with a diameter of 150 mm driven by four motors to rotate the sphere as described above. We used the Partially Sliding Roller (PSR) mechanism [22] as a power transmission technique from the motors to the sphere. The use of the PSR mechanism allows direct power transmission from the motor to the sphere while controlling the smooth omni-directional motion via an omni-wheel or the like, which otherwise requires the increase in size of the whole system. In addition, the use of a 13-mm-thick urethane sponge as the contact surface between the PSR mechanism and the sphere relaxed the friction and reduced the vibration from the motor as compared to the alternative use of rubber. The driving force was transmitted from the side of the sphere, and the vertical direction was fixed with eight ball bearings in total, four at the top and four at the bottom (Fig 1A). From the above configuration, the total size of the apparatus with the camera was 245 × 245 × 330 mm (Fig 1B), and the weight was approximately 4.34 kg.

For camera image analysis, the raw image data were binarized to a grayscale format using OpenCV 2.4.9 library, and converted to black-and-white so that test animals became black and backgrounds became white. The centroid of the black area was calculated together with the deviation from the center of the image. The speed output of the motor was determined by the proportional–integral–derivative (PID) control with respect to the obtained amount of deviation, which determines the motion of the sphere. Suitable PID controller parameters needed to be determined for different species of animals in preliminary experiments. In case of the species tested here (*Armadillim vulgare*) a PD controller proved sufficient. We needed to determine the value of PID from the results of preliminary experiments. The image size used for real-time image detection was 320 × 240 pixel, so that a maximum of 30 frames per second (FPS) could be achieved.

### Experimental setup

In this study, we obtained the walking trajectories of individual pillbugs to assess the validity of the servosphere as a tracking tool. We collected 29 individuals that were longer than 10 mm on the grounds of Kyoto University (35.030794°N, 135.785492°E) on August 2, 2016. The animals were kept individually in plastic containers filled with paper towel (Kimtowel; Crecia, Tokyo, Japan) and maintained at a constant temperature (25 ± 1°C) under 16 h light (7:00 to 23:00) and 8 h dark cycle for 5 days until the following experiments.

**Fig 2:**
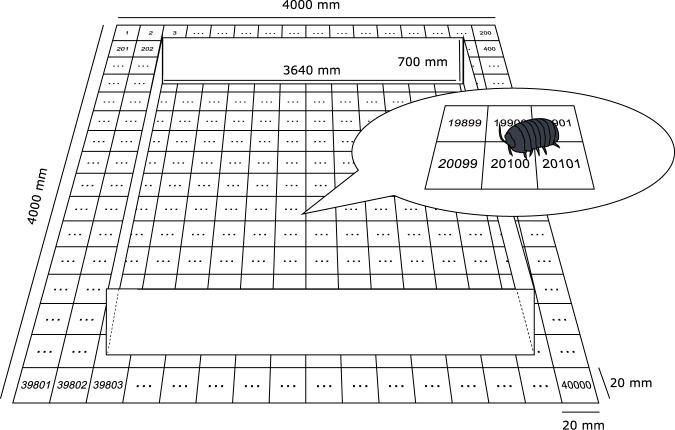
Diagram of the large arena. At the beginning of each experiment, a single pillbug was placed in the center of the arena, which is the lattice point surrounded by lattices numbered 19900, 19901, 20100, and 20101.

The free-walking behavior of the individuals on the servosphere was tracked for 1800 sec. We placed the ANTAM in an environmental chamber at constant temperature (25 ± 1°C) and under constant darkness. The ANTAM was connected to a laptop PC (VAIO, VPCZ23AJ; SONY Corp., Japan, with installed Windows 7 operating system), and the gain values of proportional, integral and derivative controls were set to 5, 0, and 2, respectively. Thus, functionally we used a PD controller for locomotion compensation in the present experiments. The threshold value for binarization was set to 70. To compare the walking trajectories obtained from tracking the animal on the servosphere with those obtained from plane arenas, we prepared two types of experimental arenas, a small arena and a large arena. The small arena had a wall-enclosed circular surface, which has often been used for tracking the free-walking behavior of small animals under laboratory conditions (for example [10]). We prepared a white polystyrene board with white, round plastic wall (φ = 300 mm; height = 50 mm) as an experimental enclosure. We placed a single pillbug in the center and tracked its movement continuously for 30 min with a digital video camera (NEX-VG30, SONY Corp, Japan) at a resolution of 1920 × 1080 pixels and a frequency of 25 FPS. The large arena was designed to measure the walking patterns of animals under homogenous and borderless conditions. We measured the walking patterns of individuals until they left the effective area of the arena. The effective area of the arena was 3.6 m by 3.6 m, set inside a larger area to reduce any visual clues (Fig 2). We placed a single pillbug in the center of the paper and tracked the free-walking behavior for 1800 sec or until it left the effective area. As the large arena was too large to record the walking behavior of individuals with video cameras, we printed a grid (spacing of 20 mm) with grid lines numbered from 1 to 40,000 and recorded the locations of animals using binoculars every 10 seconds (Fig 2). Although this grid lines and letters were visible to the animals, individuals did not trace the grid lines during the experiments. To ensure accurate recordings, the locations of individuals were checked by two observers and recorded by video camera (NEX-VG10, SONY Corp., Japan) whenever possible.

We measured the walking trajectories of 29 individuals across the three tracking conditions during the light cycle (servosphere: 11:00–20:00; small arena: 12:00–18:00; large arena: 10:00–18:00) to account for the any effects of circadian rhythms. The experimental order was prepared using the following sequences with 5–6 replications per sequence: E_1_E_2_E_3_, E_1_E_3_E_2_, E_2_E_1_E_3_, E_2_E_3_E_1_, E_3_E_1_E_2_, and E_3_E_2_E_1_, where E_1_ is the sequence of the servosphere, E_2_ is the sequence of the small arena, and E_3_ is the sequence of the large arena. The surface of the small arena and the servosphere were cleaned with 70% ethanol before each trial.

The study animal is not endangered or protected. All experiments were conducted under the regulation of Kyoto University Ethics Committee (http://www.kyoto-u.ac.jp/en/research/ethic/arcku).

### Data analysis

All the analyses were conducted using R v3.1.3 software [23] with ‘exactRankTests,’ ‘survival,’ and ‘MASS’ packages. The sampling rate of the locations of animals depended on webcams and laptops and varied around 30 FPS. To compare movement patterns on the servosphere and the small arena, we reduced the frame frequency of both data to 5 FPS, whereas to compare the movement pattern between the servosphere and the large arena, the rate frequency of the data was reduced to 0.1 FPS and location data were converted into corresponding lattices of the large arena. To compare basic characteristics of the movement patterns between the tracking conditions, we computed the instantaneous speed as the distance covered by the pillbug from one frame to the next and the direction of movement as the angle between the corresponding displacement and the horizontal axis. Turning angles were then identified as the magnitude of changes in the direction of motion from one frame to the next. We used Wilcoxon signed-rank test to determine whether the representative values of the speed and the turning angle were different between the tracking conditions.

To compare the diffusive properties of individual movements between the servosphere and the large arena, we analyzed the time until leaving the area of 3.6 × 3.6 m by generating Kaplan-Meier survival curves and using both log-rank tests and Wilcoxon tests to check for a difference between tracking conditions. The results of both tests were similar, so we report only the *P* values given by the log-rank tests. We also analyzed the distance traveled by individuals until leaving the area using Wilcoxon signed-rank test.

We evaluated the mean squared displacement (MSD) to compare the diffusive properties of individual movements between the servosphere and the small arena. It was difficult to evaluate MSD of the data in the large arena because of the low sampling rate. The MSD is defined as the mean of squared distance that an organism travels from its starting location to another point during a given time. As a macrospecific property of random walks, the MSD is proportional to the time to the power of α, MSD ~ *t*^α^, where α characterizes the behavior of diffusive processes. In normal diffusion, as is the case of Brownian motion, the MSD increases linearly with time (α = 1). Anomalous diffusion can involve subdiffusive (α < 1) or superdiffusive (α > 1) processes. In this study, we performed linear regression tests on the log-log transformations to evaluate α.

To test for the presence of intrinsic Lévy-like search behavior on the servosphere, we used the methods described by Humphries et al. [24]. The trajectories obtained on the servosphere with 1 FPS were separated into sequences of moves (i.e., quasi-linear track segments at which the individuals did not change direction) to obtain the step-length distribution. We used a one-dimensional segmentation method that identifies direction reversals in single dimensions [25]. The move length distributions were determined separately for each individual, because apparent Lévy walks might arise from pooling the movements of animals that actually perform Brownian walks with typical move lengths of different sizes [26]. Maximum likelihood estimation was used to fit model parameters (exponential or truncated Pareto; TP) to step-length distributions, with model selection by *w*AIC, where a weight of 1 provides strongest support and 0 no support for Lévy walks.

## Results

**Fig 3:**
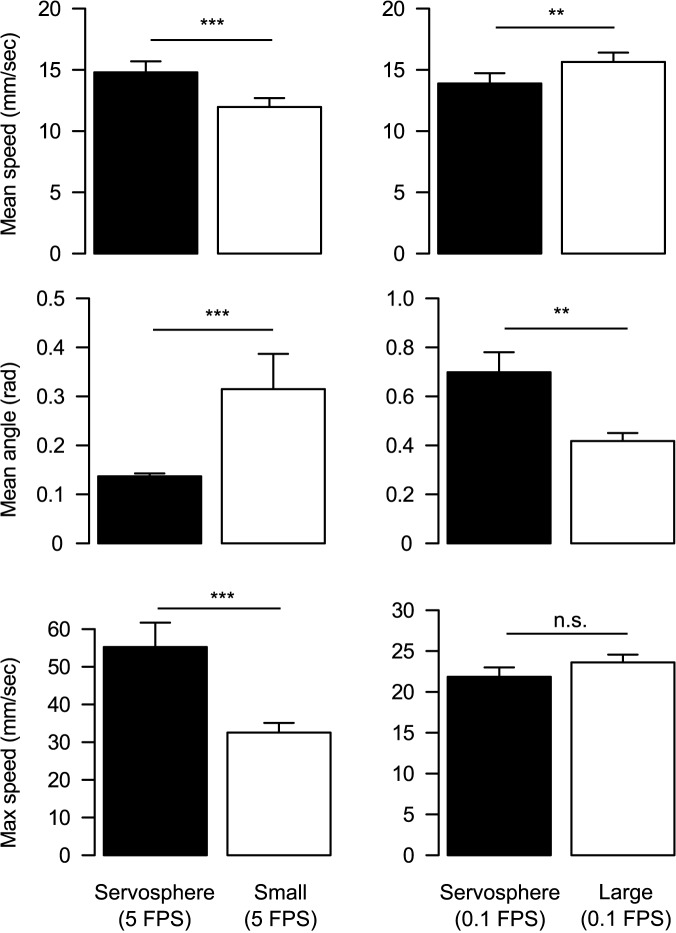
The comparison of movement characteristics between servosphere and arenas. Bars represents standard errors. The asterisks indicate the presence of significant differences (**: *P* < 0.01, ***: *P* < 0.001, Wilcoxon signed-rank test). Note that the values for servosphere (filled bars) are different between left and right columns because of the difference in FPS.

We could not obtain the whole set of trajectories from the three tracking conditions in four individuals because of molting, death, and accidental leg breaking during successive measurements. Thus, we obtained 28 trajectories on the servosphere, 28 in the small arena, and 26 in the large arena (S1 Datasets). The mean speed (FPS: average ± s.d.) on the servosphere (5 FPS: 14.8 ± 4.8 mm/sec; 0.1 FPS: 13.9 ± 4.5 mm/sec) was statistically significantly higher than that in the small arena (5 FPS: 12.0 ± 3.8 mm/sec) and lower than that in the large arena (0.1 FPS: 15.6 ± 3.9 mm/sec) (Wilcoxon signed-rank test; servosphere vs. small arena: *V* = 336, *P* = 0.0002; servosphere vs. large arena: *V* = 71, *P* = 0.0067; Fig 3, S1 Fig). The mean turning angle (FPS: average ± s.d.) was also significantly different between tracking conditions, where the angle on the servosphere (5 FPS: 0.137 ± 0.032 rad; 0.1 FPS: 0.698 ± 0.433 rad) was smaller than that in the small arena (5 FPS: 0.315 ± 0.380 rad) and larger than that in the large arena (0.1 FPS: 0.418 ± 0.167 rad) (Wilcoxon signed-rank test; servosphere vs. small arena: *V* = 12, *P* < 0.0001; servosphere vs. large arena: *V* = 289, *P* = 0.0029; Fig 3, S1 Fig). The maximum speed (FPS: average ± s.d.) was significantly higher on the servosphere (5 FPS: 55.2 ± 34.3 mm/sec; 0.1 FPS: 23.6 ± 4.9 mm/sec) than that in the small arena (5 FPS: 32.5 ± 13.6 mm/sec), but there was no significant difference between the maximum speed on the servosphere and that in the large arena (0.1 FPS: 21.8 ± 6.1 mm/sec) (Wilcoxon signed-rank test; servosphere vs. small arena: *V* = 321, *P* = 0.0009; servosphere vs. large arena: *V* = 98, *P* = 0.1434; Fig 3, S1 Fig).

**Fig 4:**
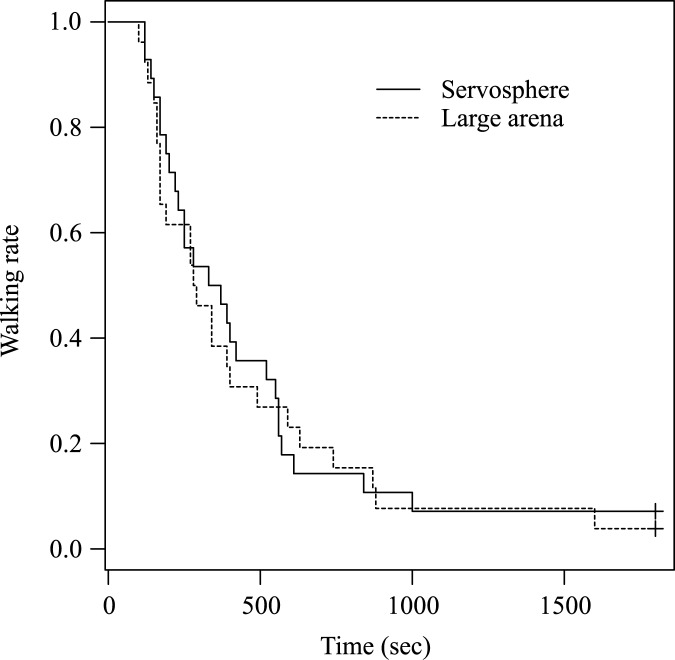
Diffusion process of pillbugs on the servosphere and in the large arena. The time until leaving the area of 3.6 × 3.6 m is compared between individuals mounted on the servosphere and those in the Large arena. The + symbol represents that the individuals were still walking within the arena after 1800 sec (the data were then treated as “right-censored” in the survival analysis).

On the large arena, 24 out of 26 analyzed individuals (92%) left the area of 3.6 × 3.6 m within 1800 sec. There was no significant difference (average ± s.d.) in the time until leaving the area between individuals on the servosphere (4.9 ± 3.1 m) and those in the large arena (5.8 ± 4.2 m) (log-rank test; *χ*^2^_1_ = 0.1; *P* = 0.782, Fig 4). We also did not find any significant differences in the traveled distances within the areas (Wilcoxon signed-rank test: *V* = 164, *P* = 0.7835).

**Fig 5:**
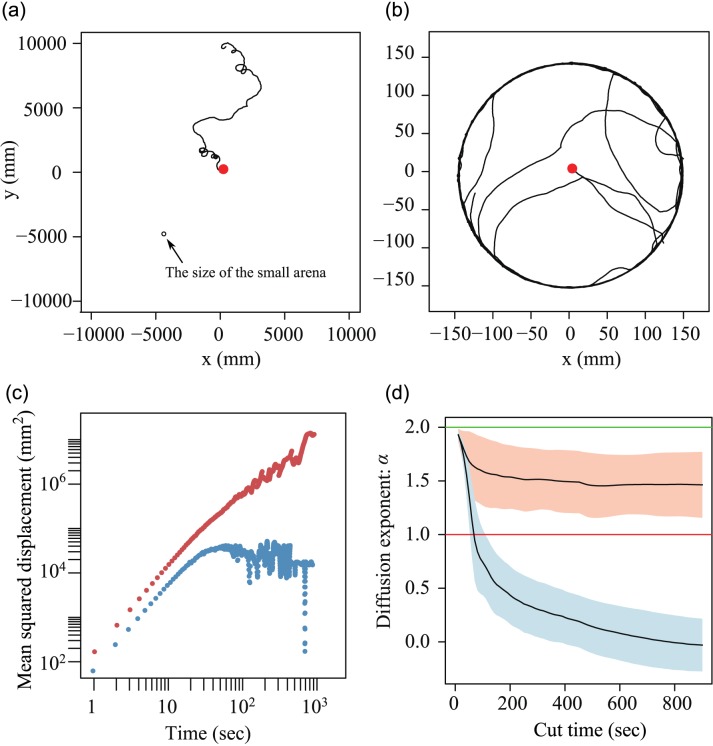
The comparison of diffusive properties between movements on the servosphere and in the small arena. The trajectories of an individual (id: 5) are presented on the servosphere (a) and in the Small arena (b). Mean squared displacements for the trajectories on the servosphere are presented in red whereas those in the small arena are presented in blue (c). The changes in diffusion exponents across analyzed time (d). Lines indicate the means and shadows indicate standard deviations of all analyzed individuals (red: servosphere; blue: Small arena).

On the servosphere, pillbugs diffused apparently freely from the starting points for 1800 sec (diffused distances: mean ± s.d. = 10.5 ± 6.5 m; Fig 5A). Under such conditions, the MSD simply increased with time (Fig 5C). In contrast, in the small arena, pillbugs immediately reached the wall and exhibited wall-following behavior due to thigmotaxis (Fig 5B) [27]. Moreover, because of the limited space for walking, the MSD increased with time and then plateaued (Fig 5C). The diffusion exponent α was robust regardless of the length of time used for regression analysis during the movement on the servosphere, but it varied dramatically during the movement in the small arena (Fig 5D). Thus, it seemed to be difficult to measure α for the movement in the small arena. During the movement on the servosphere, α was 1.46 ± 0.31 (mean ± s.d.; the length of regression time was 900 sec).

**Fig 6:**
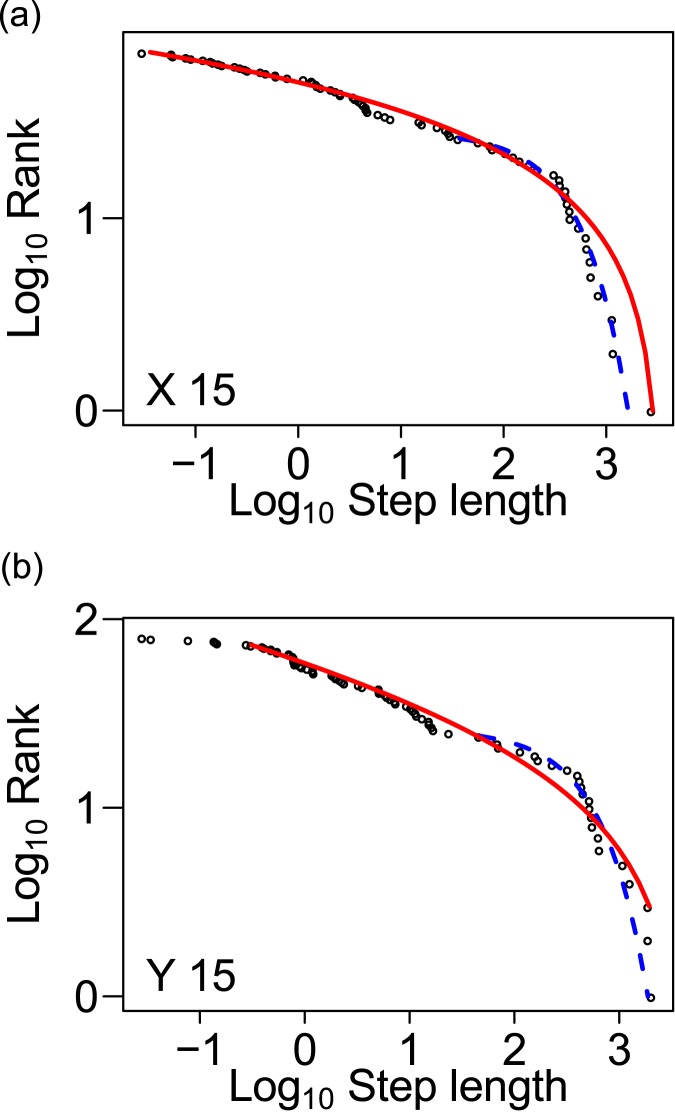
The distribution of step lengths obtained from a representative individual that exhibited truncated Lévy walk pattern. The analysis were conducted for x-axis (a) and y-axis (b) separately. Black dots are observations, red and blue dashed lines are truncated Pareto and competing exponential distributions, respectively, fitted to the data.

Of the 28 individuals analyzed on the servosphere, the movements of 14 individuals (50%) fit the best the TP distribution in both dimensions, 9 individuals (32%) fit the TP in only one dimension, and 5 individuals (18%) were fit to neither distribution (Fig 6, S2 Fig; S1–S3 Tables). None of the movements was best fit to the exponential distribution in either dimension. When approximating the movements to the truncated Lévy walk, the Lévy exponent μ was estimated to 1.38 ± 0.29 (mean ± s.d.; *n* = 16) for x-axis and 1.65 ± 0.57 (mean ± s.d.; *n* = 21) for y-axis.

## Discussion

Here, we present a novel tracking system to measure the inherent free-walking behavior of small animals. This novel system was developed based on the OTM mechanism, but it was improved in points that will be mentioned in the following discussion. We tested the potential of the servosphere to measure the diffusive properties of small animals by comparing free-walking behavior of pillbugs on a servosphere with those in flat arenas. Individual pillbugs showed similar diffusive characteristics both on the servosphere and large arena that simulated an open space. Compared with small arena, which is often used for tracking small animals in the laboratory, our system offered a better assessment of how pillbugs perform anomalous diffusion by enabling a robust measure of diffusive properties. This highly diffusive walking behavior of pillbugs is consistent with the previous observation that they often move forward in a straight line in an open field [28]. Thus, our system would be suitable to explore diffusion characteristics of small animals.

Many studies have shown that Lévy-like, multi-scale search behavior is displayed by a wide range of animals, including humans, and it was observed even in trails of trace fossils [4,24,29–31]. There are several hypotheses about the underlying mechanism of the Lévy walk. According to one hypothesis Lévy walk arises from interactions of animals with external factors, i.e., the environment [32]. The other hypothesis, which has been supported by increasing evidence, states that Lévy walk is shaped by intrinsic factors of the animals’ neuronal and/or physiological mechanisms, [2,5,33]. In this study, we confirmed that the Lévy-like behavior in pillbugs on the servosphere was not triggered by any external factors (Fig 6). This result suggests that the multi-scale walking behavior displayed by pillbugs is elicited by their intrinsic factors. Our system provides a unique opportunity to test the existence of the multi-scale searching behavior in small animals across various taxa.

Tracking the free-walking behavior using servospheres such as ANTAM has some fundamental advantages over other locomotion compensator systems. First, our system does not require any external manipulation of the body of test animals. For example, in most locomotion compensator systems (e.g., trackballs [19,21] and the insect-controlled robot [34]), test animals need to be tethered or cemented above the ball to keep them at the same place. Such treatments can restrict the degree of freedom in their movement, especially in turning motion [21]. Second, compared with previous locomotion compensator systems using servospheres, our system has an advantage in being free from the need of a reflector [17,35]. We should note that such systems without the need of a reflector have been developed by Sakuma [15] and van Tilborg et al. [13], which explored insects’ orientation to the olfactory signals. Tracking without any external manipulation could be the necessary condition for measuring free-walking behavior, concerning the potential effect of external manipulations on the behavior of test animals [36].

Another advantage of our system is that static electricity, which causes small test animals to be blown suddenly out of the measurement range, is removed by the static electricity removal brush. A similar problem of static electricity has also been reported by van Tilborg et al. in another OTM device, LC 100 of Syntech Inc. [13]. They reported that static electricity adversely affected the motion of target insects in approximately 5% of the trials and that an effort to reduce the influence of static electricity by placing the device in a highly humid environment in turn caused dust and garbage to attach to the ball, eventually disrupting the image analysis. The use of the removal brush was the only solution as long as we tested (e.g. highly humid environment, other materials for a sphere and sponges, stuffing wires in a sphere, washing a sphere with water at the beginning of each trial, or contacting a sphere and a device with earth wires).

In addition, because our system uses the PSR mechanism to transmit the driving force, not only pillbugs but also other insects can be targeted, even with relatively greater moving speed and a more rapid change in acceleration. In particular, the technical challenge we faced during motion compensation is how to cancel the inertial force following the acceleration change of the sphere. One solution is to create a hardware that is highly responsive and with a large output torque, i.e., to use a motor. However, even if such a motor is used, when the frictional force at the contact portion with the sphere is low, slippage occurs and it becomes difficult to realize high responsiveness. Generally, since such a motor is large in size, the device itself must be large in size. The other solution, the use of the PSR mechanism which was implemented herein, is superior because the size of the apparatus remains compact while the problem of the increased number of independently driven motors is solved.

Several factors may be involved in the observed difference in moving speed and turning angles among the tracking conditions. First, previous studies of animal tracking devices [21,37] noted that the accuracy of measurement depends on the types of sensors used. They compared results using a computer mouse sensor with those using a computer-vision-based tracking system, and found that the former showed apparent increases in the walking speed and the straightness of their paths, possibly attributed to a reduction in sensor sensitivity that systematically occurred only in the former. Our results, using only the mouse sensor for the servosphere, are partially consistent with this observation, as individual pillbugs moved at higher speed and made turns at sharper angles on the servosphere than in the small arena, but not in the large arena. Second, the surface of each tracking environment was different (paper, styrene, and acrylic for large arena, small arena, and servosphere, respectively), which might have affected the moving speed of individuals. Third, among tracking conditions, there is a difference in visual stimulation such as the grid lines in the large arena or walls in the small arena, which could also affect the walking characteristics. In addition, several features of our servosphere, such as inevitable mechanical vibration and the spherical shape of the tracking floor, could also have affected their movement characteristics. Thus, our system is not suitable to measure parameters such as speed of small animals. Yet, the results confirmed that our system is adequate to measure the diffusive characteristics of individuals (Fig 4), which is an essential parameter to determine the efficiency of the searching strategy [3].

Although many studies exploited the OTM systems, most of them have focused on the response to real or virtual external stimuli, whereas how animals move on OTM without stimuli remained largely unexplored. In this study, we demonstrated that the servosphere ANTAM is useful to measure the inherent movement patterns of small animals, especially in diffusiveness. Further studies are warranted to confirm the validity of our system in a wider variety of animals. By comparing the inherent movement patterns within and among species, our system provides an innovative step for investigating the movement patterns in animals.

## Supporting information

S1 Fig The histograms of the data of mean speed, mean angle and max speed. (PDF)

S2 Fig The distribution of step lengths for all individuals. Combinations of a letter and a number indicates the analyzed axis and the individuals. Black dots are observations, red and blue dashed lines are truncated Pareto and competing exponential distributions, respectively, fitted to the data.
(PDF)

S1 Table Maximum likelihood estimation analysis results for the individuals whose trajectories are determined as TP for both x and y axis. TP is truncated Pareto, Exp is exponential; OOM is order of magnitude.
(PDF)

S2 Table Maximum likelihood estimation analysis results for the individuals whose trajectories are determined as TP for either x or y axis. TP is truncated Pareto, Exp is exponential; OOM is order of magnitude.
(PDF)

S3 Table Maximum likelihood estimation analysis results for the individuals whose trajectories are determined as unclassified. TP is truncated Pareto, Exp is exponential; OOM is order of magnitude.
(PDF)

S1 Datasets The data and trajectories obtained in our experiments. (ZIP)
