# Supplementary figures and images for: Anomalous diffusion on the servosphere: A potential tool for detecting inherent organismal movement patterns
Source: PLoS One. 2017 Jun 1;12(6):e0177480. doi: 10.1371/journal.pone.0177480 (PMC5453419; doi:10.1371/journal.pone.0177480)

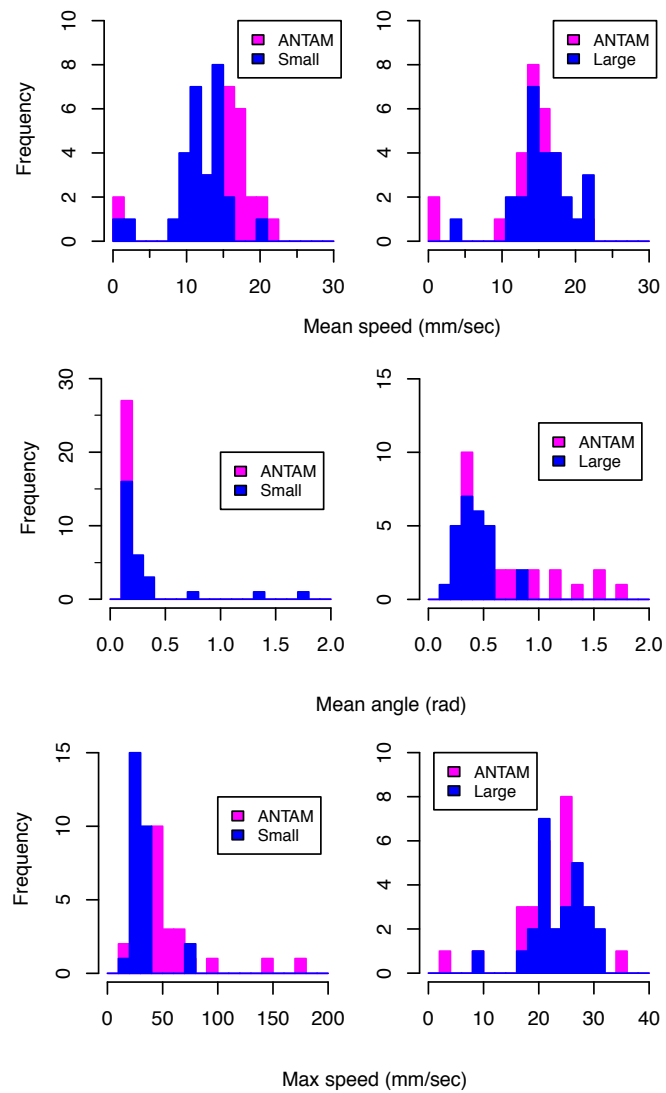

S1 Fig. The histograms of the data of mean speed, mean angle and max speed.

Supplement: S1 Fig — (PDF) [file pone.0177480.s001.pdf]

5\_01

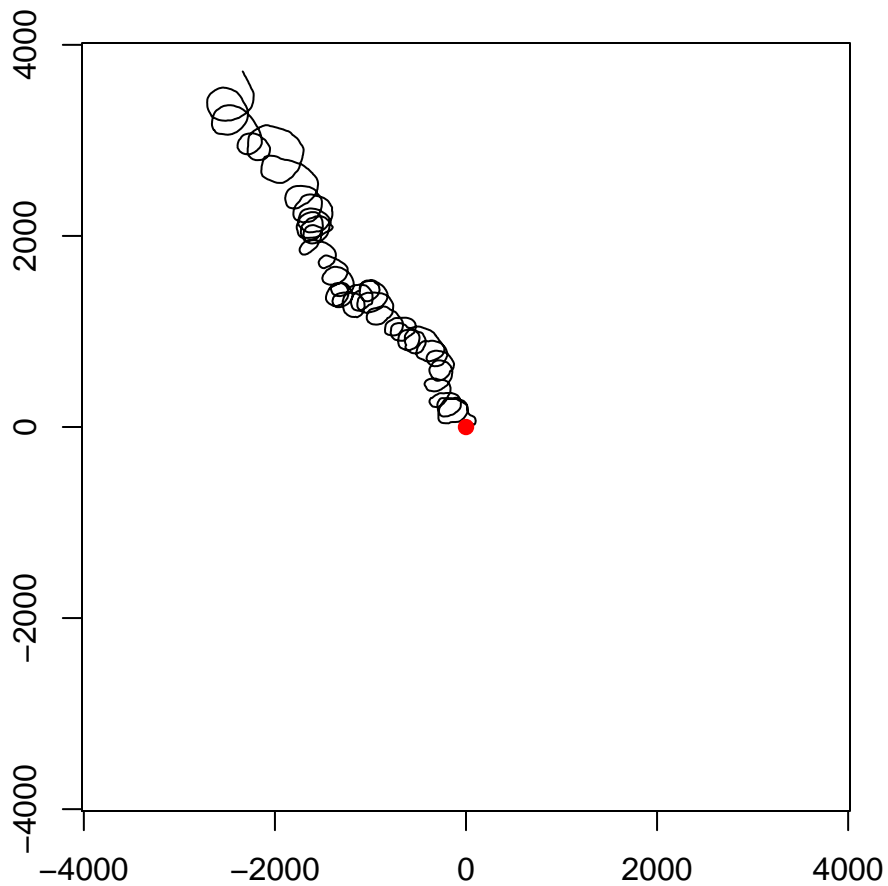

Supplement: S1 Datasets — (ZIP) [file pone.0177480.s006.zip › ANTAM data/trajectories/ANTAM (5FPS)/ANTAM_FPS5_01.pdf]

5\_02

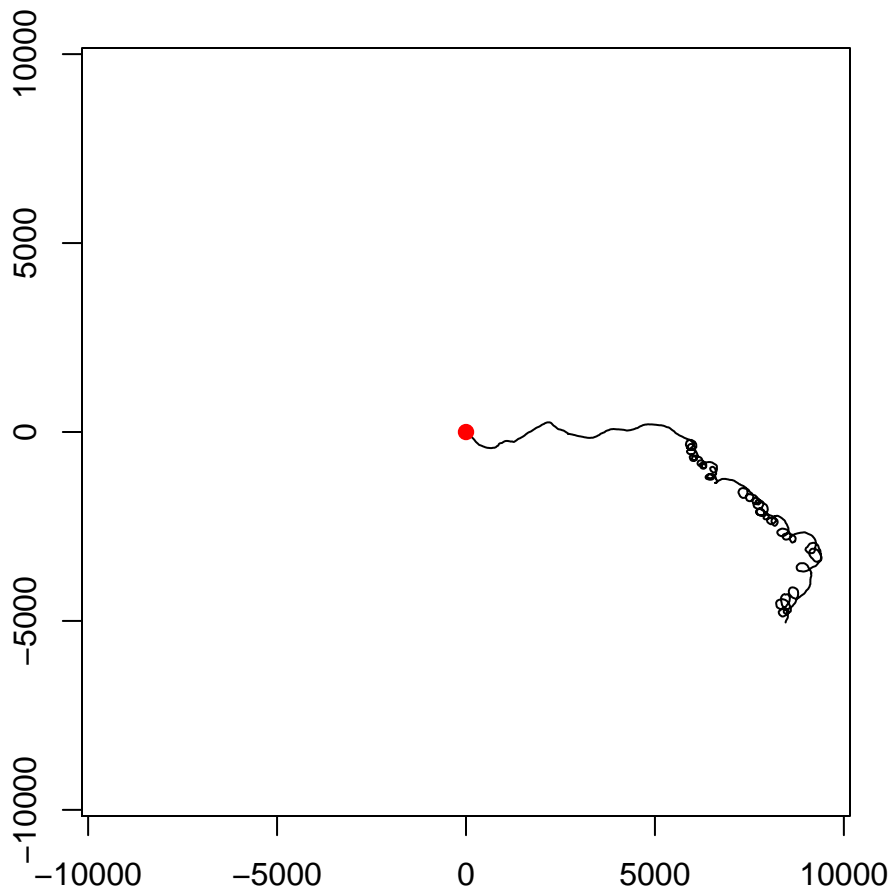

Supplement: S1 Datasets — (ZIP) [file pone.0177480.s006.zip › ANTAM data/trajectories/ANTAM (5FPS)/ANTAM_FPS5_02.pdf]

5\_03

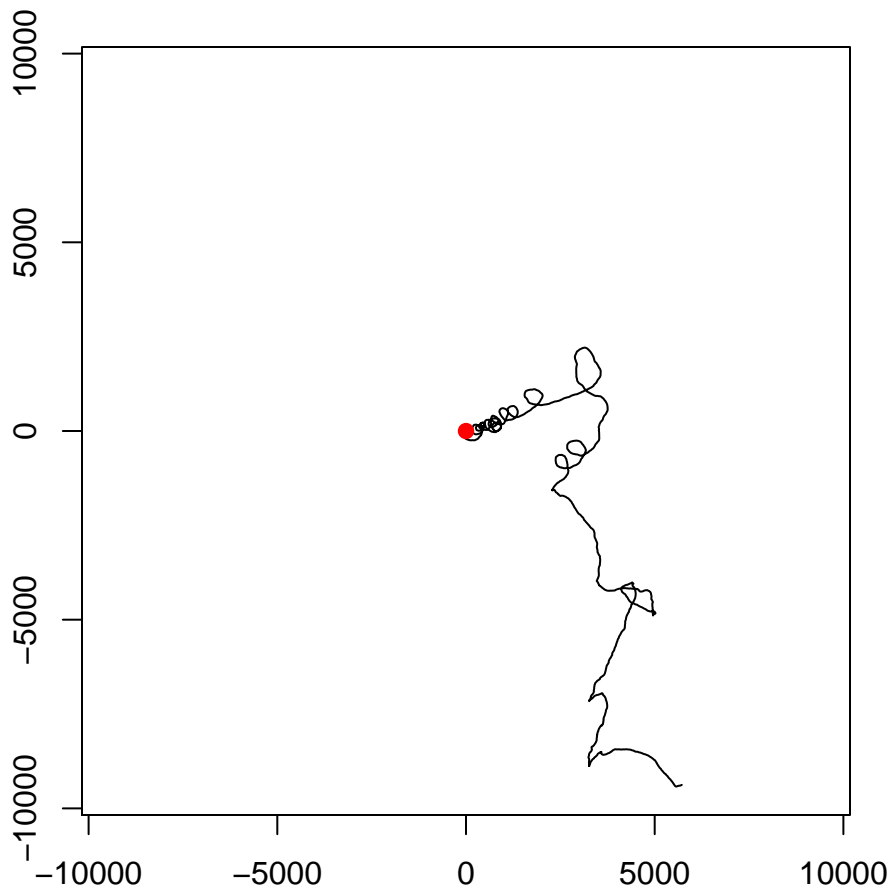

Supplement: S1 Datasets — (ZIP) [file pone.0177480.s006.zip › ANTAM data/trajectories/ANTAM (5FPS)/ANTAM_FPS5_03.pdf]

5\_05

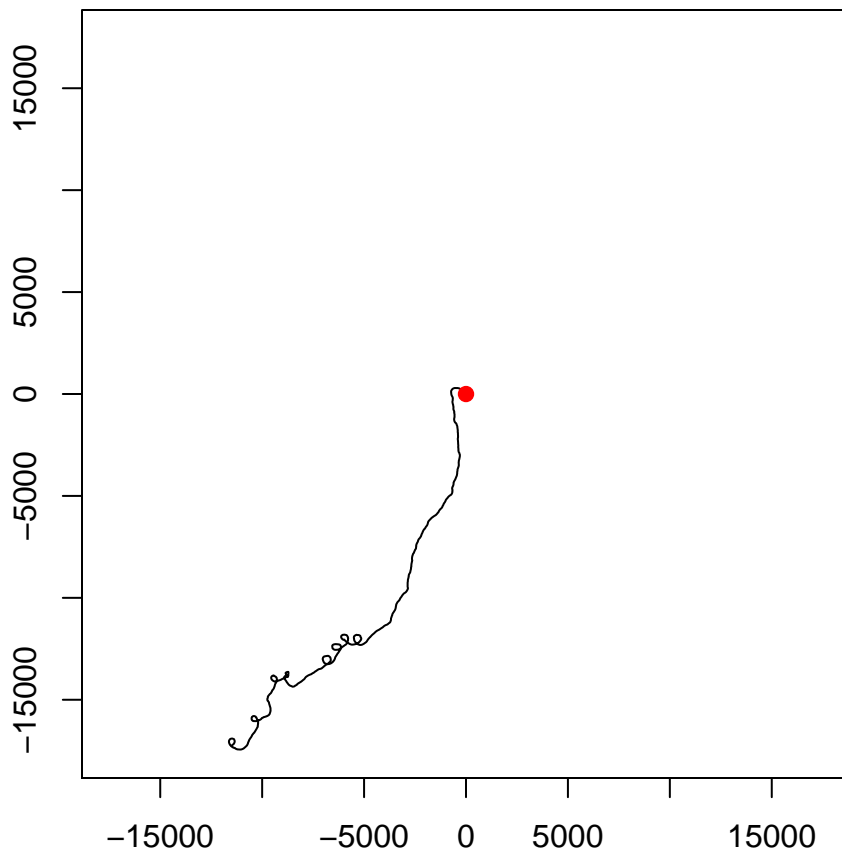

Supplement: S1 Datasets — (ZIP) [file pone.0177480.s006.zip › ANTAM data/trajectories/ANTAM (5FPS)/ANTAM_FPS5_05.pdf]

5\_06

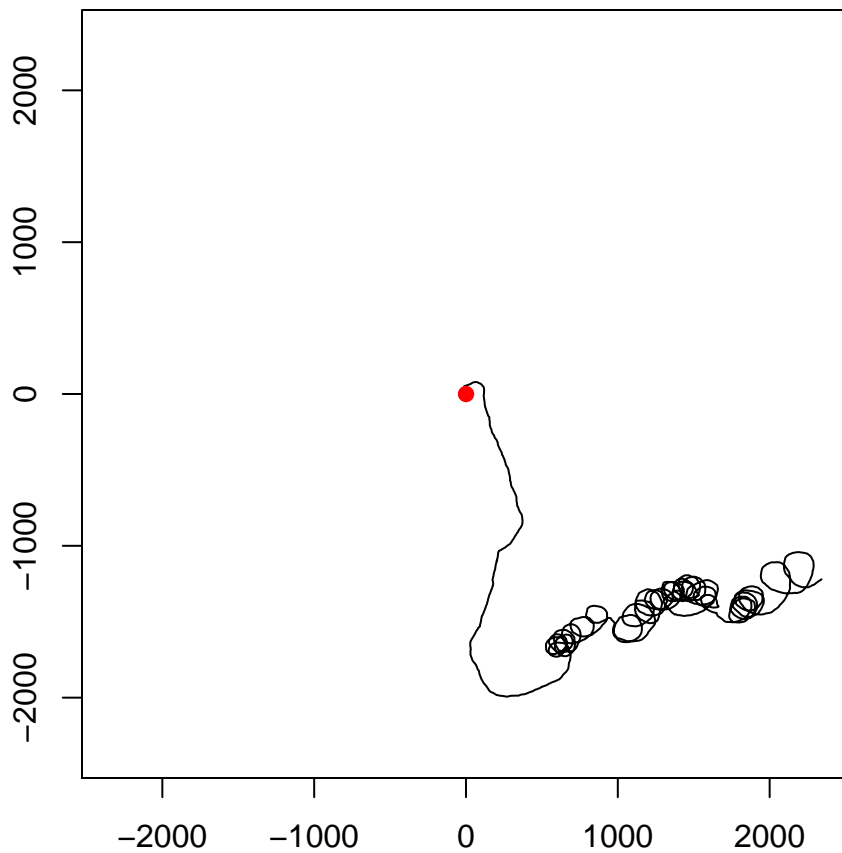

Supplement: S1 Datasets — (ZIP) [file pone.0177480.s006.zip › ANTAM data/trajectories/ANTAM (5FPS)/ANTAM_FPS5_06.pdf]

**5\_07**

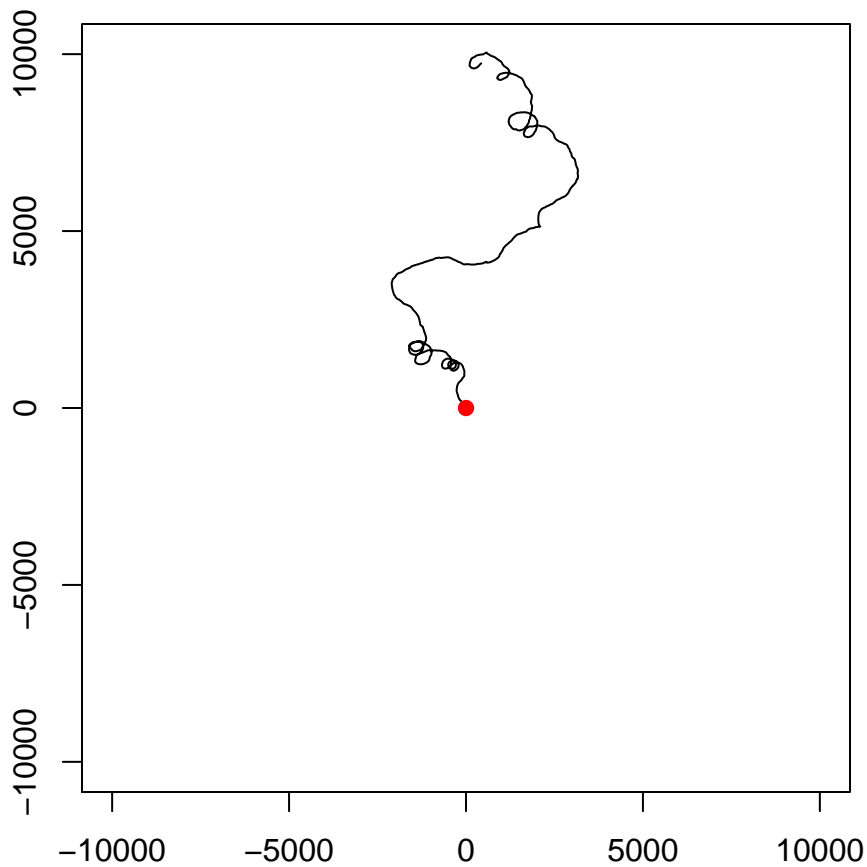

Supplement: S1 Datasets — (ZIP) [file pone.0177480.s006.zip › ANTAM data/trajectories/ANTAM (5FPS)/ANTAM_FPS5_07.pdf]

5\_08

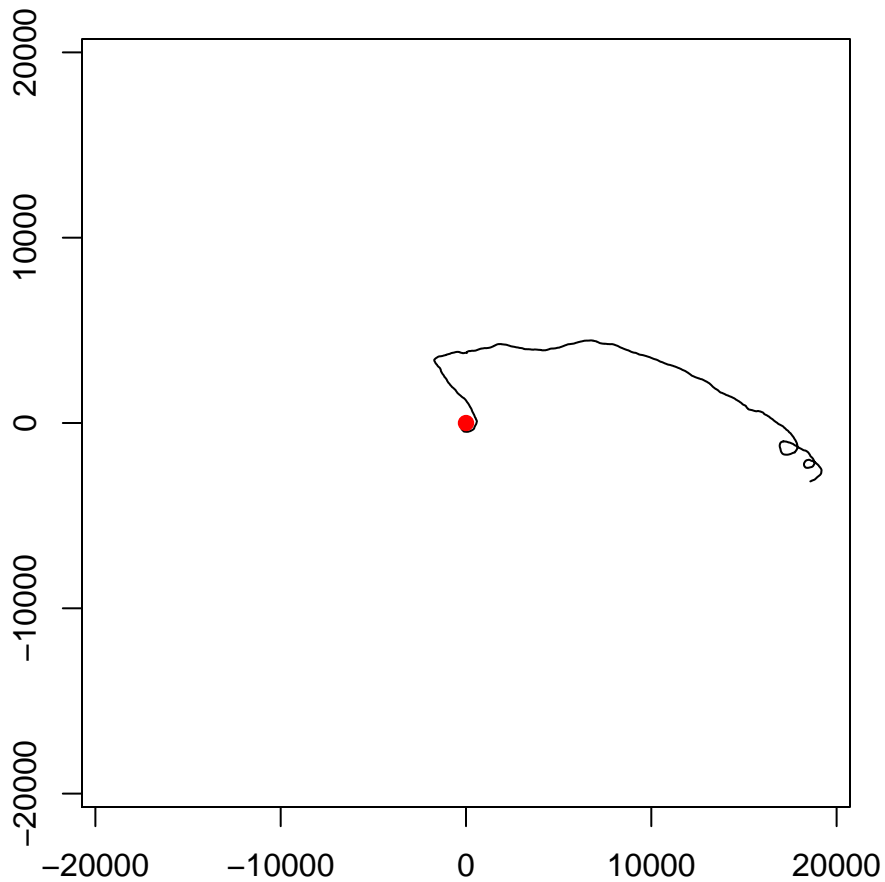

Supplement: S1 Datasets — (ZIP) [file pone.0177480.s006.zip › ANTAM data/trajectories/ANTAM (5FPS)/ANTAM_FPS5_08.pdf]

5\_09

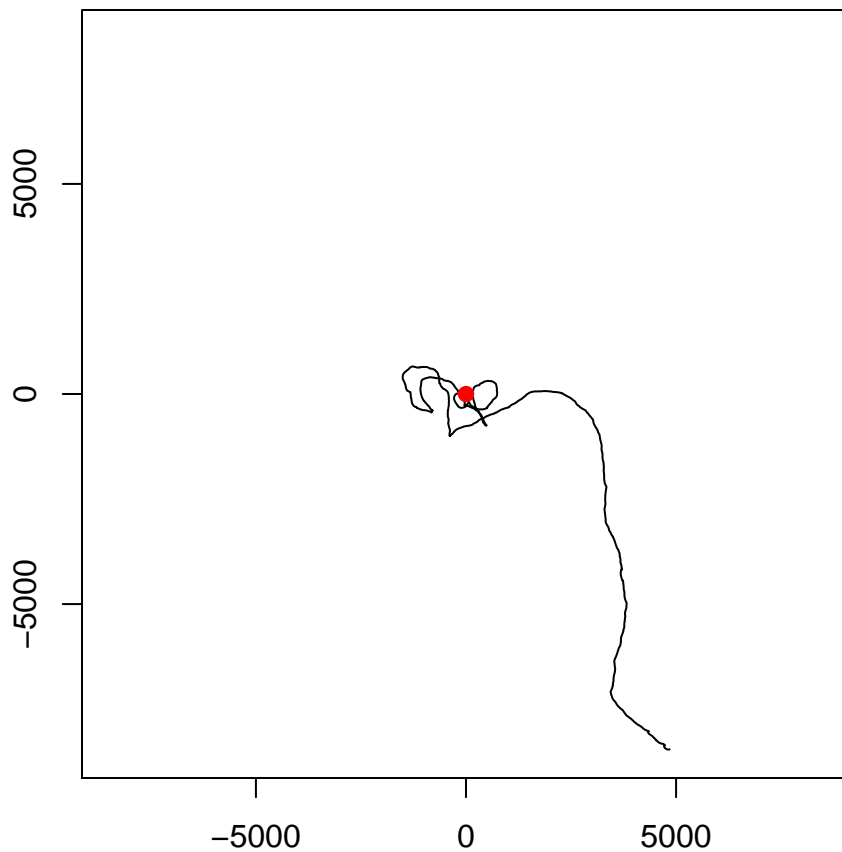

Supplement: S1 Datasets — (ZIP) [file pone.0177480.s006.zip › ANTAM data/trajectories/ANTAM (5FPS)/ANTAM_FPS5_09.pdf]

5\_10

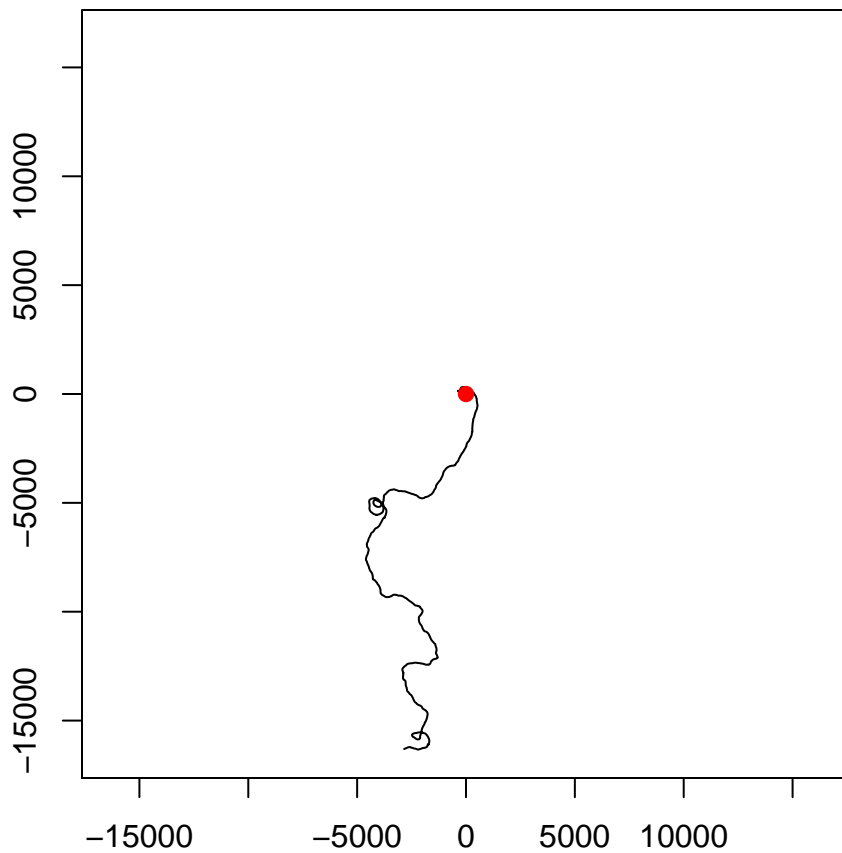

Supplement: S1 Datasets — (ZIP) [file pone.0177480.s006.zip › ANTAM data/trajectories/ANTAM (5FPS)/ANTAM_FPS5_10.pdf]

5\_11

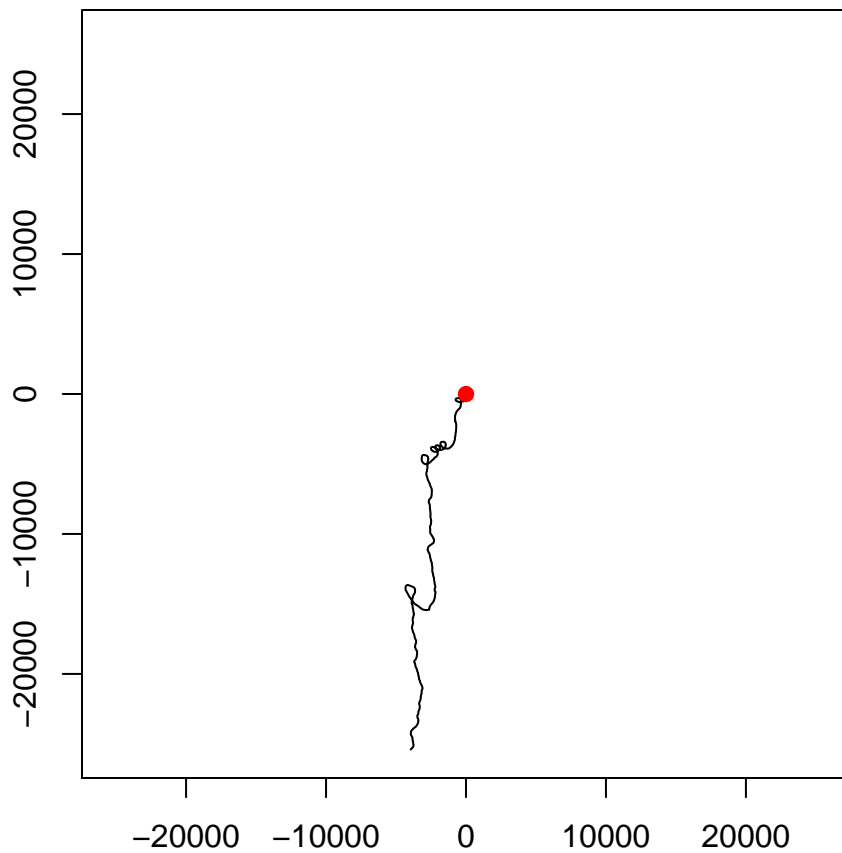

Supplement: S1 Datasets — (ZIP) [file pone.0177480.s006.zip › ANTAM data/trajectories/ANTAM (5FPS)/ANTAM_FPS5_11.pdf]

5\_12

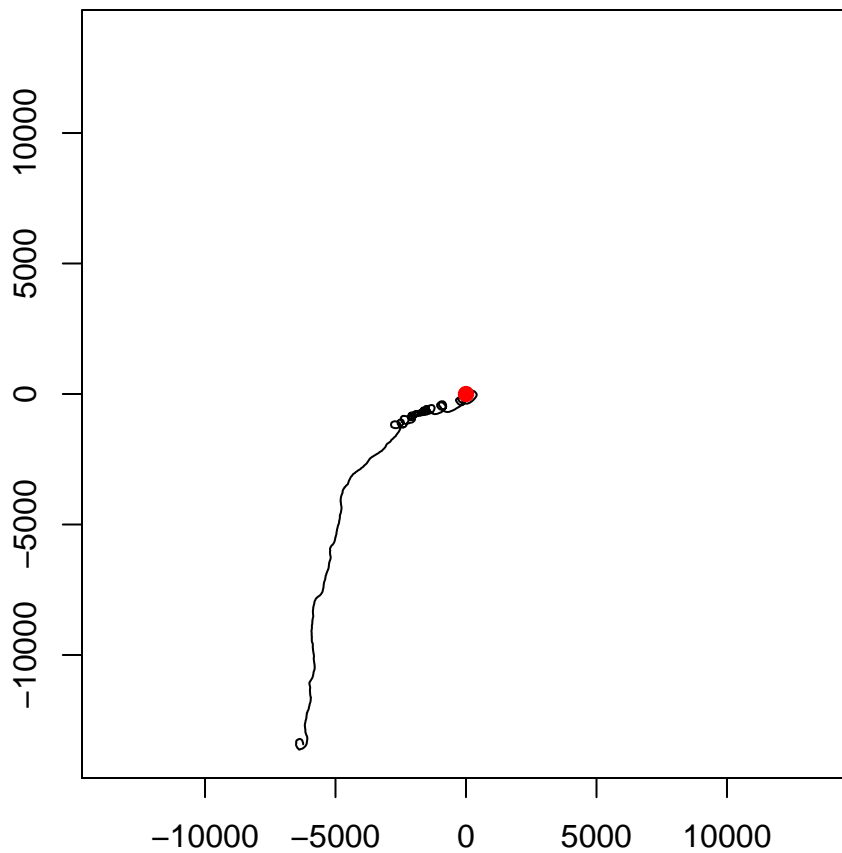

Supplement: S1 Datasets — (ZIP) [file pone.0177480.s006.zip › ANTAM data/trajectories/ANTAM (5FPS)/ANTAM_FPS5_12.pdf]

5\_13

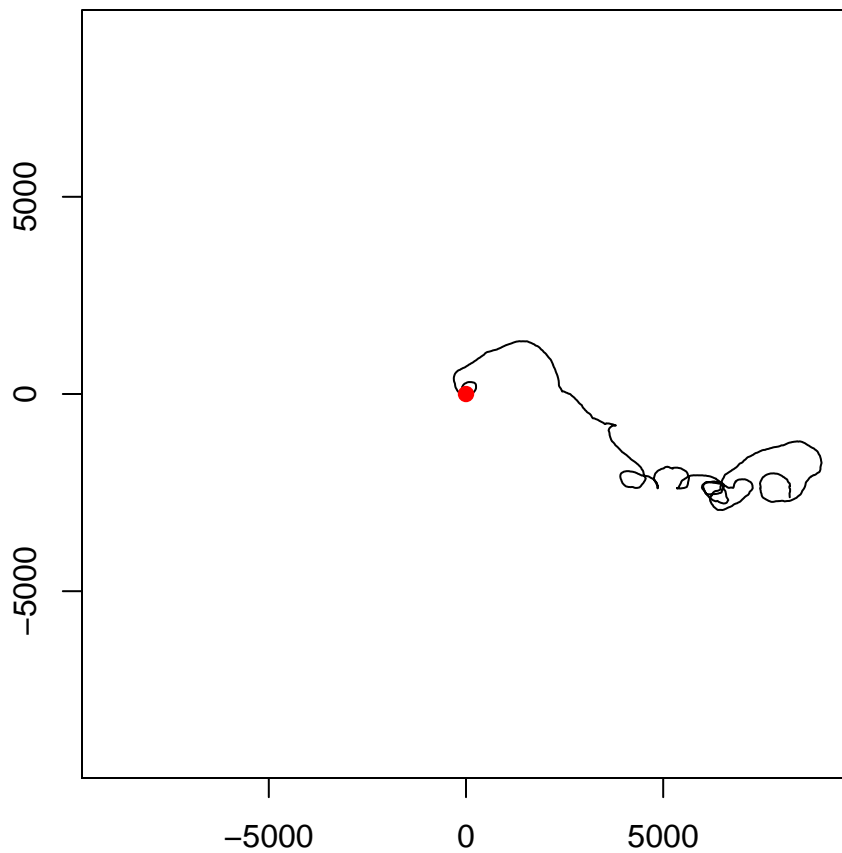

Supplement: S1 Datasets — (ZIP) [file pone.0177480.s006.zip › ANTAM data/trajectories/ANTAM (5FPS)/ANTAM_FPS5_13.pdf]

5\_14

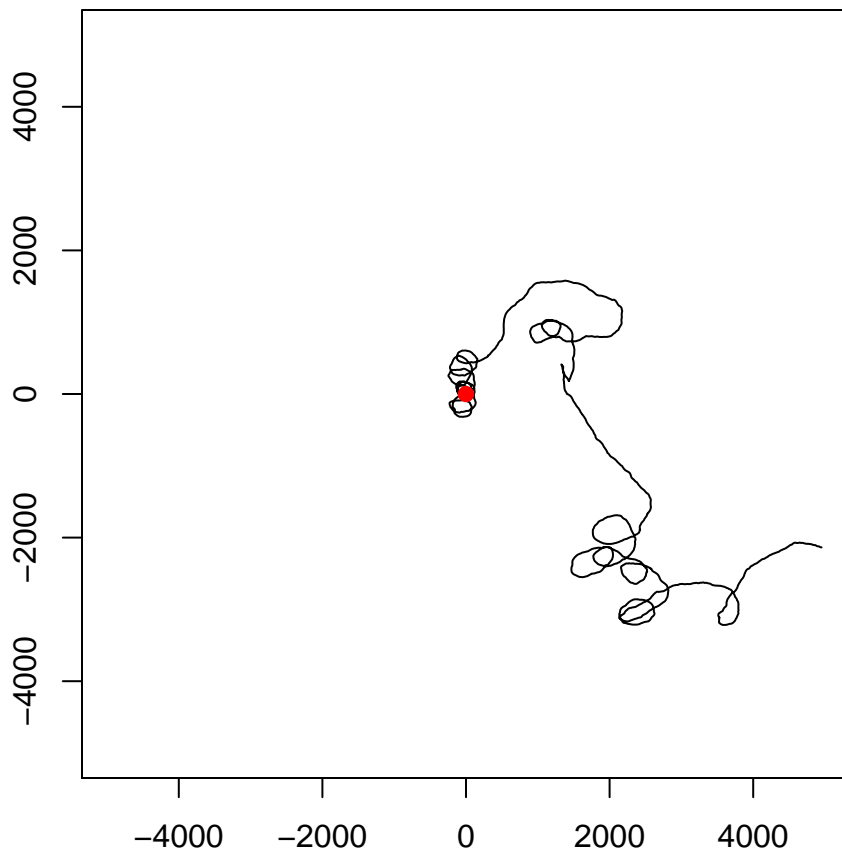

Supplement: S1 Datasets — (ZIP) [file pone.0177480.s006.zip › ANTAM data/trajectories/ANTAM (5FPS)/ANTAM_FPS5_14.pdf]

5\_15

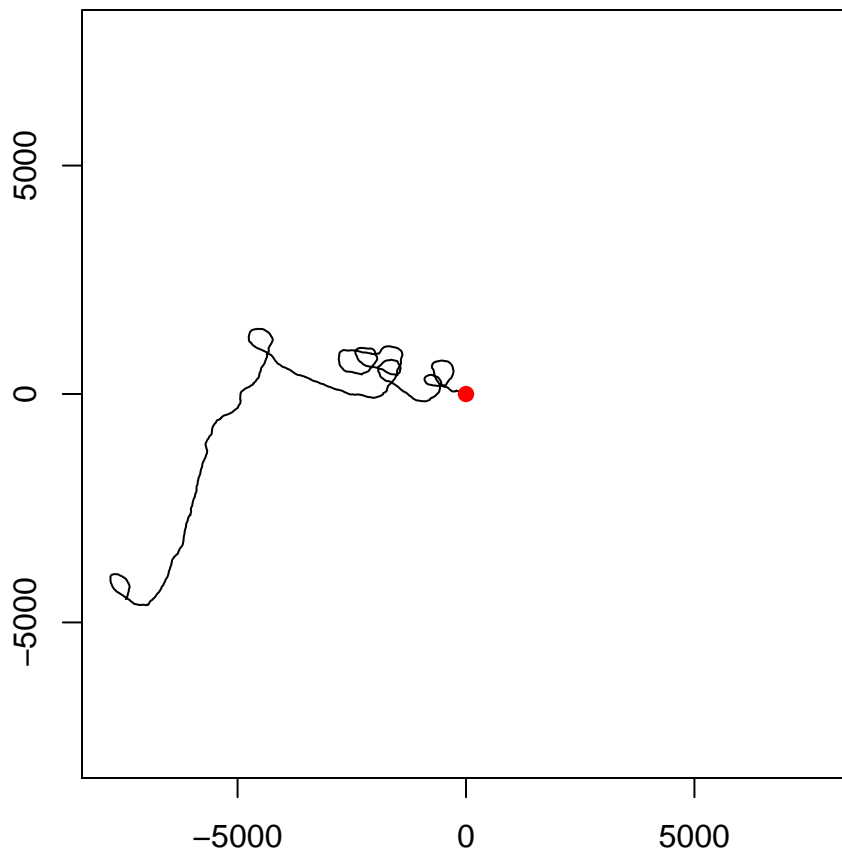

Supplement: S1 Datasets — (ZIP) [file pone.0177480.s006.zip › ANTAM data/trajectories/ANTAM (5FPS)/ANTAM_FPS5_15.pdf]

5\_16

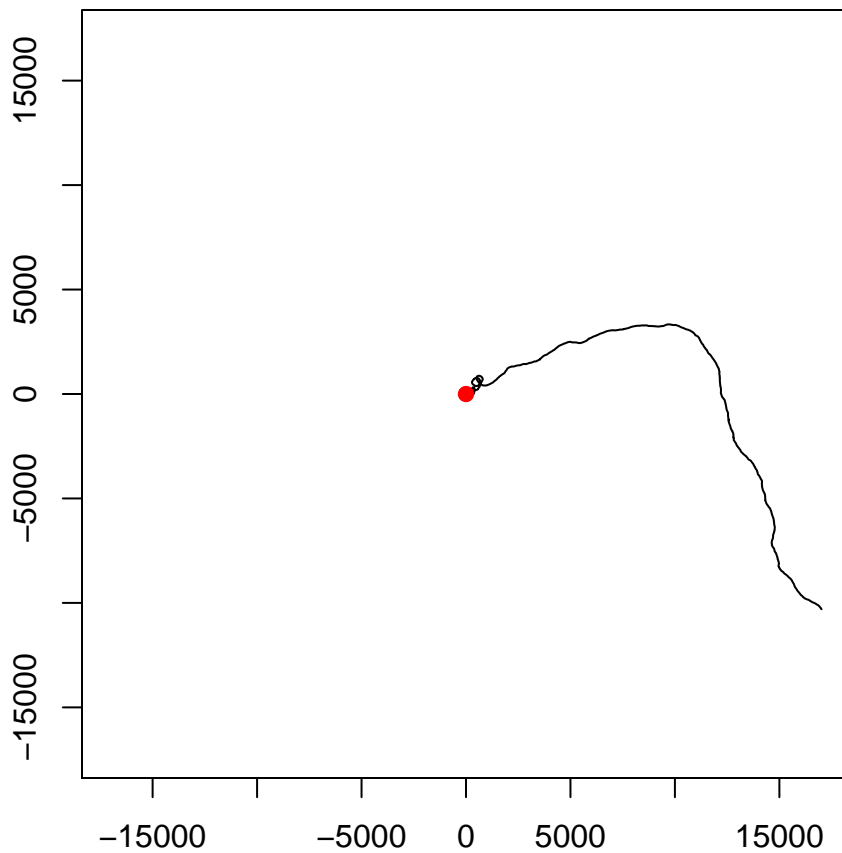

Supplement: S1 Datasets — (ZIP) [file pone.0177480.s006.zip › ANTAM data/trajectories/ANTAM (5FPS)/ANTAM_FPS5_16.pdf]

5\_17

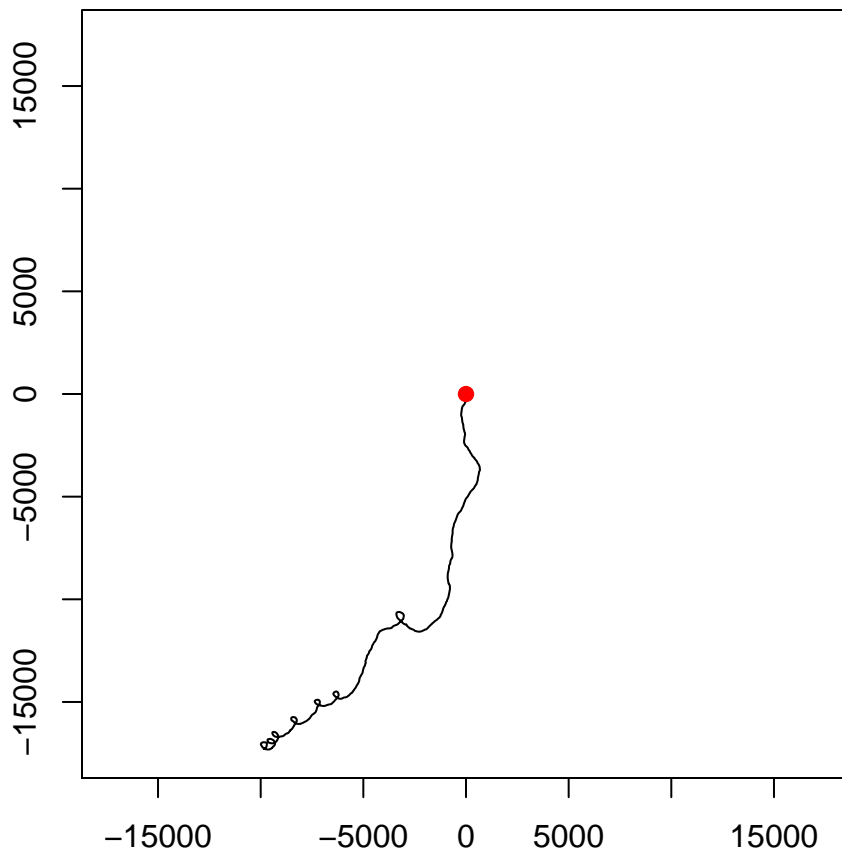

Supplement: S1 Datasets — (ZIP) [file pone.0177480.s006.zip › ANTAM data/trajectories/ANTAM (5FPS)/ANTAM_FPS5_17.pdf]

5\_18

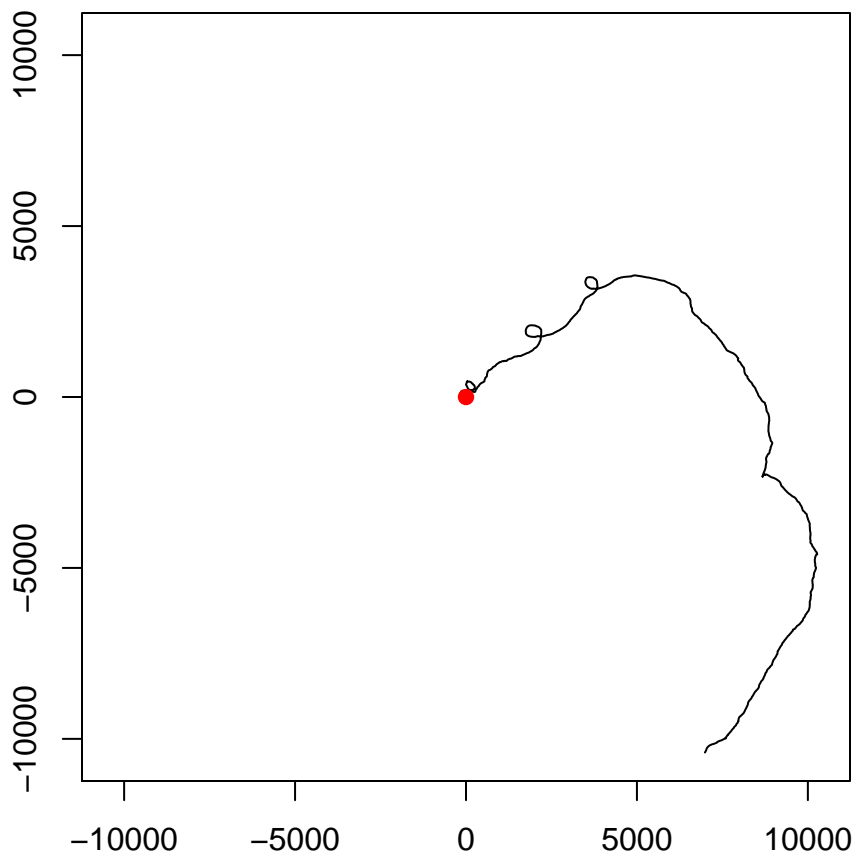

Supplement: S1 Datasets — (ZIP) [file pone.0177480.s006.zip › ANTAM data/trajectories/ANTAM (5FPS)/ANTAM_FPS5_18.pdf]

5\_19

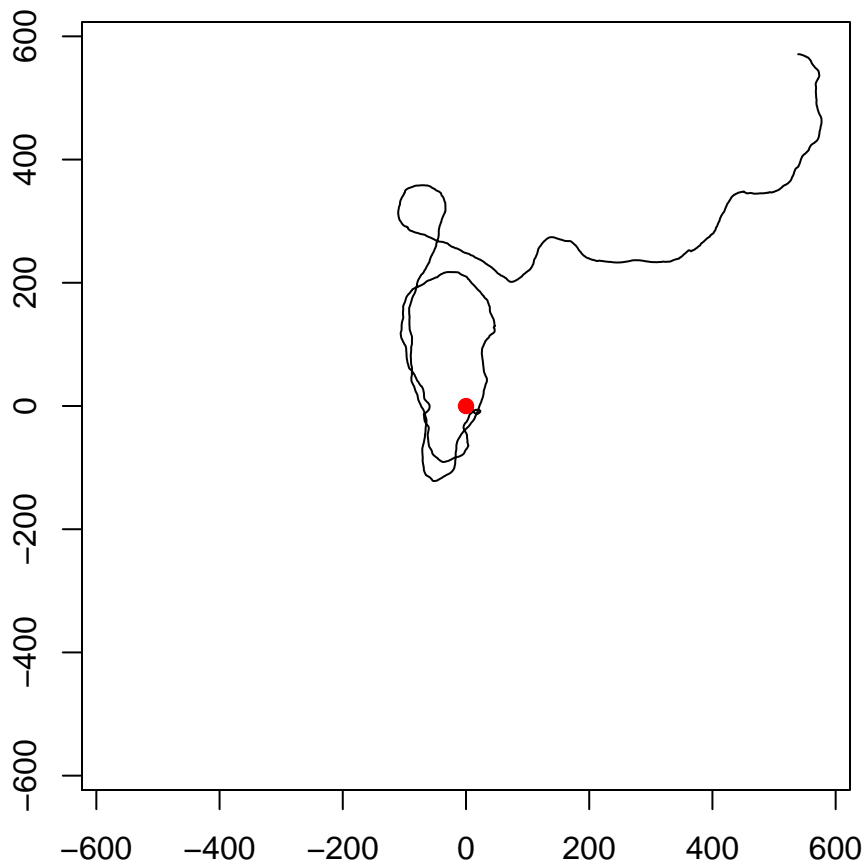

Supplement: S1 Datasets — (ZIP) [file pone.0177480.s006.zip › ANTAM data/trajectories/ANTAM (5FPS)/ANTAM_FPS5_19.pdf]

5\_20

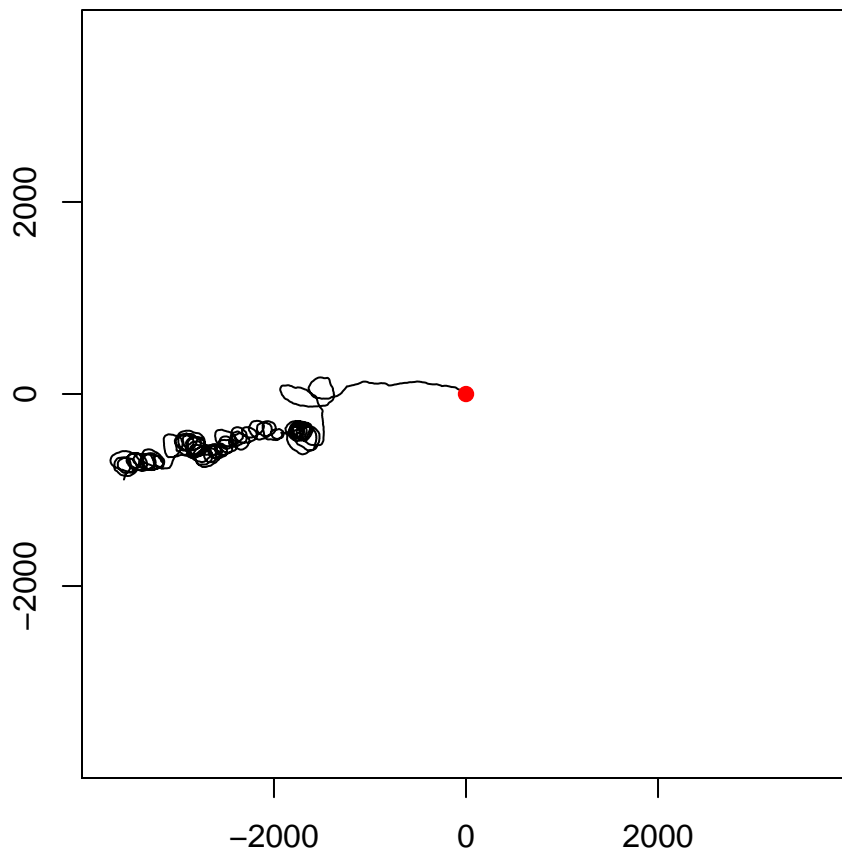

Supplement: S1 Datasets — (ZIP) [file pone.0177480.s006.zip › ANTAM data/trajectories/ANTAM (5FPS)/ANTAM_FPS5_20.pdf]

5\_21

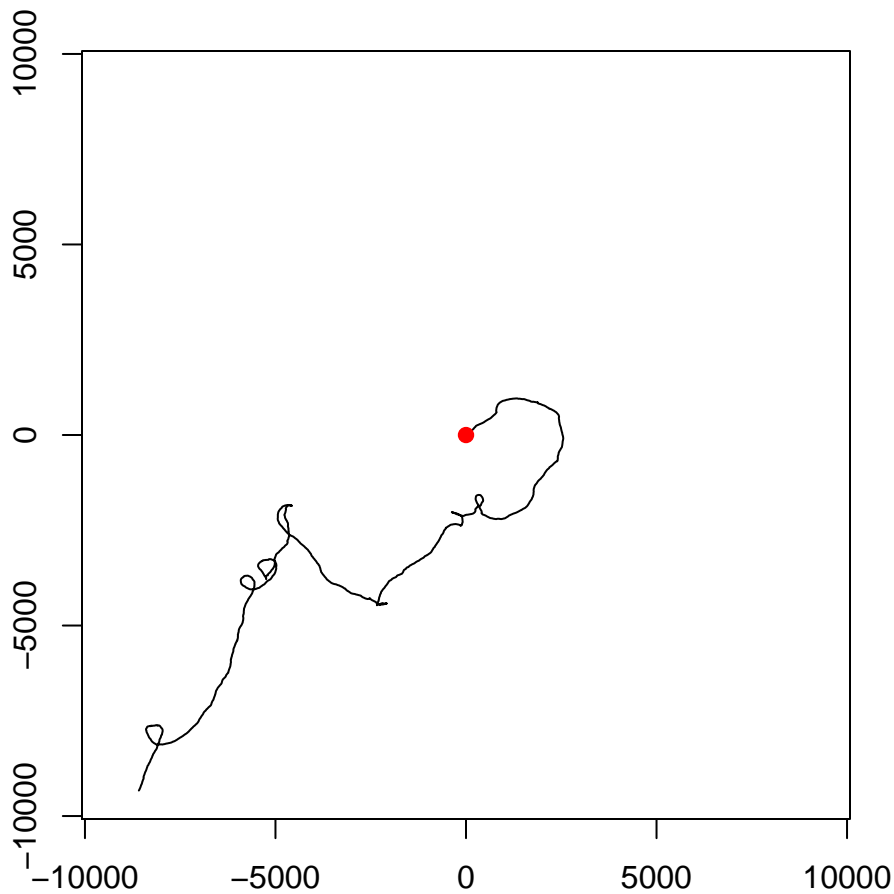

Supplement: S1 Datasets — (ZIP) [file pone.0177480.s006.zip › ANTAM data/trajectories/ANTAM (5FPS)/ANTAM_FPS5_21.pdf]

5\_22

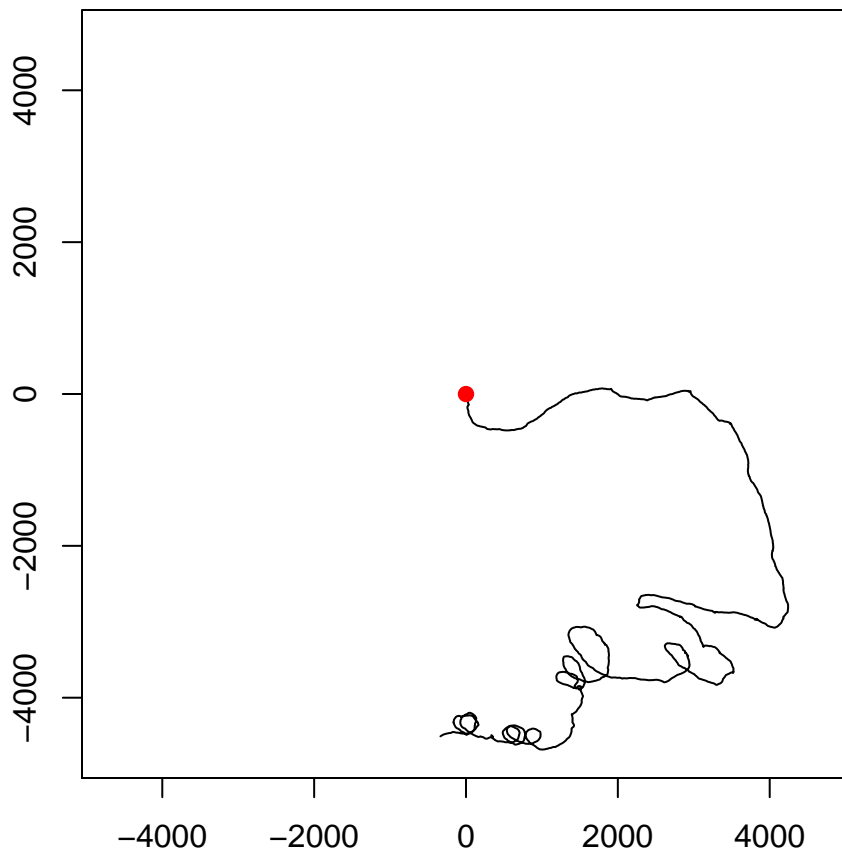

Supplement: S1 Datasets — (ZIP) [file pone.0177480.s006.zip › ANTAM data/trajectories/ANTAM (5FPS)/ANTAM_FPS5_22.pdf]

5\_23

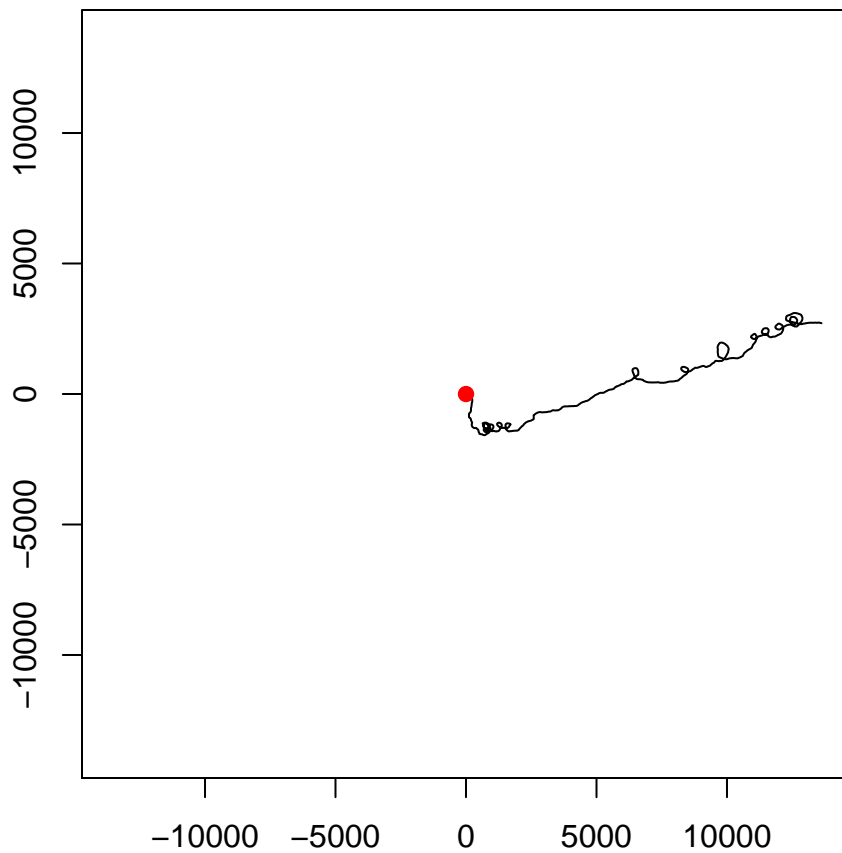

Supplement: S1 Datasets — (ZIP) [file pone.0177480.s006.zip › ANTAM data/trajectories/ANTAM (5FPS)/ANTAM_FPS5_23.pdf]

5\_24

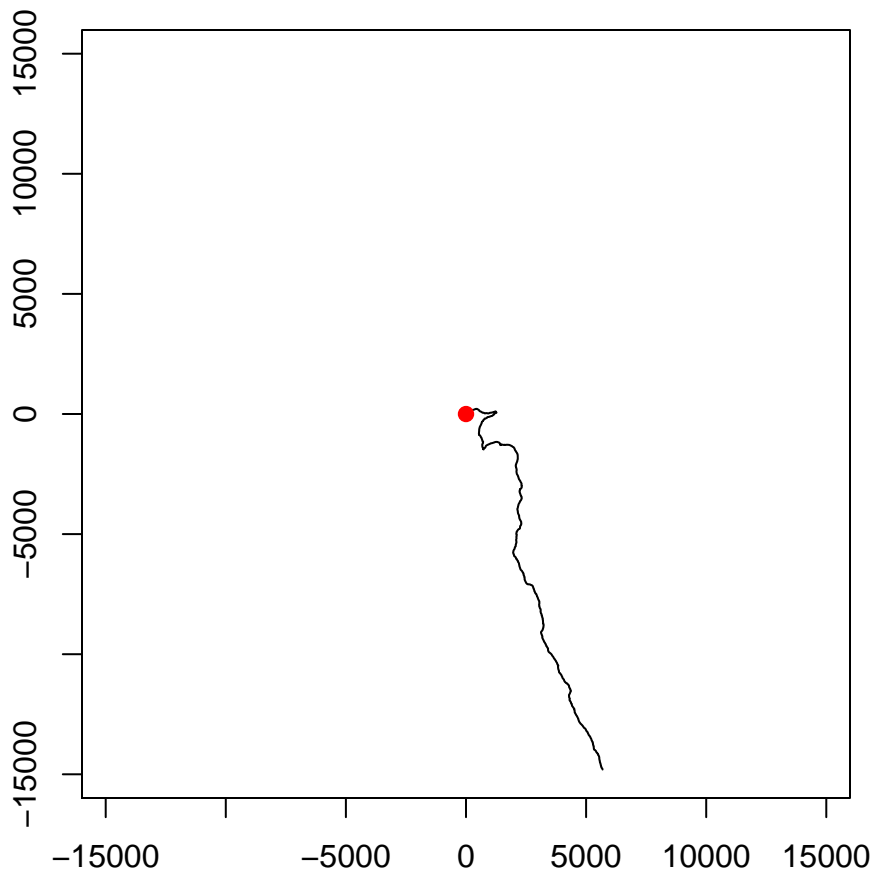

Supplement: S1 Datasets — (ZIP) [file pone.0177480.s006.zip › ANTAM data/trajectories/ANTAM (5FPS)/ANTAM_FPS5_24.pdf]

5\_25

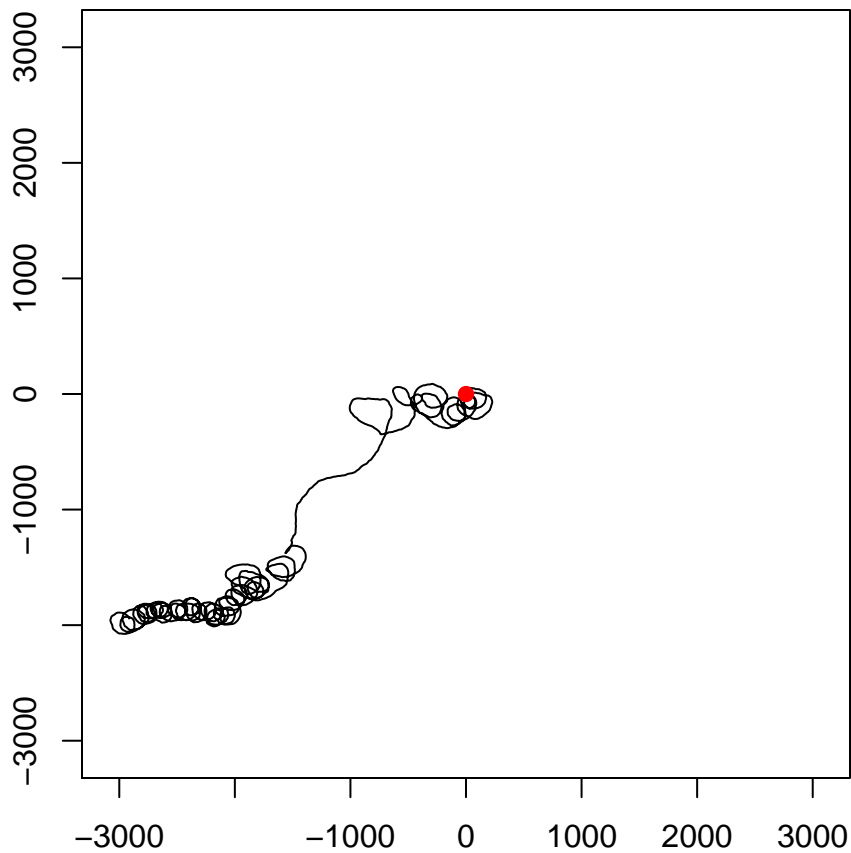

Supplement: S1 Datasets — (ZIP) [file pone.0177480.s006.zip › ANTAM data/trajectories/ANTAM (5FPS)/ANTAM_FPS5_25.pdf]

5\_26

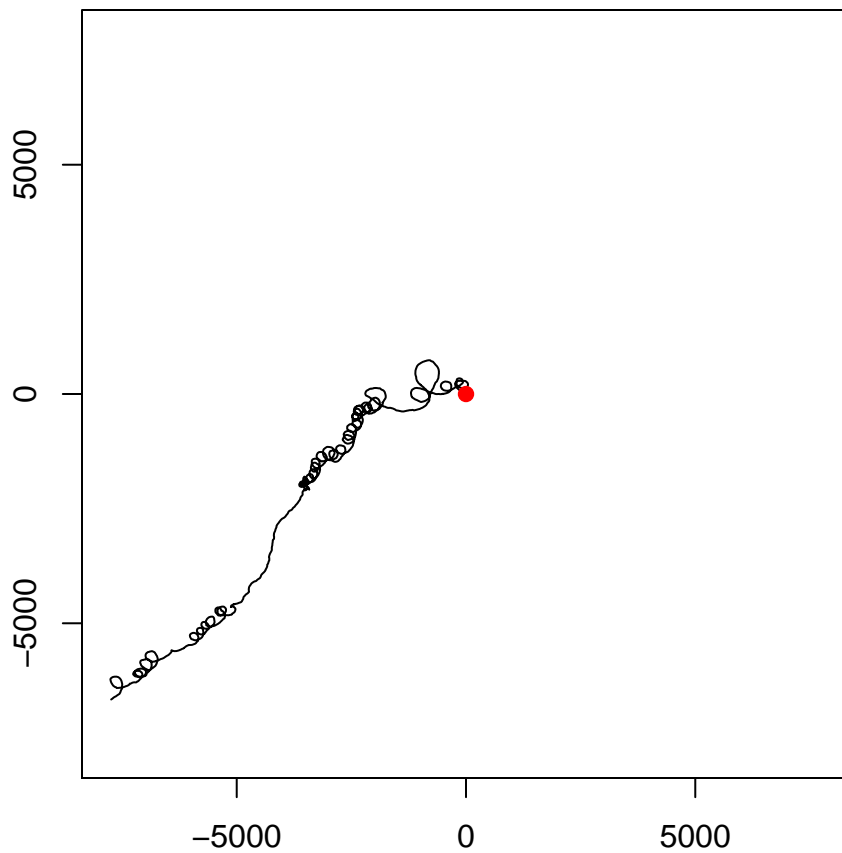

Supplement: S1 Datasets — (ZIP) [file pone.0177480.s006.zip › ANTAM data/trajectories/ANTAM (5FPS)/ANTAM_FPS5_26.pdf]

5\_27

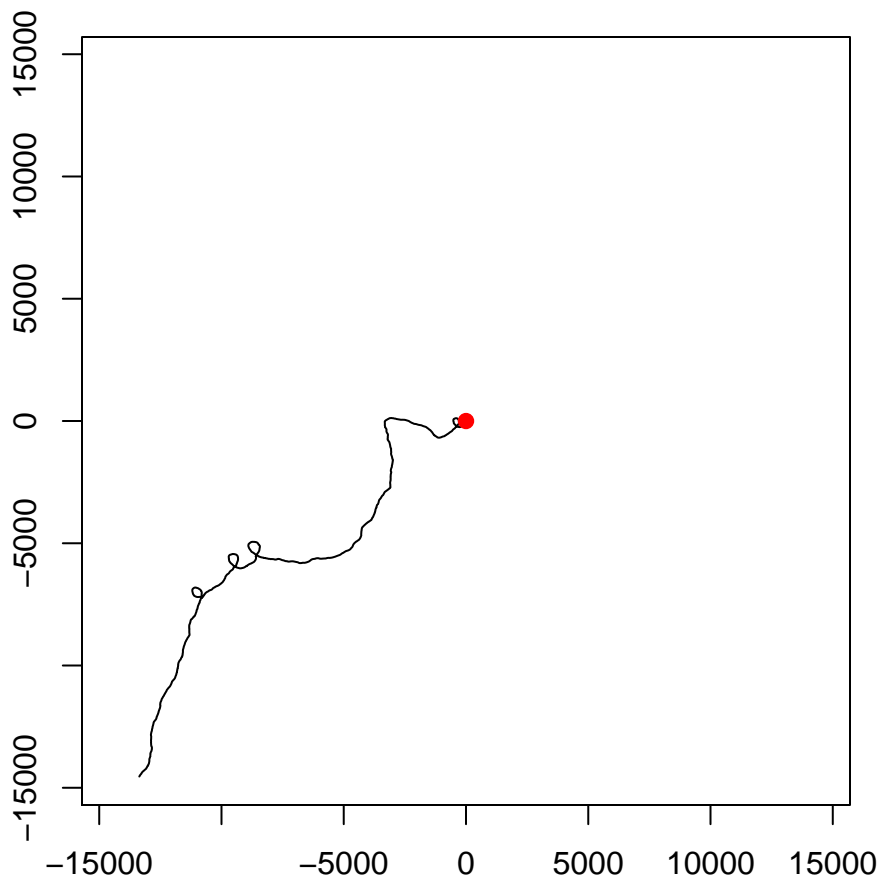

Supplement: S1 Datasets — (ZIP) [file pone.0177480.s006.zip › ANTAM data/trajectories/ANTAM (5FPS)/ANTAM_FPS5_27.pdf]

5\_28

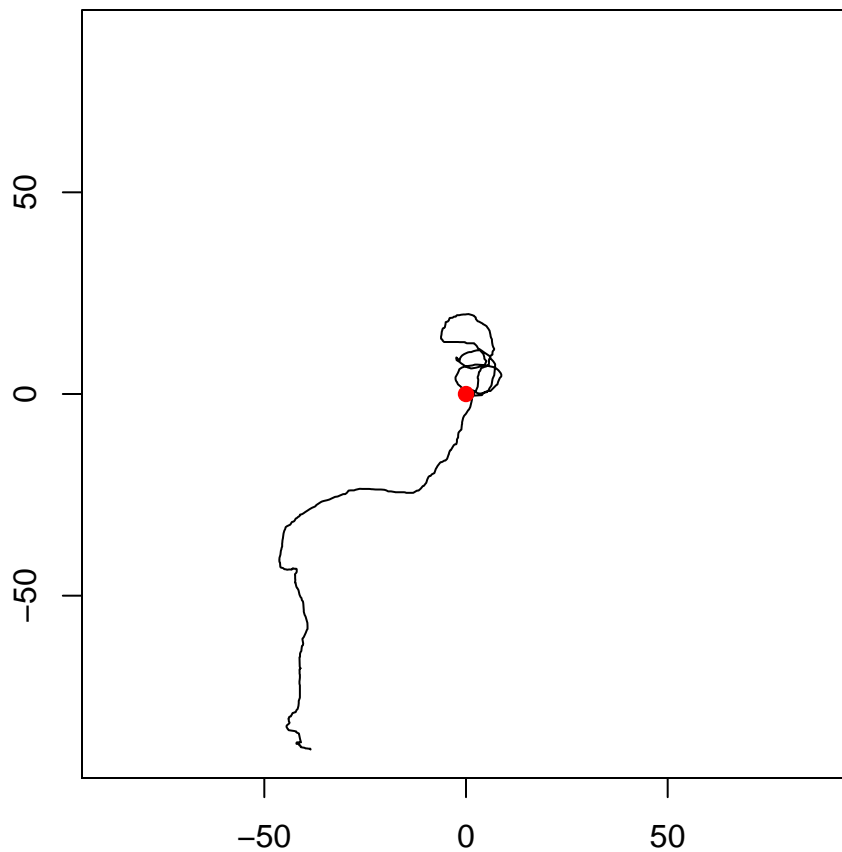

Supplement: S1 Datasets — (ZIP) [file pone.0177480.s006.zip › ANTAM data/trajectories/ANTAM (5FPS)/ANTAM_FPS5_28.pdf]

5\_29

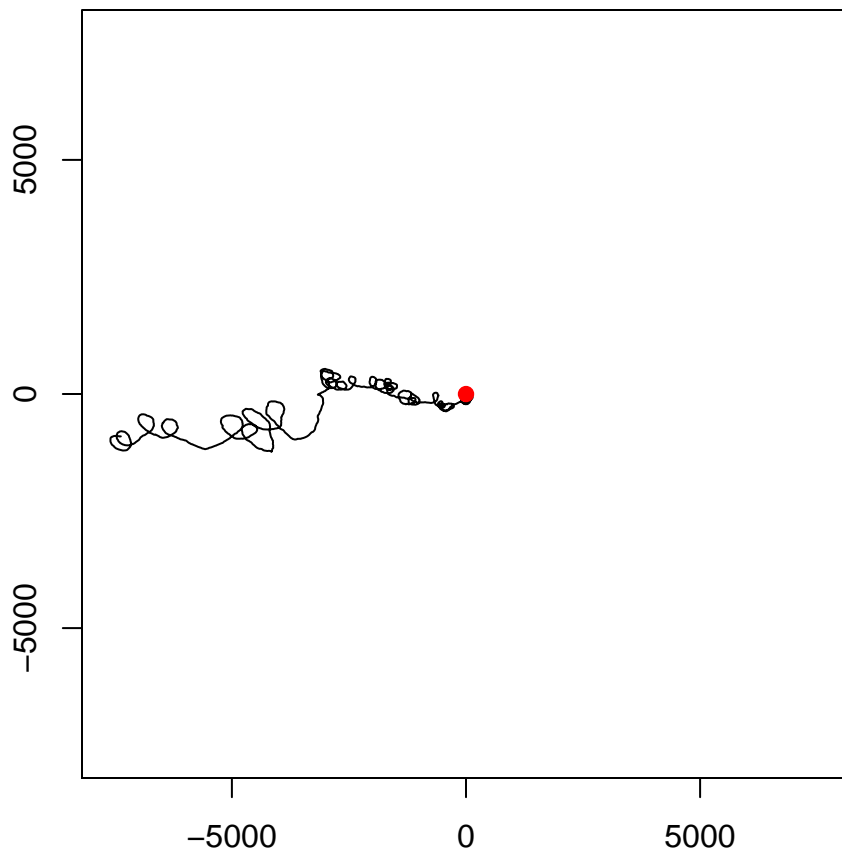

Supplement: S1 Datasets — (ZIP) [file pone.0177480.s006.zip › ANTAM data/trajectories/ANTAM (5FPS)/ANTAM_FPS5_29.pdf]

1

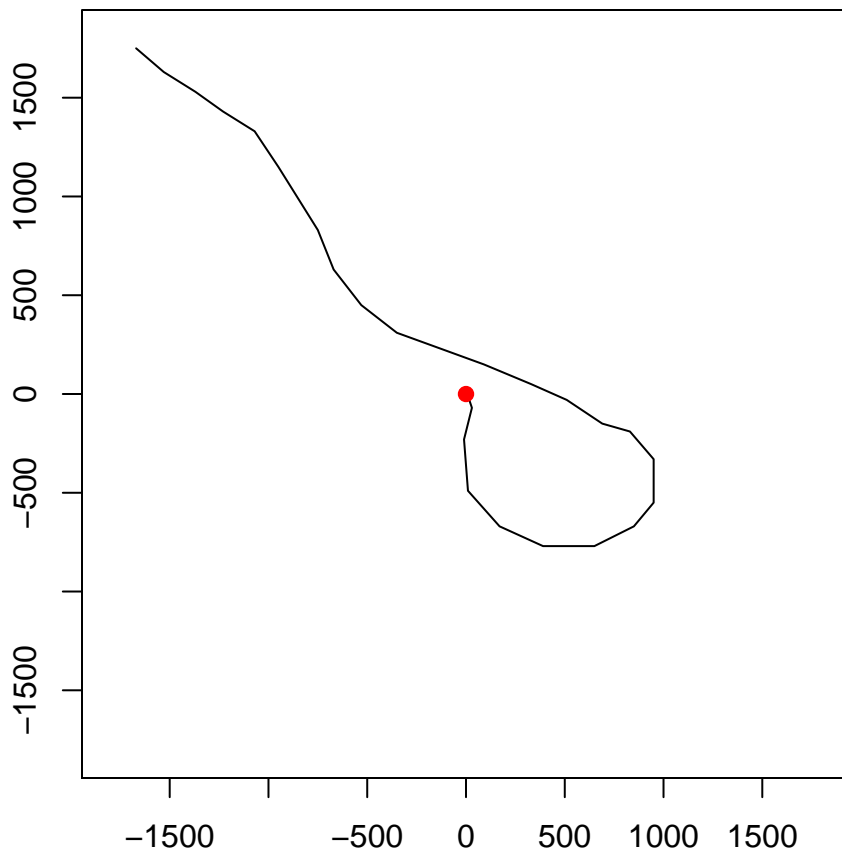

Supplement: S1 Datasets — (ZIP) [file pone.0177480.s006.zip › ANTAM data/trajectories/Large arena(0.1FPS)/poster_FPS0.1_1.pdf]

10

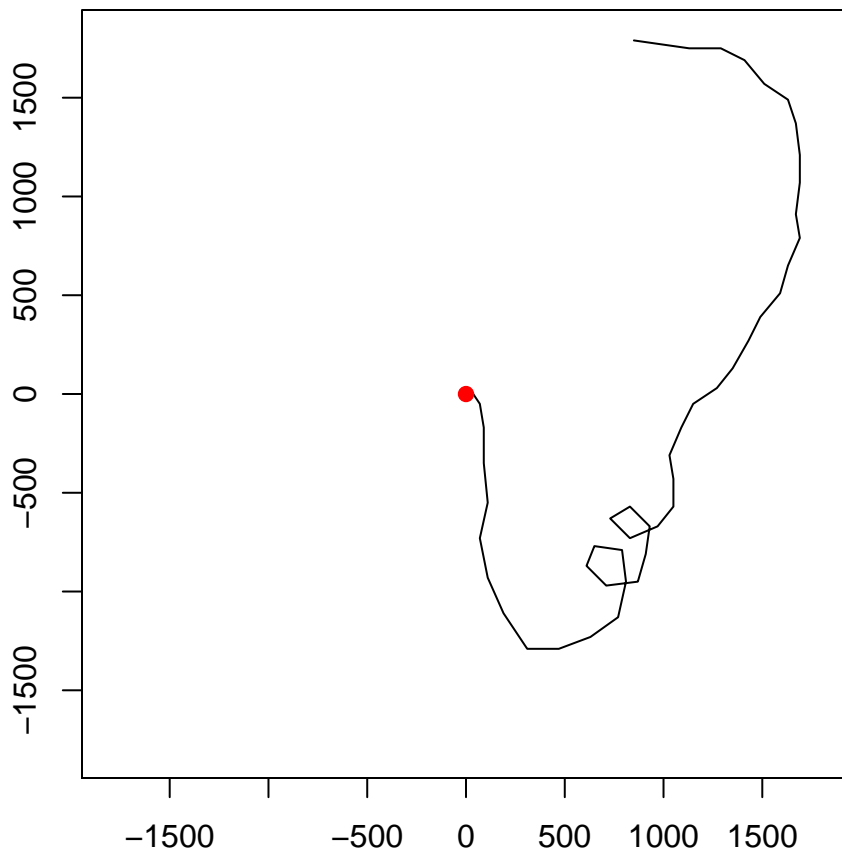

Supplement: S1 Datasets — (ZIP) [file pone.0177480.s006.zip › ANTAM data/trajectories/Large arena(0.1FPS)/poster_FPS0.1_10.pdf]

11

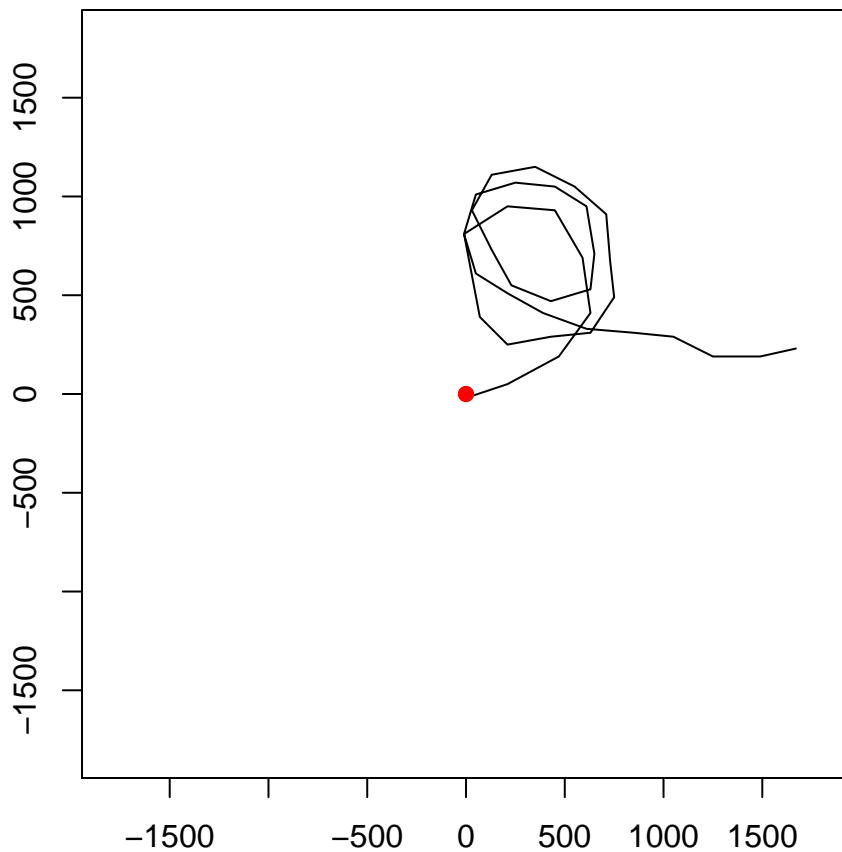

Supplement: S1 Datasets — (ZIP) [file pone.0177480.s006.zip › ANTAM data/trajectories/Large arena(0.1FPS)/poster_FPS0.1_11.pdf]

12

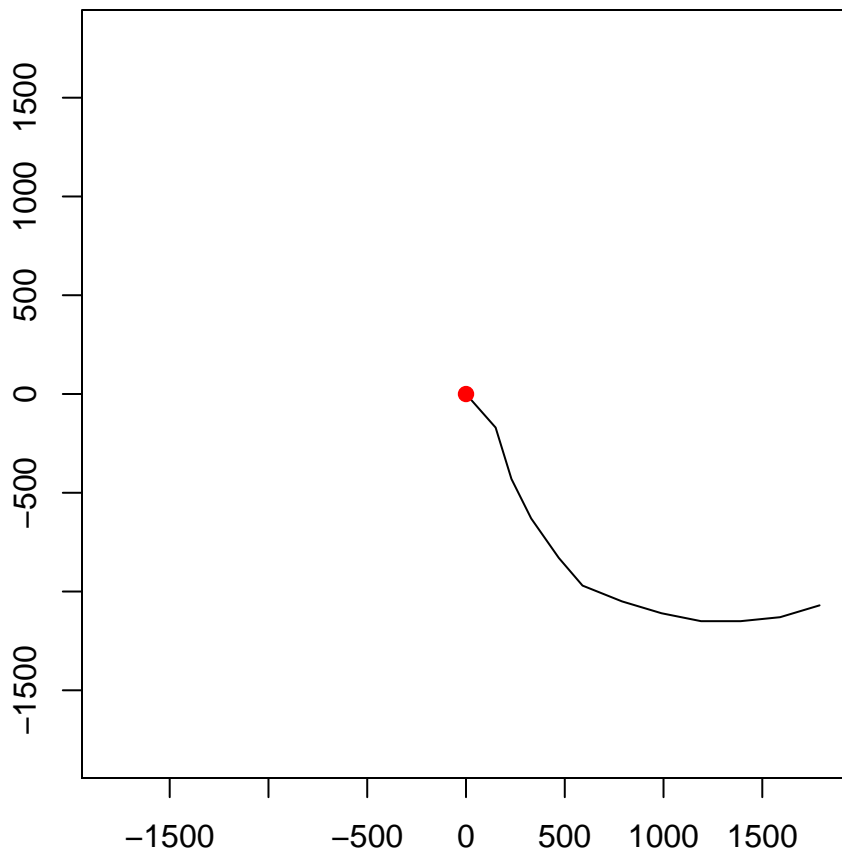

Supplement: S1 Datasets — (ZIP) [file pone.0177480.s006.zip › ANTAM data/trajectories/Large arena(0.1FPS)/poster_FPS0.1_12.pdf]

13

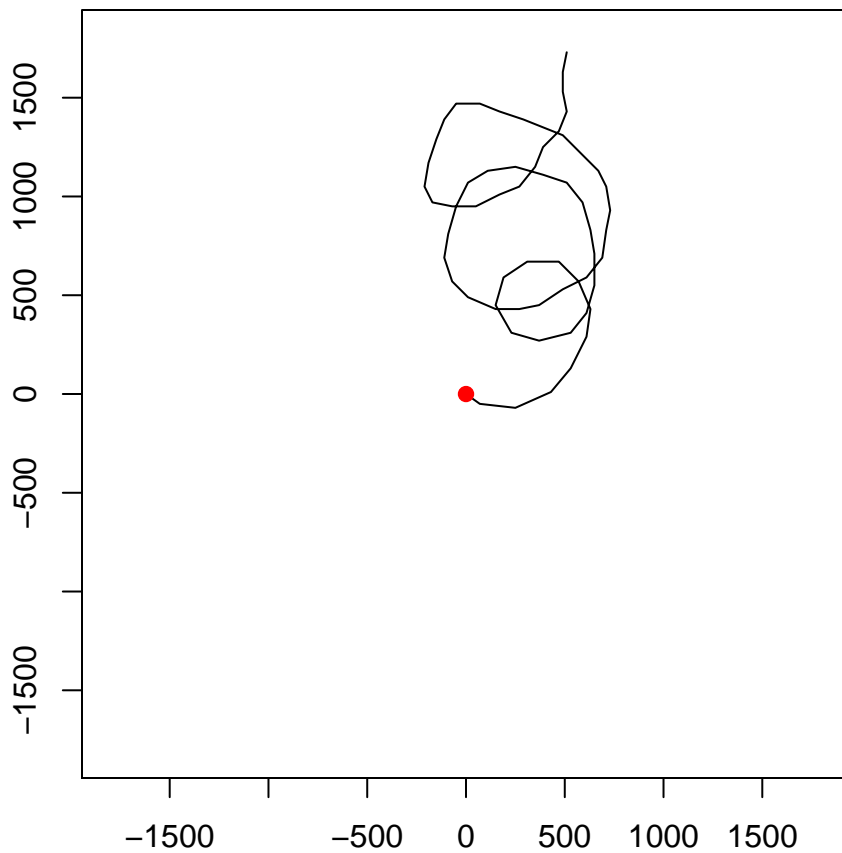

Supplement: S1 Datasets — (ZIP) [file pone.0177480.s006.zip › ANTAM data/trajectories/Large arena(0.1FPS)/poster_FPS0.1_13.pdf]

14

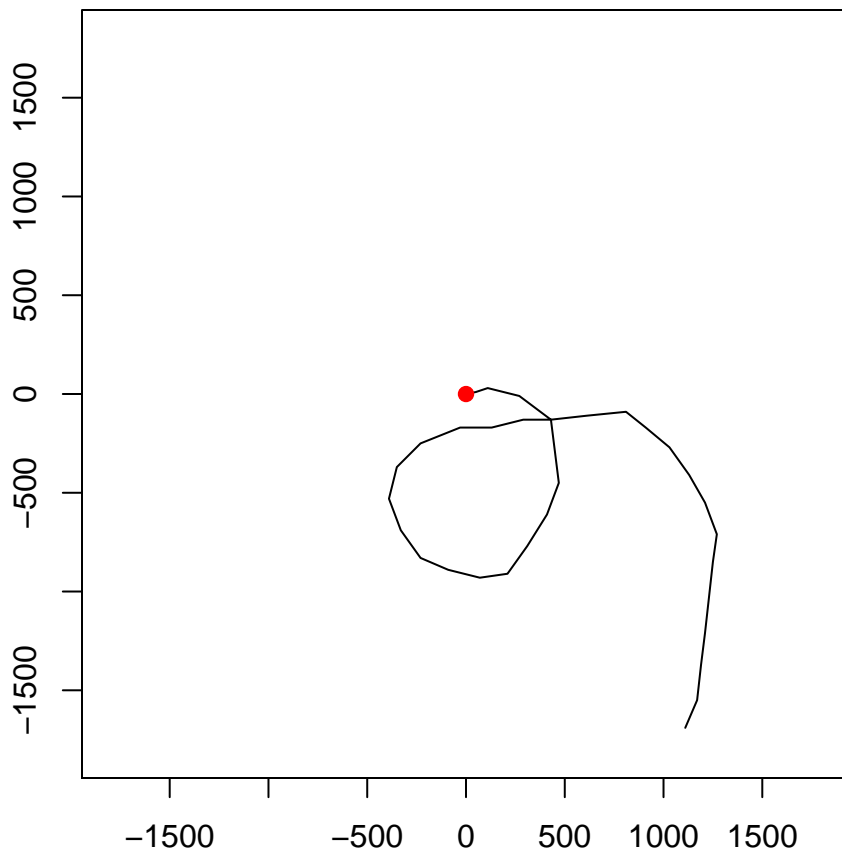

Supplement: S1 Datasets — (ZIP) [file pone.0177480.s006.zip › ANTAM data/trajectories/Large arena(0.1FPS)/poster_FPS0.1_14.pdf]

15

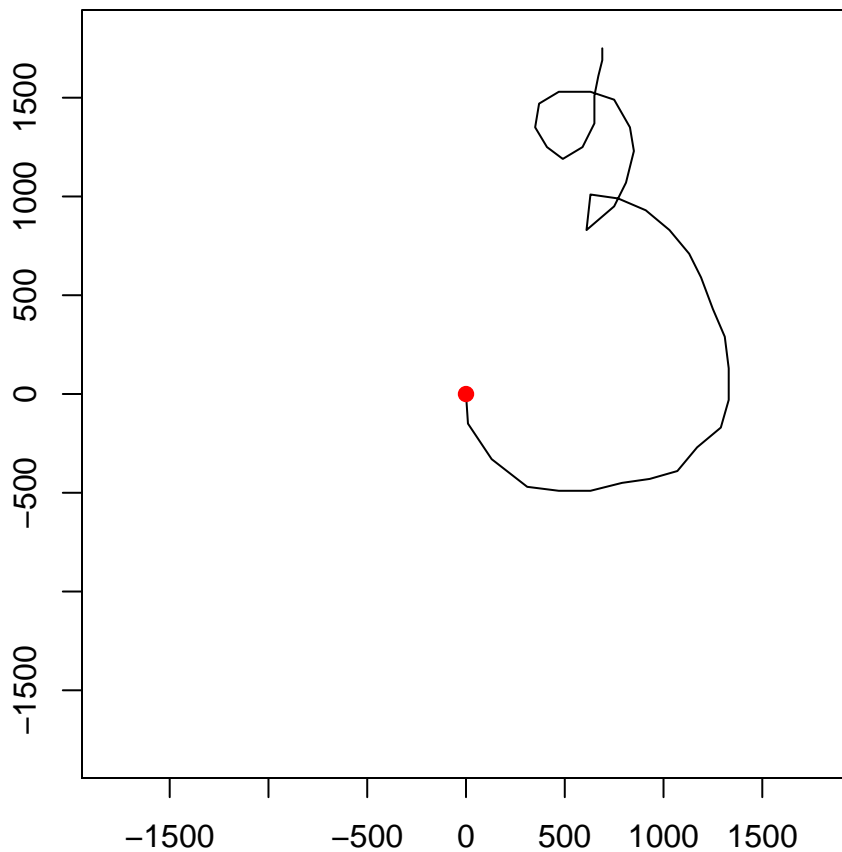

Supplement: S1 Datasets — (ZIP) [file pone.0177480.s006.zip › ANTAM data/trajectories/Large arena(0.1FPS)/poster_FPS0.1_15.pdf]

16

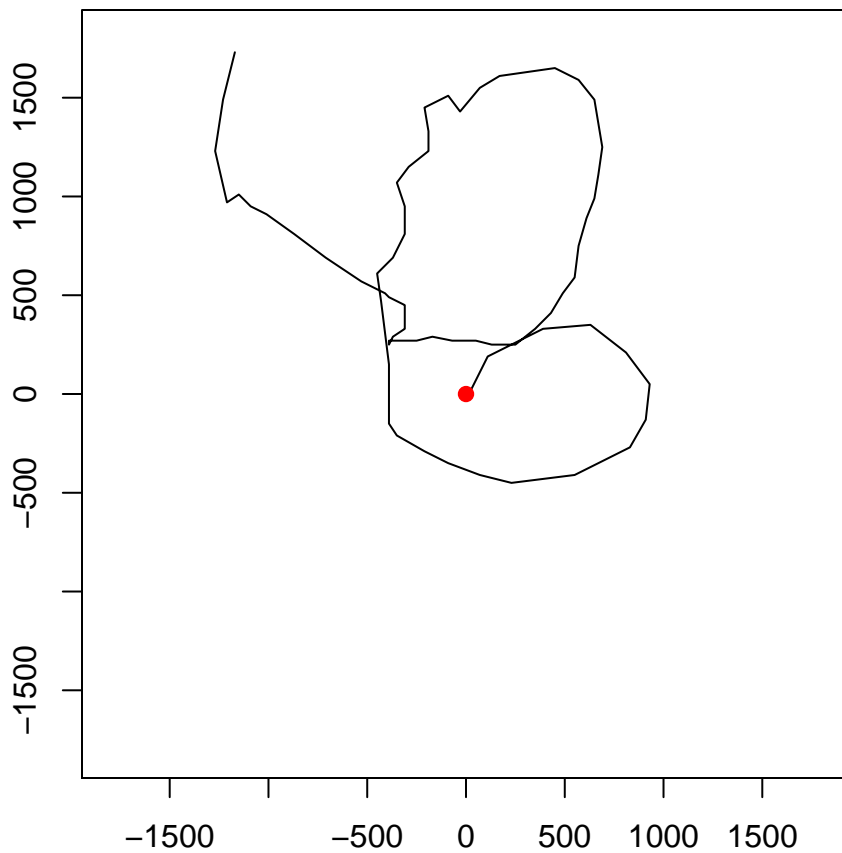

Supplement: S1 Datasets — (ZIP) [file pone.0177480.s006.zip › ANTAM data/trajectories/Large arena(0.1FPS)/poster_FPS0.1_16.pdf]

17

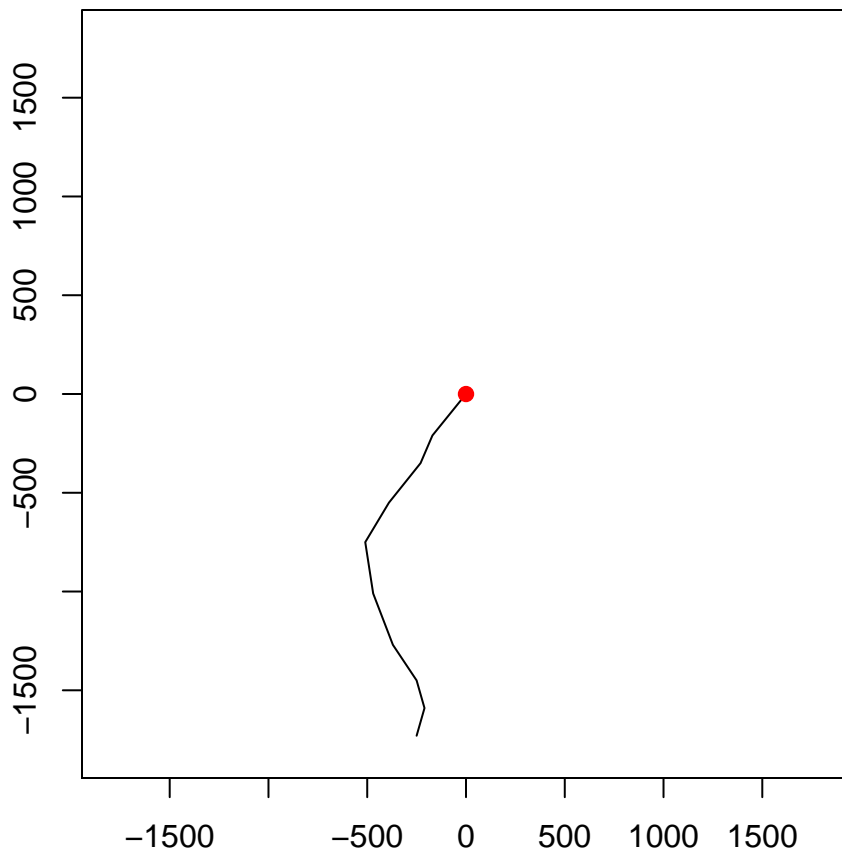

Supplement: S1 Datasets — (ZIP) [file pone.0177480.s006.zip › ANTAM data/trajectories/Large arena(0.1FPS)/poster_FPS0.1_17.pdf]

18

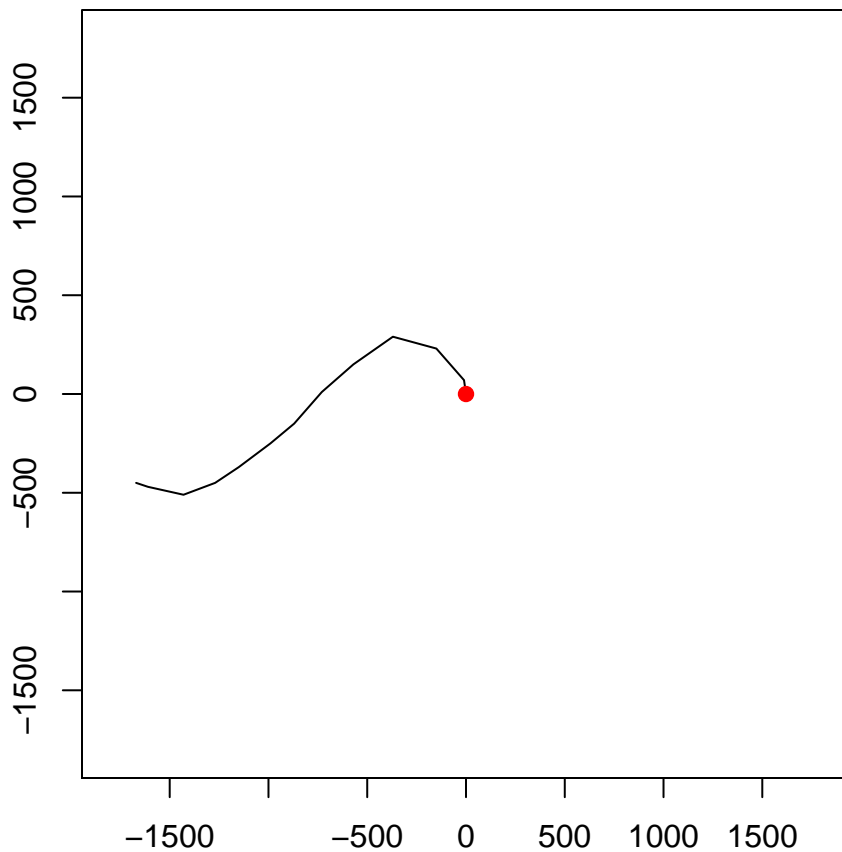

Supplement: S1 Datasets — (ZIP) [file pone.0177480.s006.zip › ANTAM data/trajectories/Large arena(0.1FPS)/poster_FPS0.1_18.pdf]

19

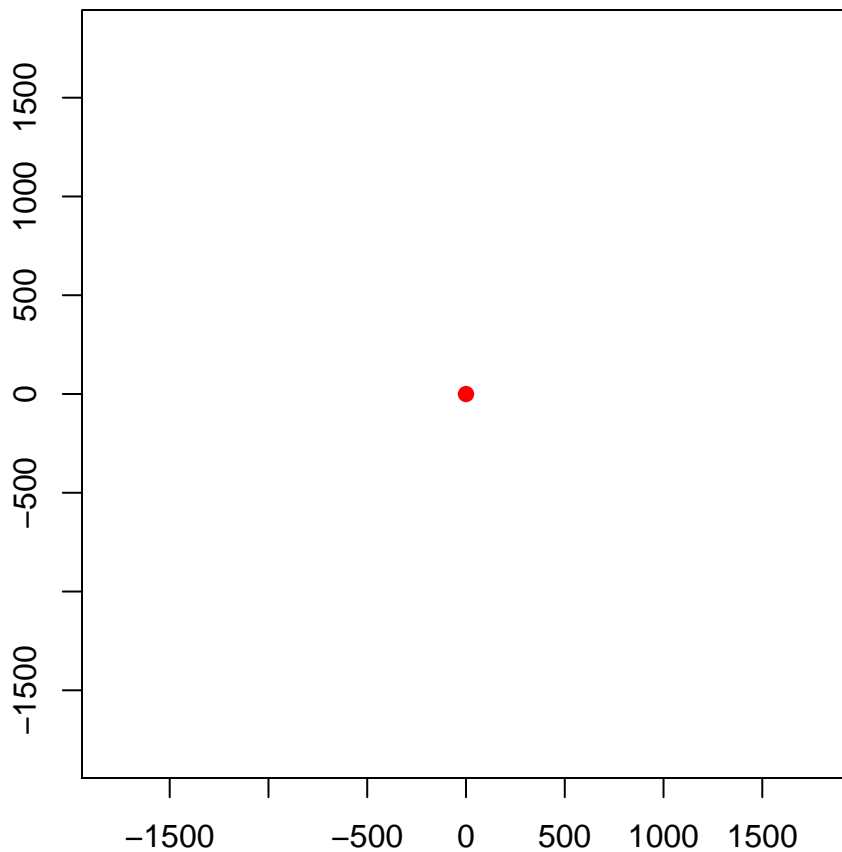

Supplement: S1 Datasets — (ZIP) [file pone.0177480.s006.zip › ANTAM data/trajectories/Large arena(0.1FPS)/poster_FPS0.1_19.pdf]

2

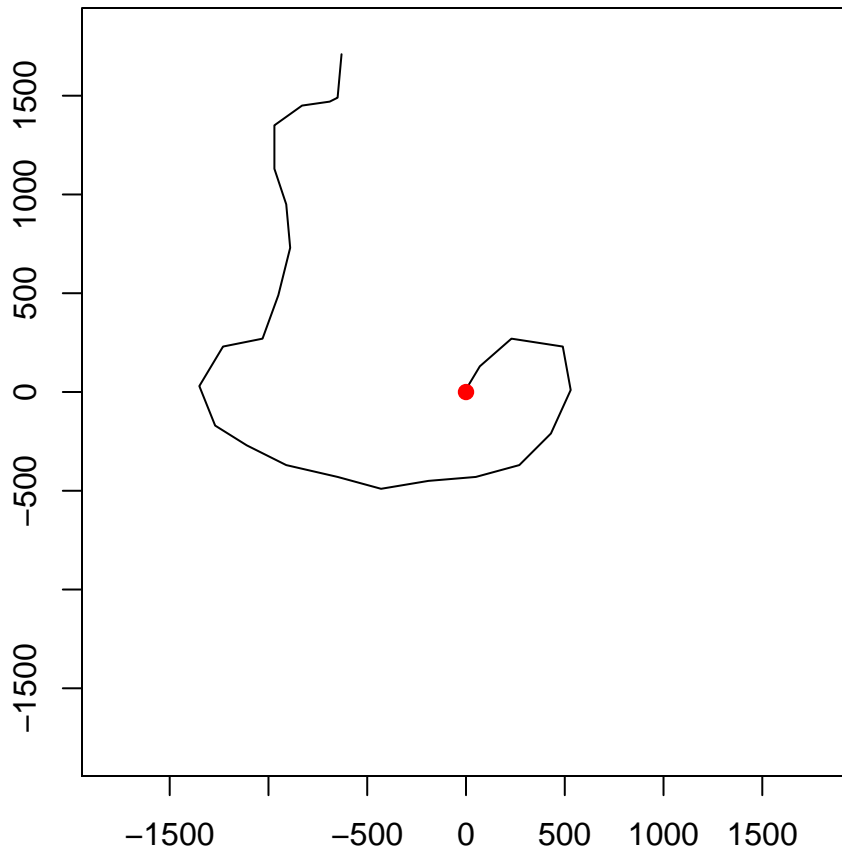

Supplement: S1 Datasets — (ZIP) [file pone.0177480.s006.zip › ANTAM data/trajectories/Large arena(0.1FPS)/poster_FPS0.1_2.pdf]

20

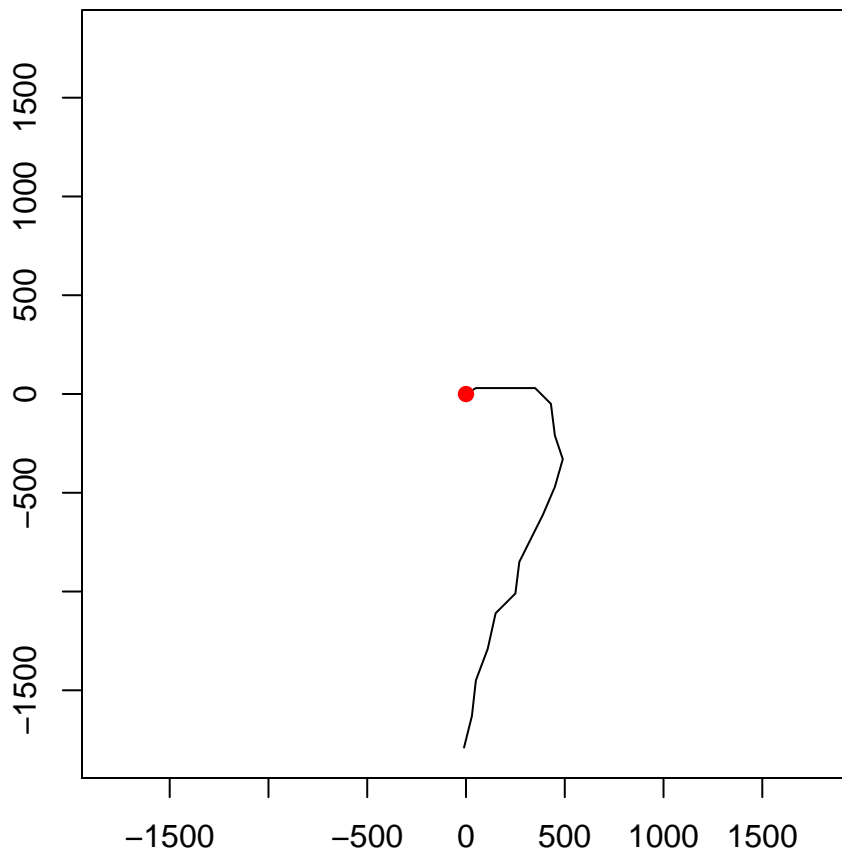

Supplement: S1 Datasets — (ZIP) [file pone.0177480.s006.zip › ANTAM data/trajectories/Large arena(0.1FPS)/poster_FPS0.1_20.pdf]

21

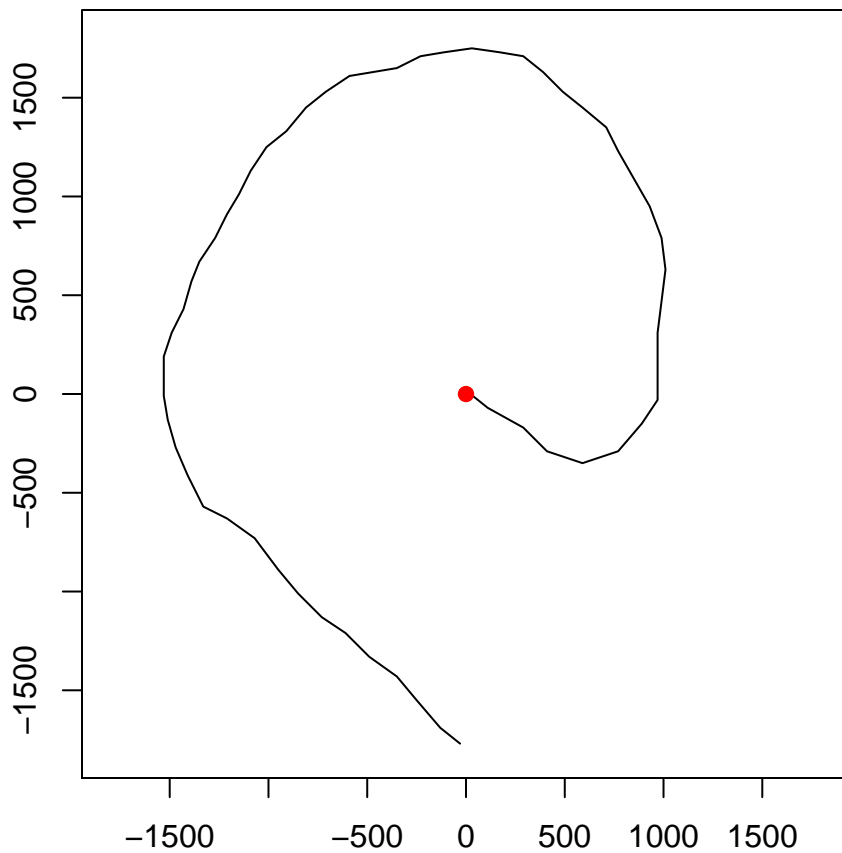

Supplement: S1 Datasets — (ZIP) [file pone.0177480.s006.zip › ANTAM data/trajectories/Large arena(0.1FPS)/poster_FPS0.1_21.pdf]

22

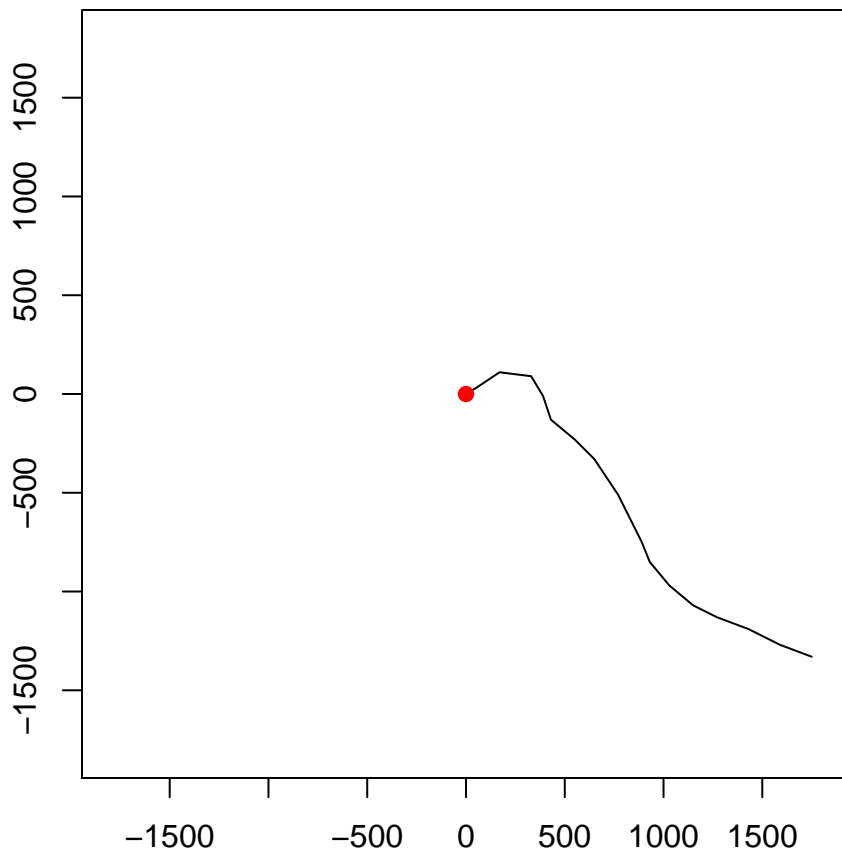

Supplement: S1 Datasets — (ZIP) [file pone.0177480.s006.zip › ANTAM data/trajectories/Large arena(0.1FPS)/poster_FPS0.1_22.pdf]

23

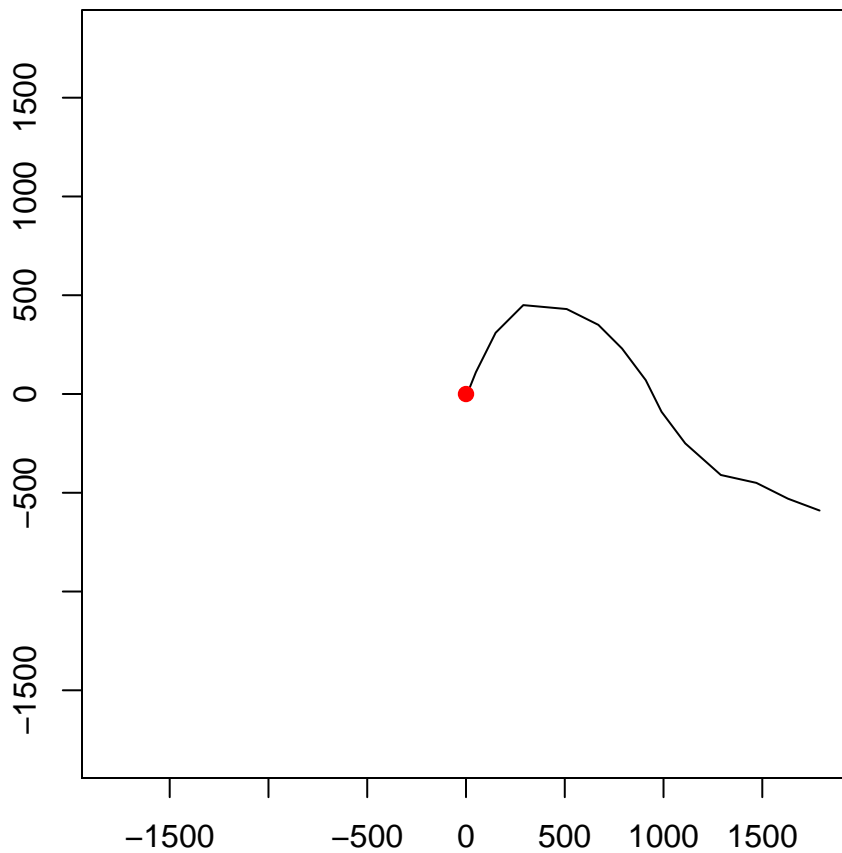

Supplement: S1 Datasets — (ZIP) [file pone.0177480.s006.zip › ANTAM data/trajectories/Large arena(0.1FPS)/poster_FPS0.1_23.pdf]

24

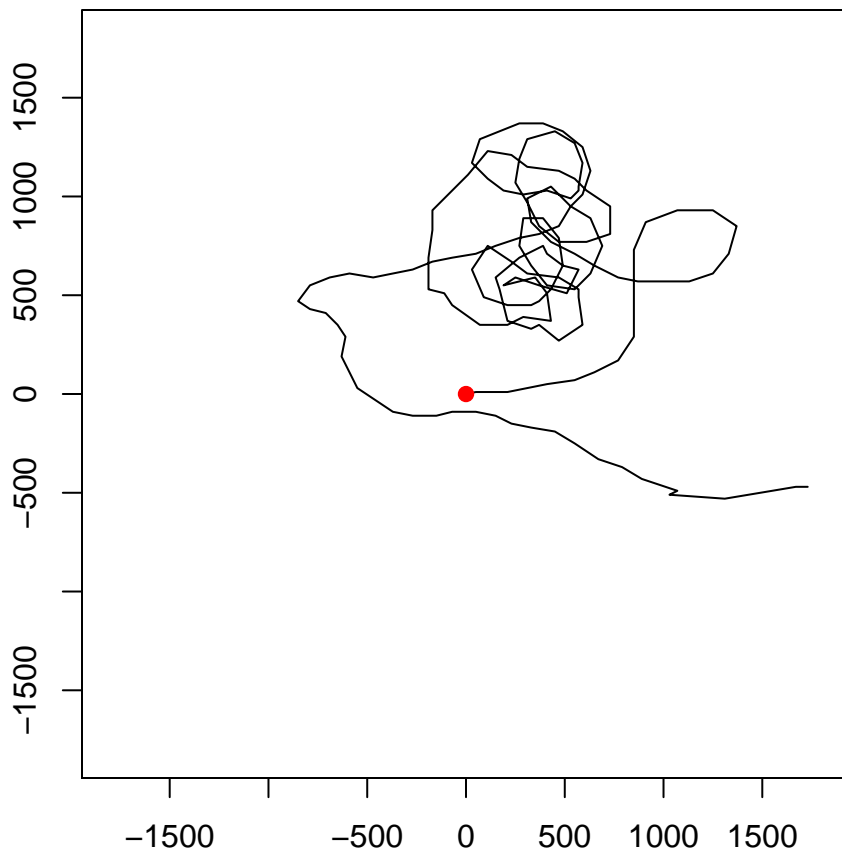

Supplement: S1 Datasets — (ZIP) [file pone.0177480.s006.zip › ANTAM data/trajectories/Large arena(0.1FPS)/poster_FPS0.1_24.pdf]

25

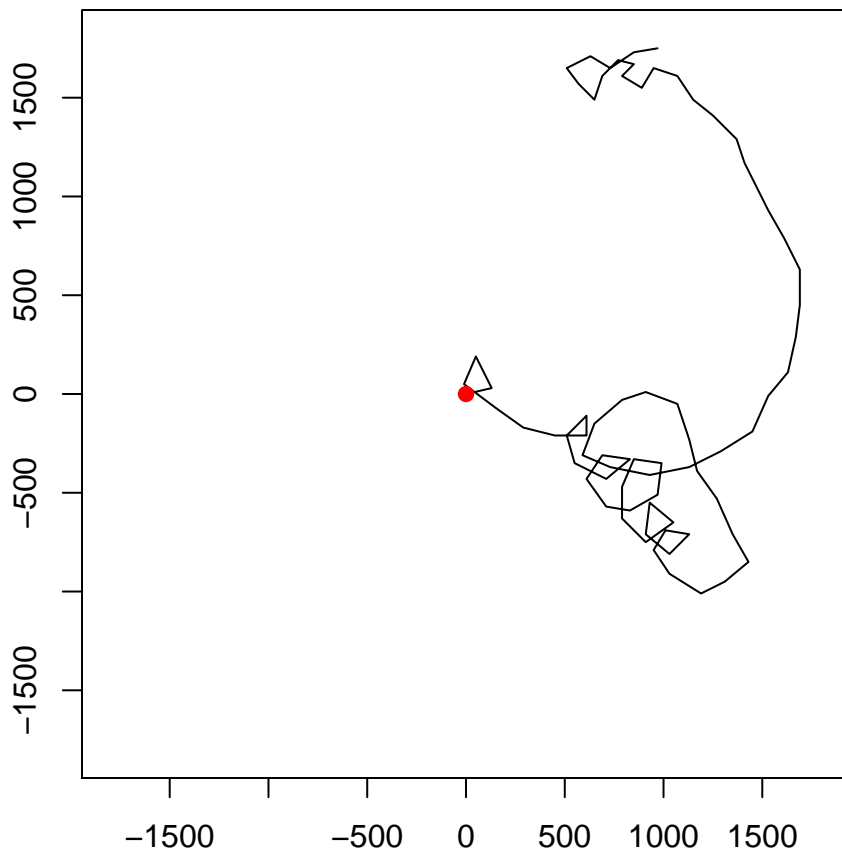

Supplement: S1 Datasets — (ZIP) [file pone.0177480.s006.zip › ANTAM data/trajectories/Large arena(0.1FPS)/poster_FPS0.1_25.pdf]

26

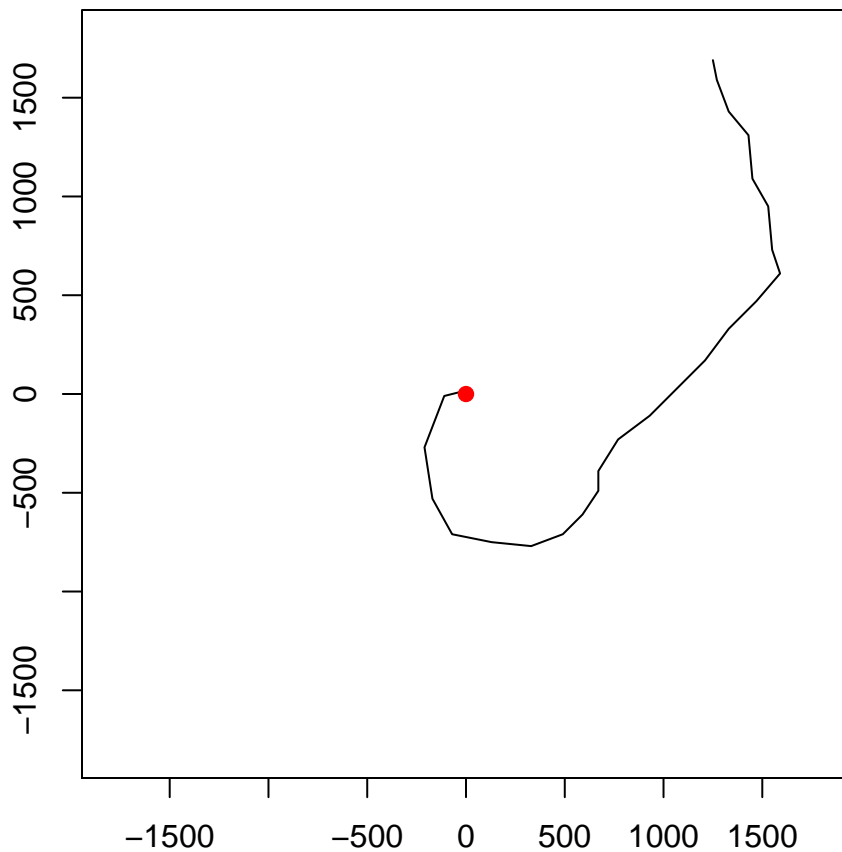

Supplement: S1 Datasets — (ZIP) [file pone.0177480.s006.zip › ANTAM data/trajectories/Large arena(0.1FPS)/poster_FPS0.1_26.pdf]

**27**

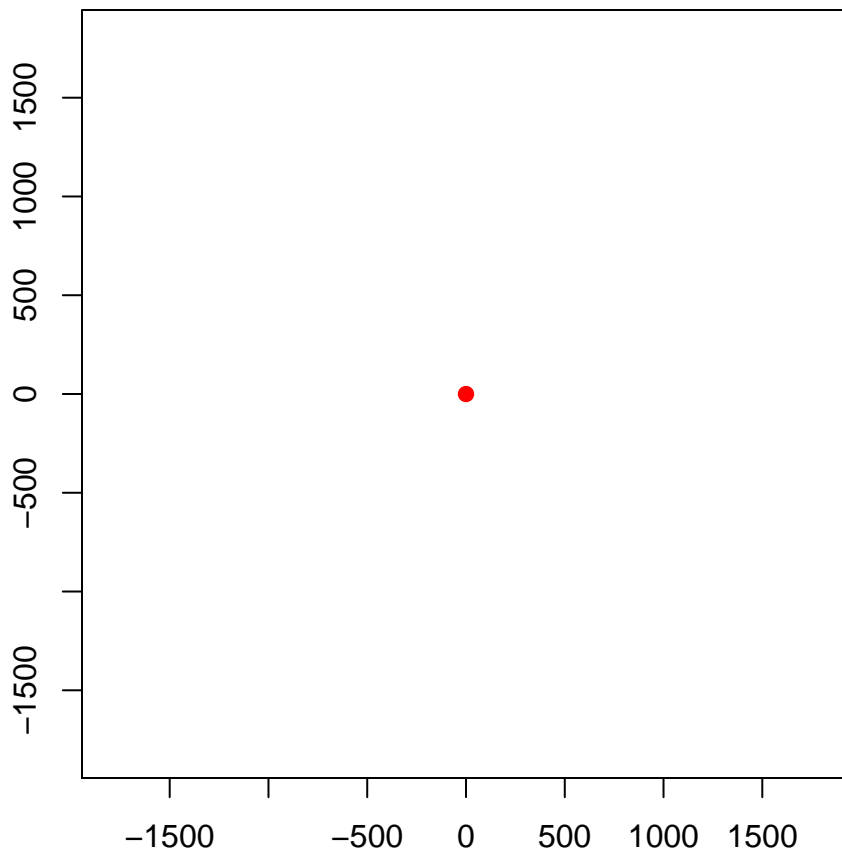

Supplement: S1 Datasets — (ZIP) [file pone.0177480.s006.zip › ANTAM data/trajectories/Large arena(0.1FPS)/poster_FPS0.1_27.pdf]

28

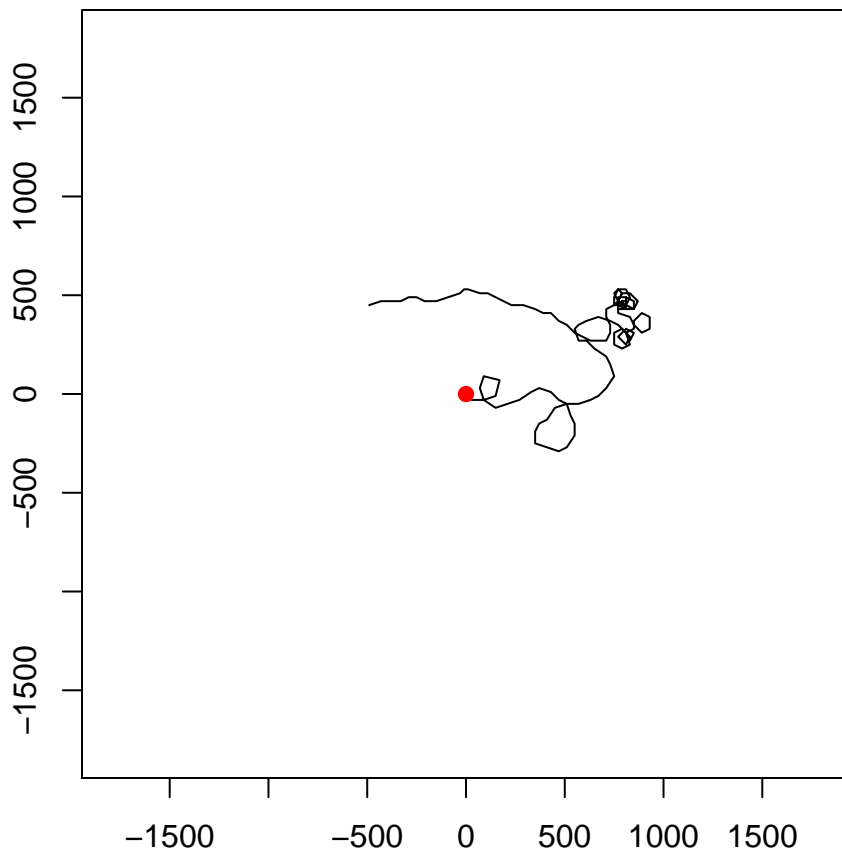

Supplement: S1 Datasets — (ZIP) [file pone.0177480.s006.zip › ANTAM data/trajectories/Large arena(0.1FPS)/poster_FPS0.1_28.pdf]

29

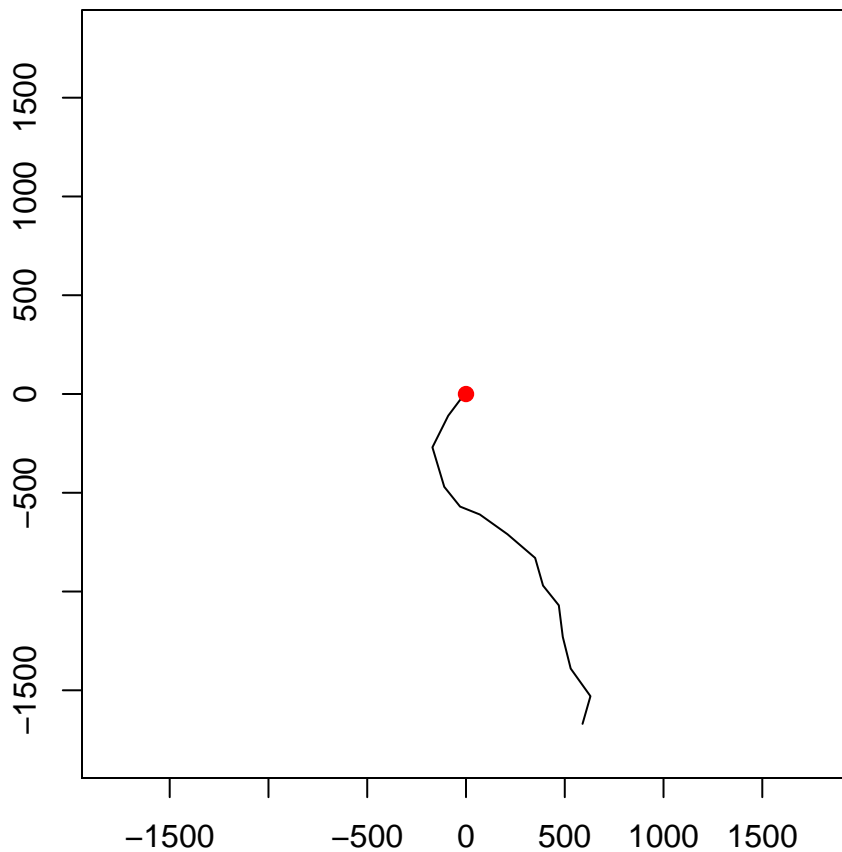

Supplement: S1 Datasets — (ZIP) [file pone.0177480.s006.zip › ANTAM data/trajectories/Large arena(0.1FPS)/poster_FPS0.1_29.pdf]

3

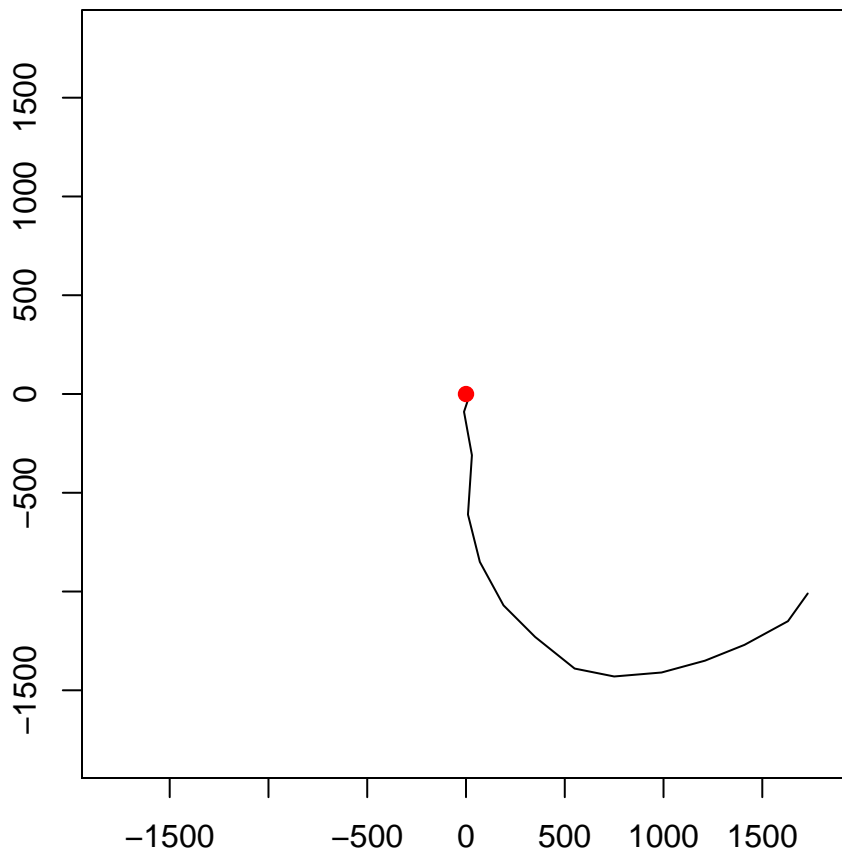

Supplement: S1 Datasets — (ZIP) [file pone.0177480.s006.zip › ANTAM data/trajectories/Large arena(0.1FPS)/poster_FPS0.1_3.pdf]

4

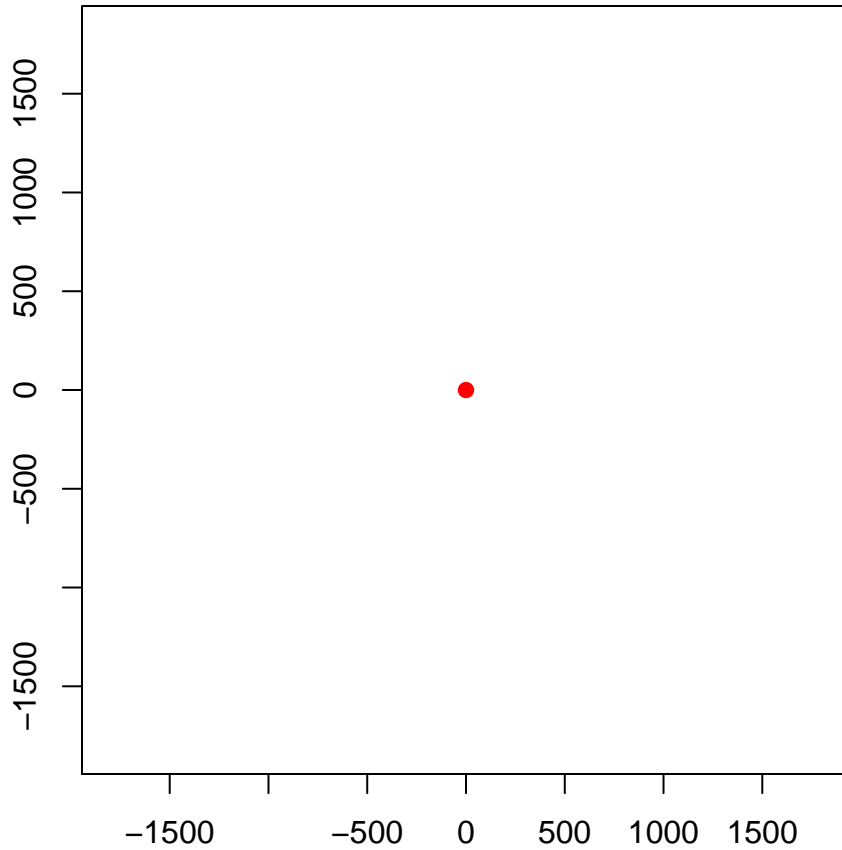

Supplement: S1 Datasets — (ZIP) [file pone.0177480.s006.zip › ANTAM data/trajectories/Large arena(0.1FPS)/poster_FPS0.1_4.pdf]

5

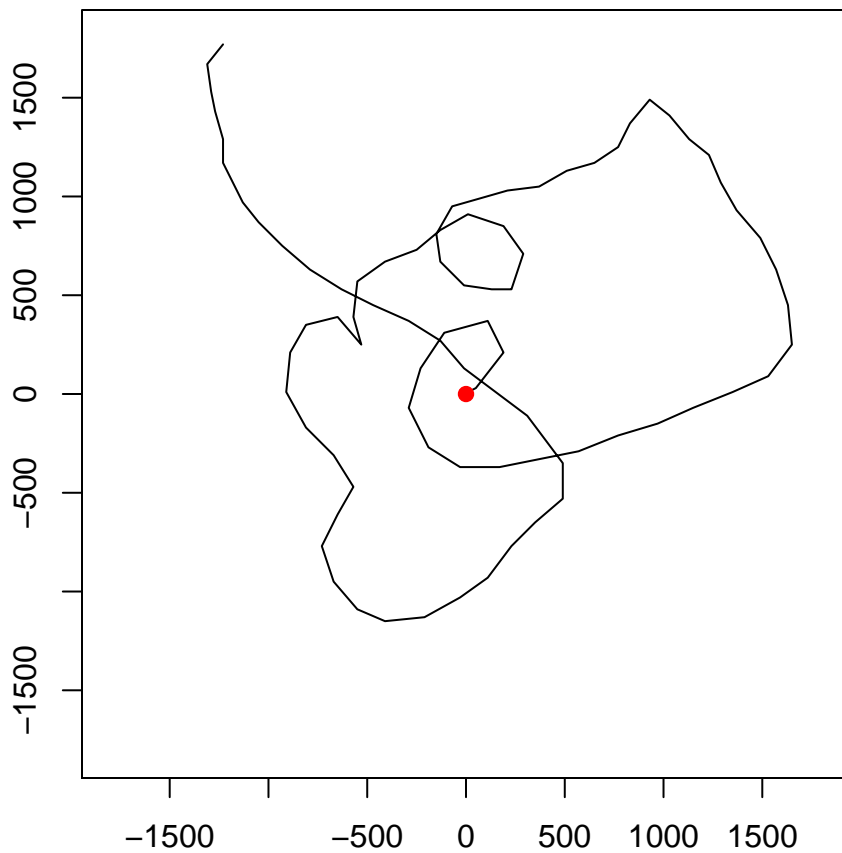

Supplement: S1 Datasets — (ZIP) [file pone.0177480.s006.zip › ANTAM data/trajectories/Large arena(0.1FPS)/poster_FPS0.1_5.pdf]

6

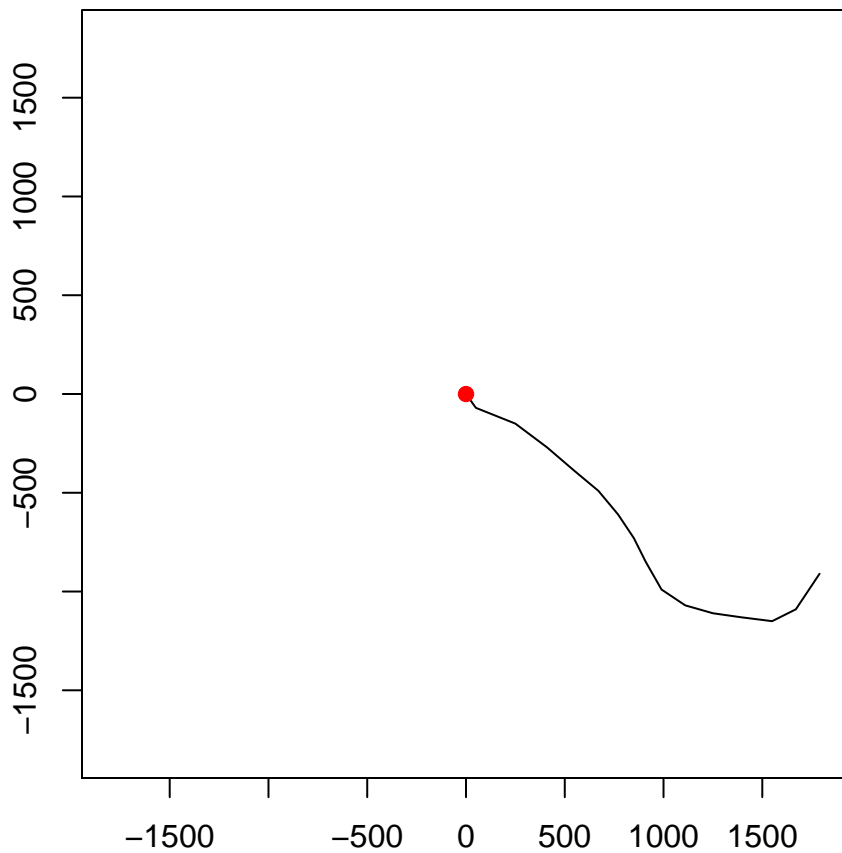

Supplement: S1 Datasets — (ZIP) [file pone.0177480.s006.zip › ANTAM data/trajectories/Large arena(0.1FPS)/poster_FPS0.1_6.pdf]

7

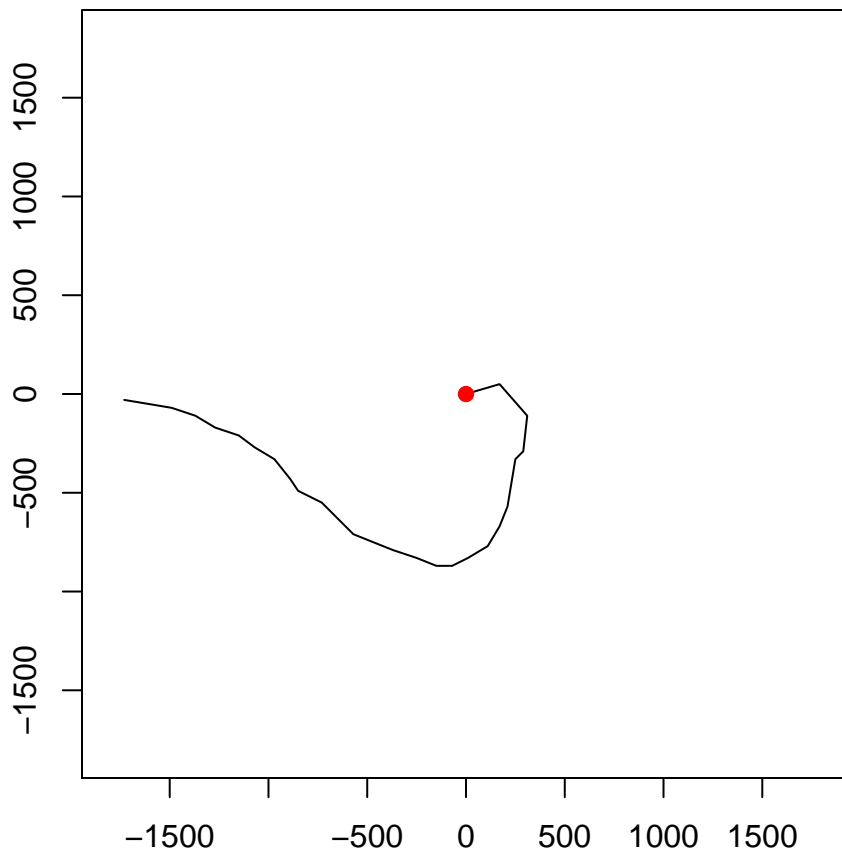

Supplement: S1 Datasets — (ZIP) [file pone.0177480.s006.zip › ANTAM data/trajectories/Large arena(0.1FPS)/poster_FPS0.1_7.pdf]

8

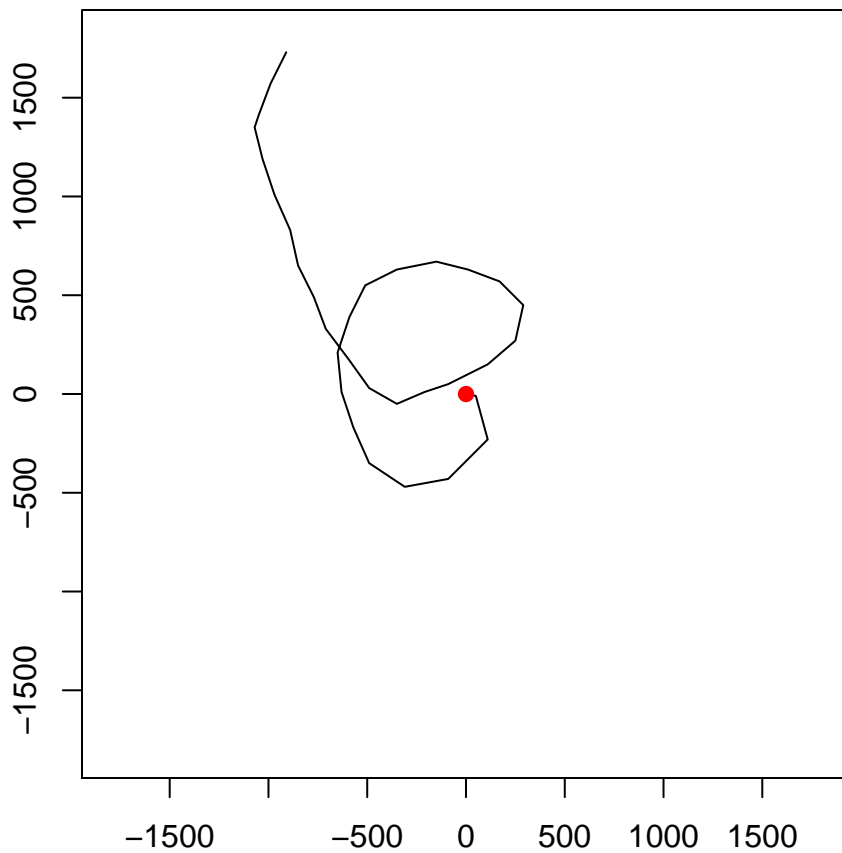

Supplement: S1 Datasets — (ZIP) [file pone.0177480.s006.zip › ANTAM data/trajectories/Large arena(0.1FPS)/poster_FPS0.1_8.pdf]

9

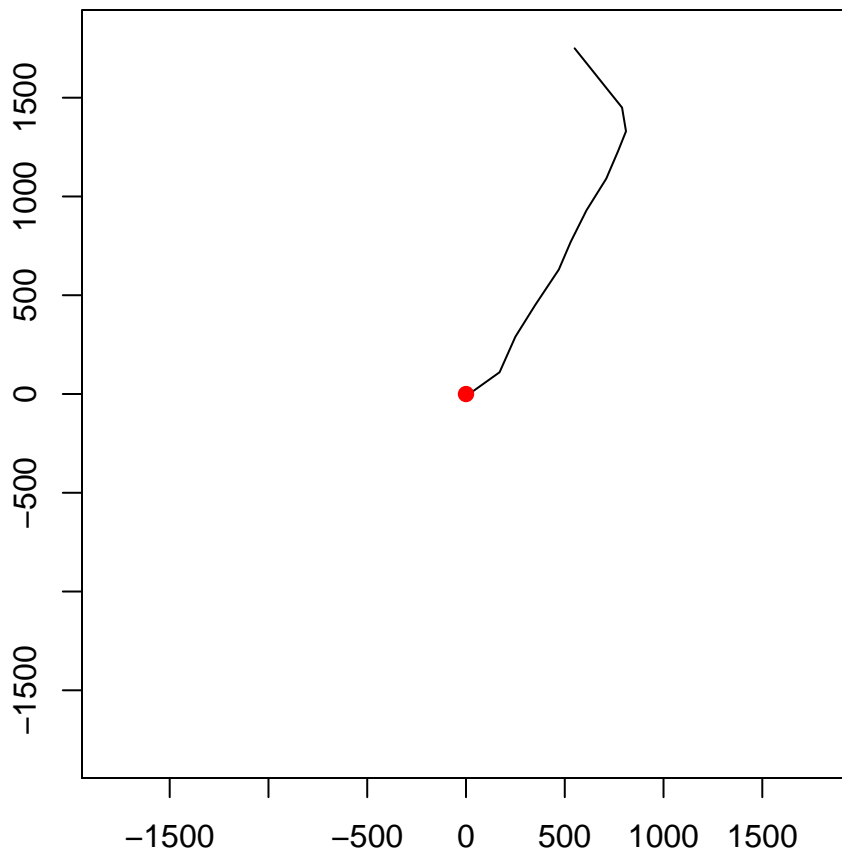

Supplement: S1 Datasets — (ZIP) [file pone.0177480.s006.zip › ANTAM data/trajectories/Large arena(0.1FPS)/poster_FPS0.1_9.pdf]

**d\_01**

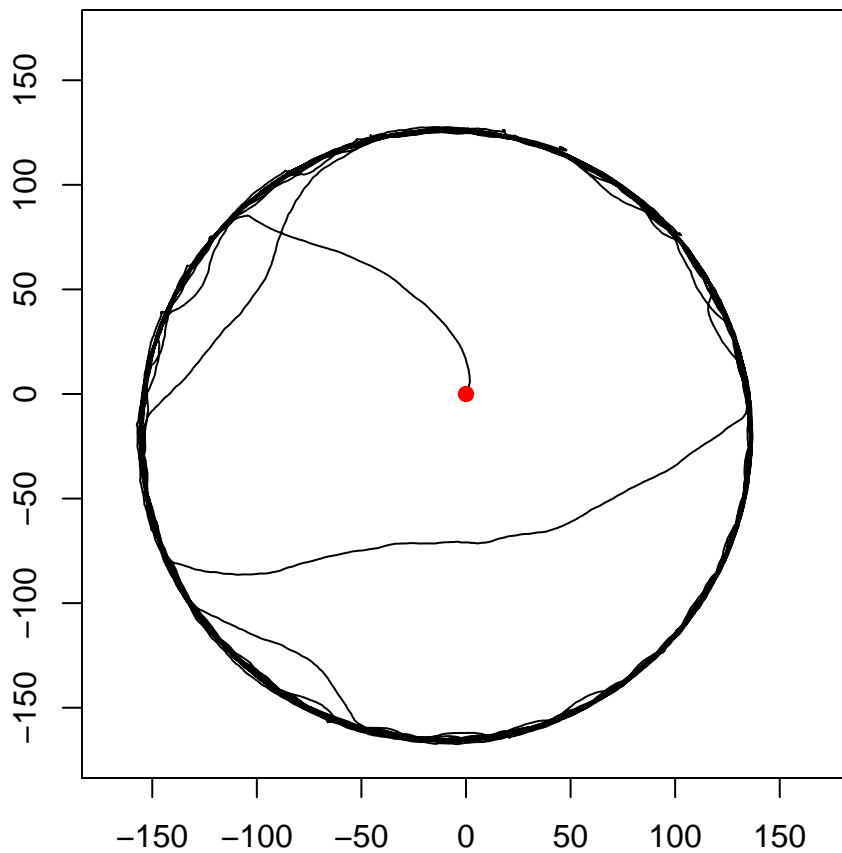

Supplement: S1 Datasets — (ZIP) [file pone.0177480.s006.zip › ANTAM data/trajectories/Small arena (5FPS)/d_01.pdf]

d\_02

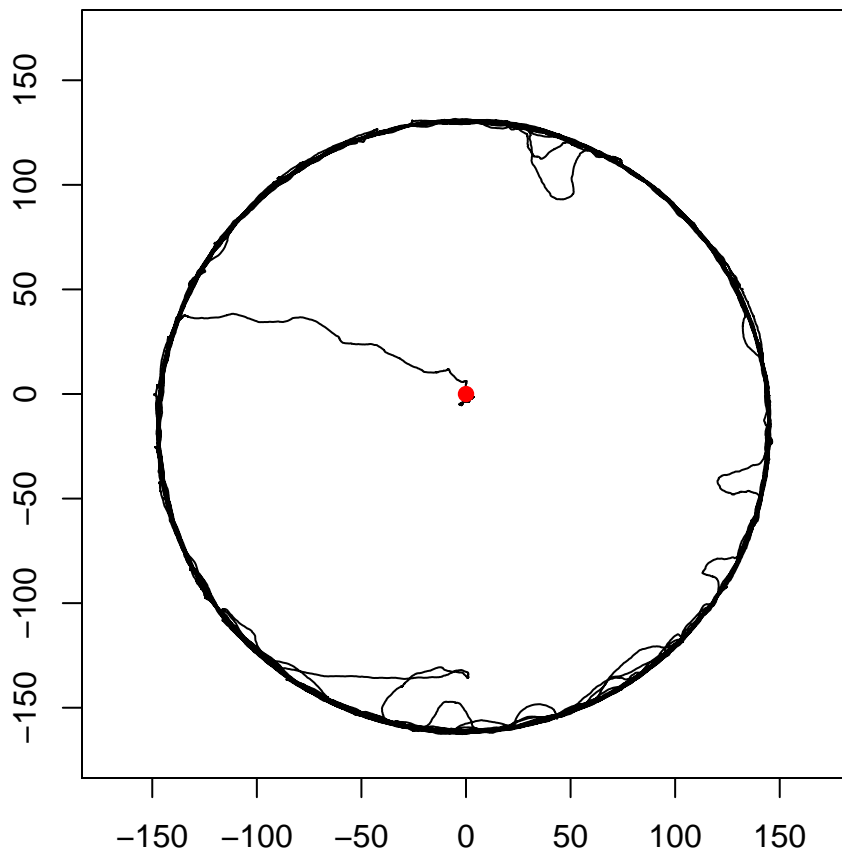

Supplement: S1 Datasets — (ZIP) [file pone.0177480.s006.zip › ANTAM data/trajectories/Small arena (5FPS)/d_02.pdf]

**d\_03**

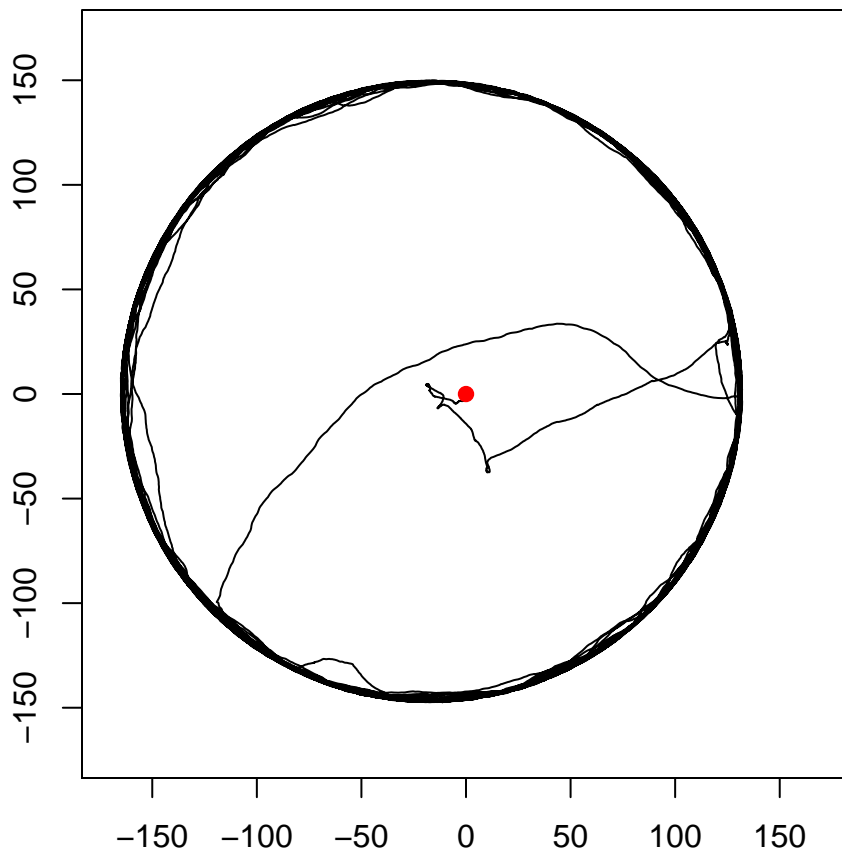

Supplement: S1 Datasets — (ZIP) [file pone.0177480.s006.zip › ANTAM data/trajectories/Small arena (5FPS)/d_03.pdf]

**d\_04**

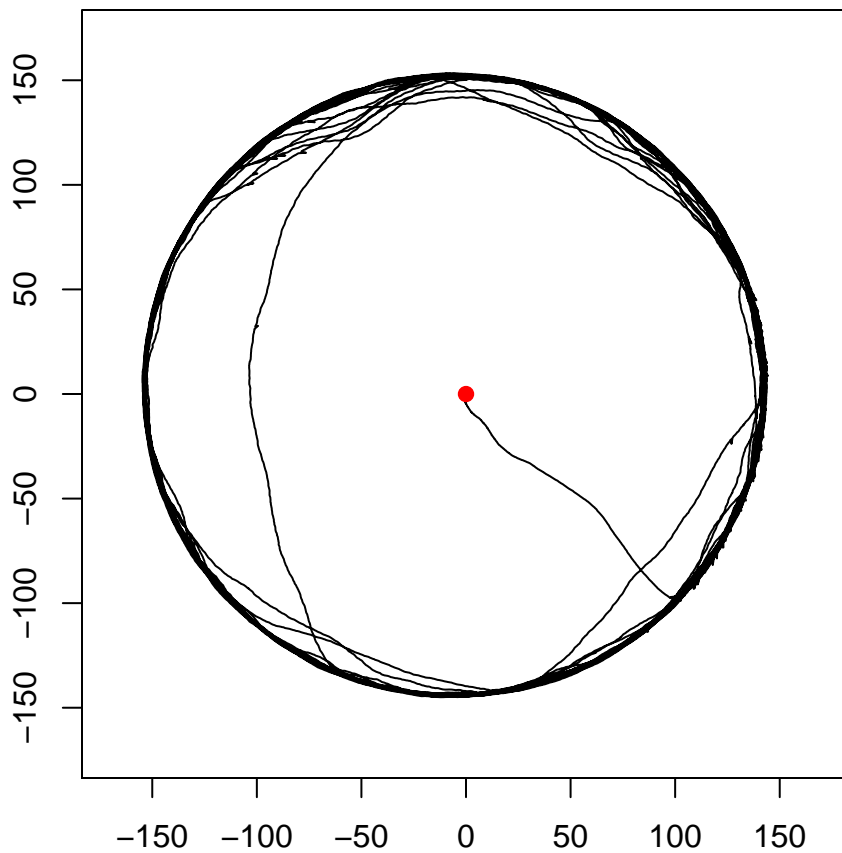

Supplement: S1 Datasets — (ZIP) [file pone.0177480.s006.zip › ANTAM data/trajectories/Small arena (5FPS)/d_04.pdf]

d\_05

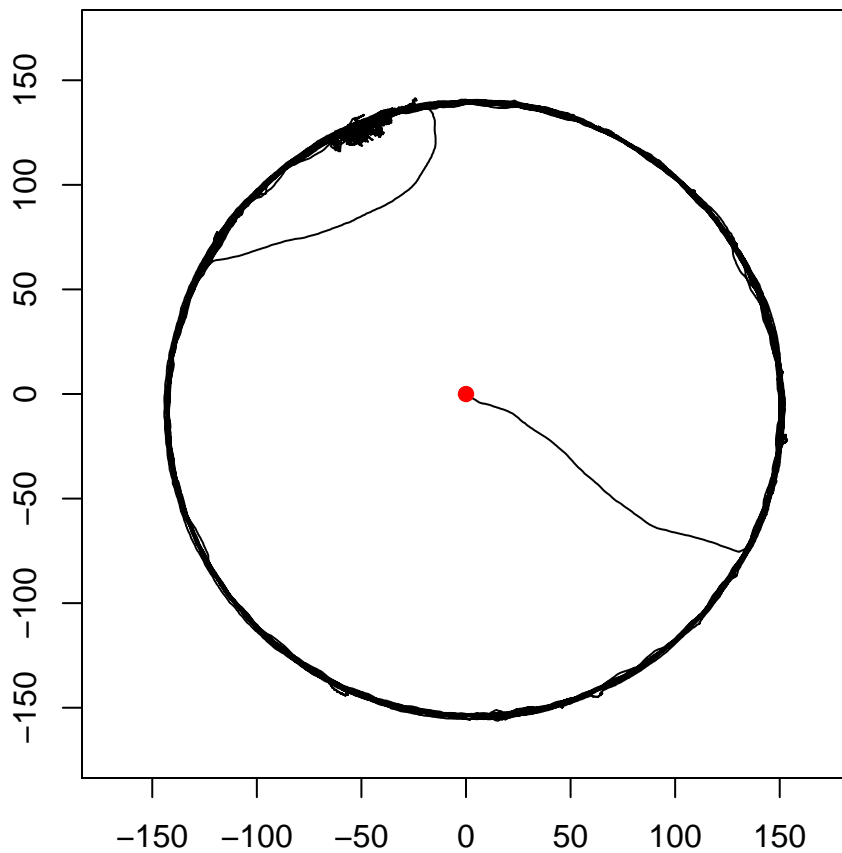

Supplement: S1 Datasets — (ZIP) [file pone.0177480.s006.zip › ANTAM data/trajectories/Small arena (5FPS)/d_05.pdf]

**d\_06**

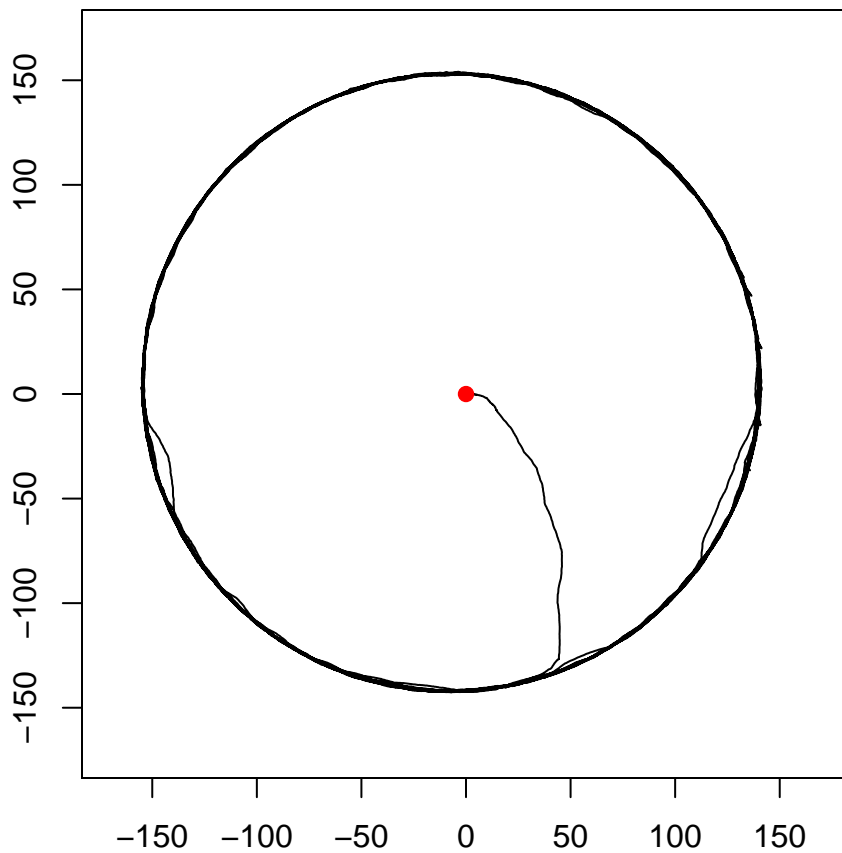

Supplement: S1 Datasets — (ZIP) [file pone.0177480.s006.zip › ANTAM data/trajectories/Small arena (5FPS)/d_06.pdf]

**d\_07**

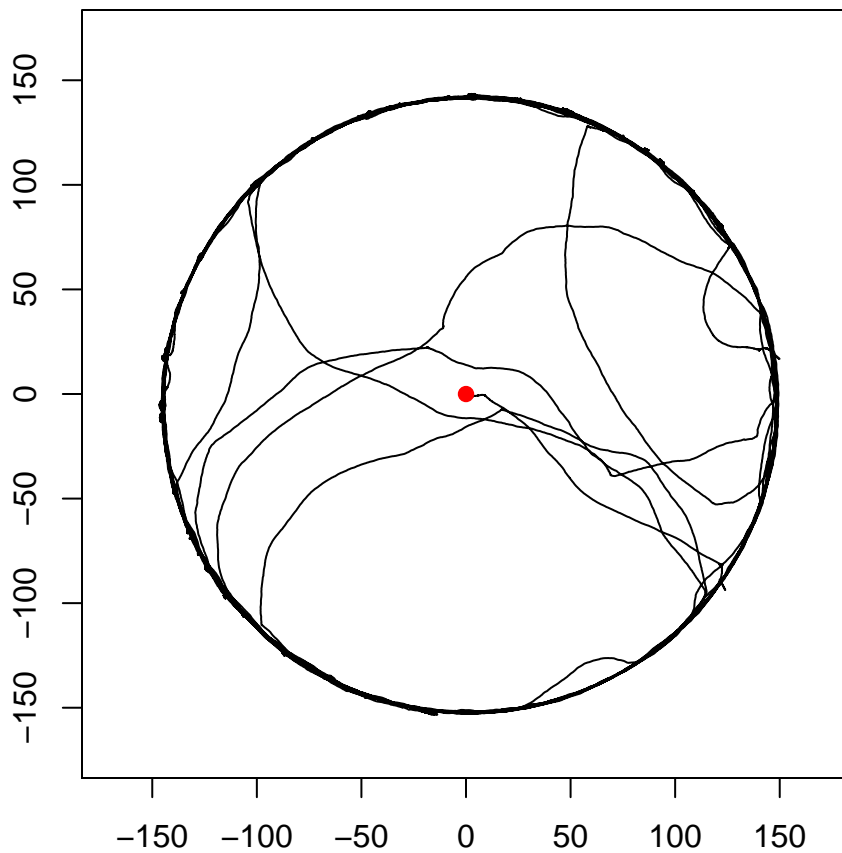

Supplement: S1 Datasets — (ZIP) [file pone.0177480.s006.zip › ANTAM data/trajectories/Small arena (5FPS)/d_07.pdf]

**d\_08**

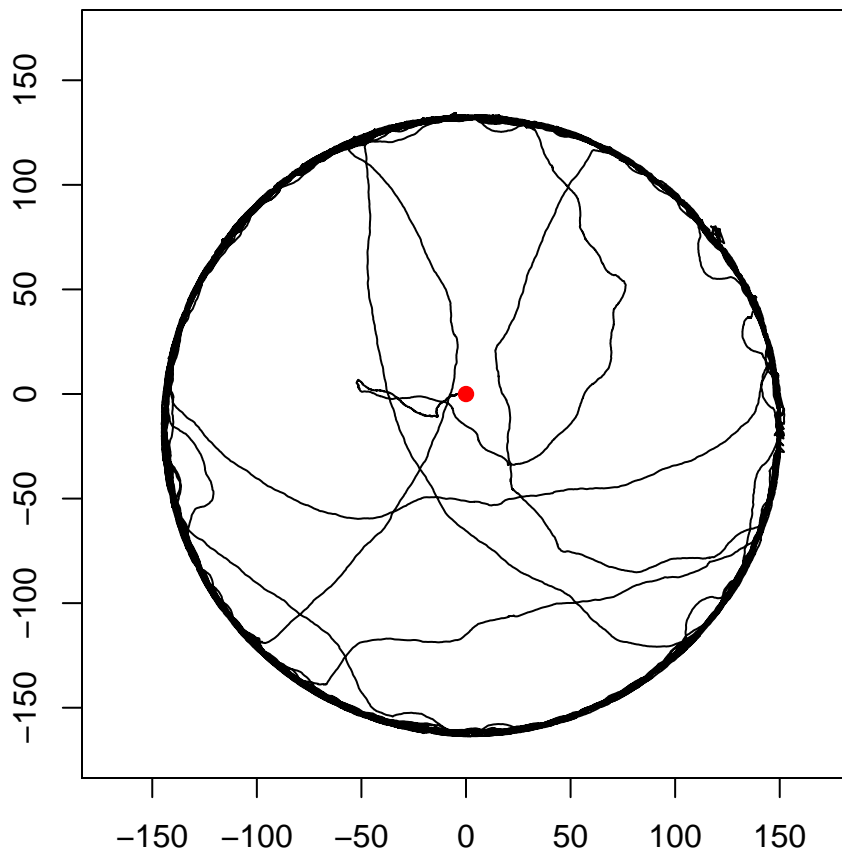

Supplement: S1 Datasets — (ZIP) [file pone.0177480.s006.zip › ANTAM data/trajectories/Small arena (5FPS)/d_08.pdf]

**d\_09**

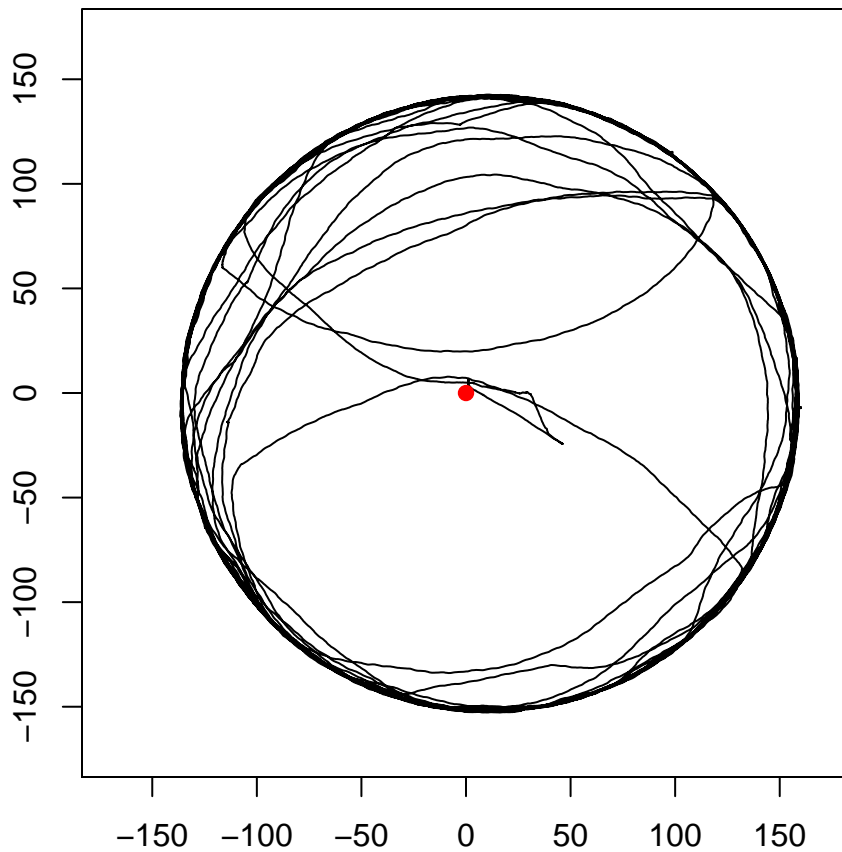

Supplement: S1 Datasets — (ZIP) [file pone.0177480.s006.zip › ANTAM data/trajectories/Small arena (5FPS)/d_09.pdf]

**d\_10**

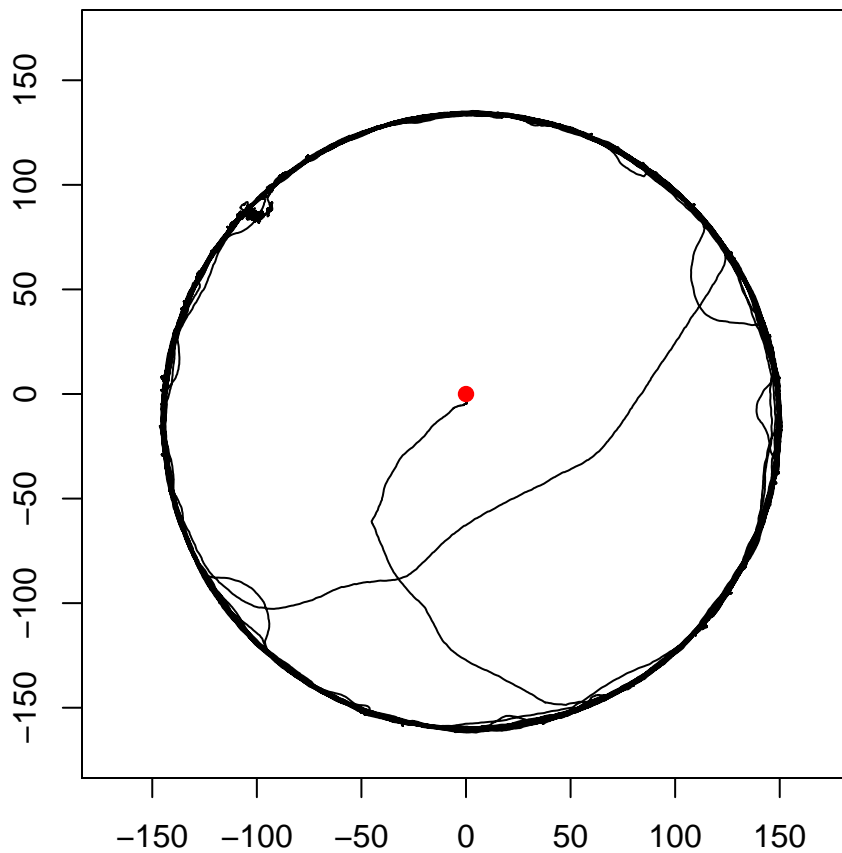

Supplement: S1 Datasets — (ZIP) [file pone.0177480.s006.zip › ANTAM data/trajectories/Small arena (5FPS)/d_10.pdf]

**d\_11**

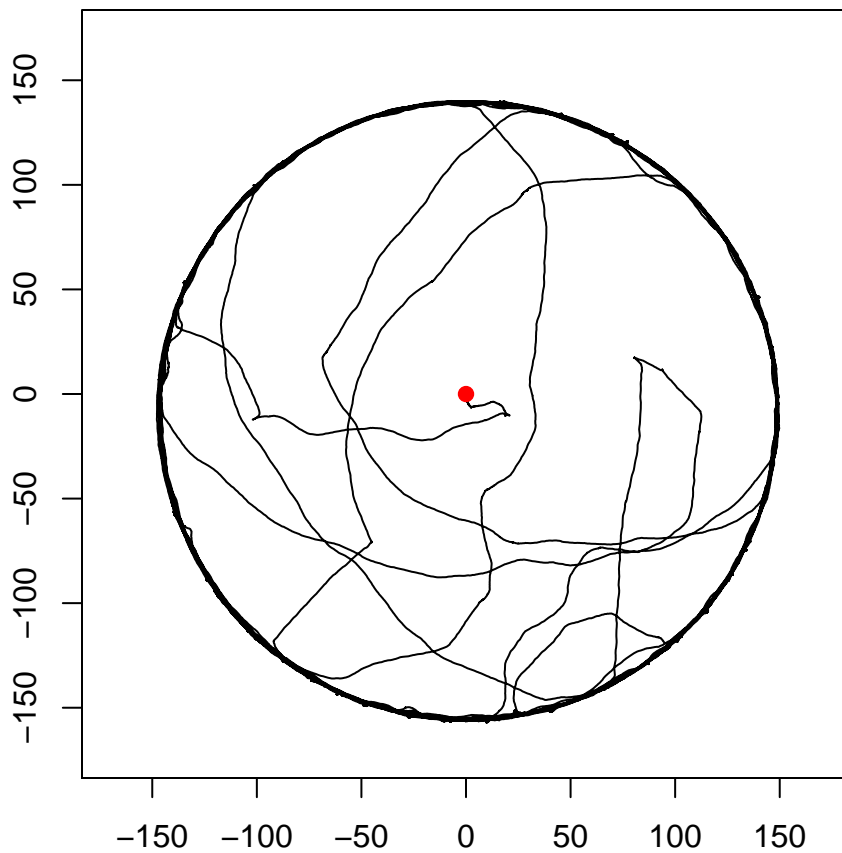

Supplement: S1 Datasets — (ZIP) [file pone.0177480.s006.zip › ANTAM data/trajectories/Small arena (5FPS)/d_11.pdf]

**d\_12**

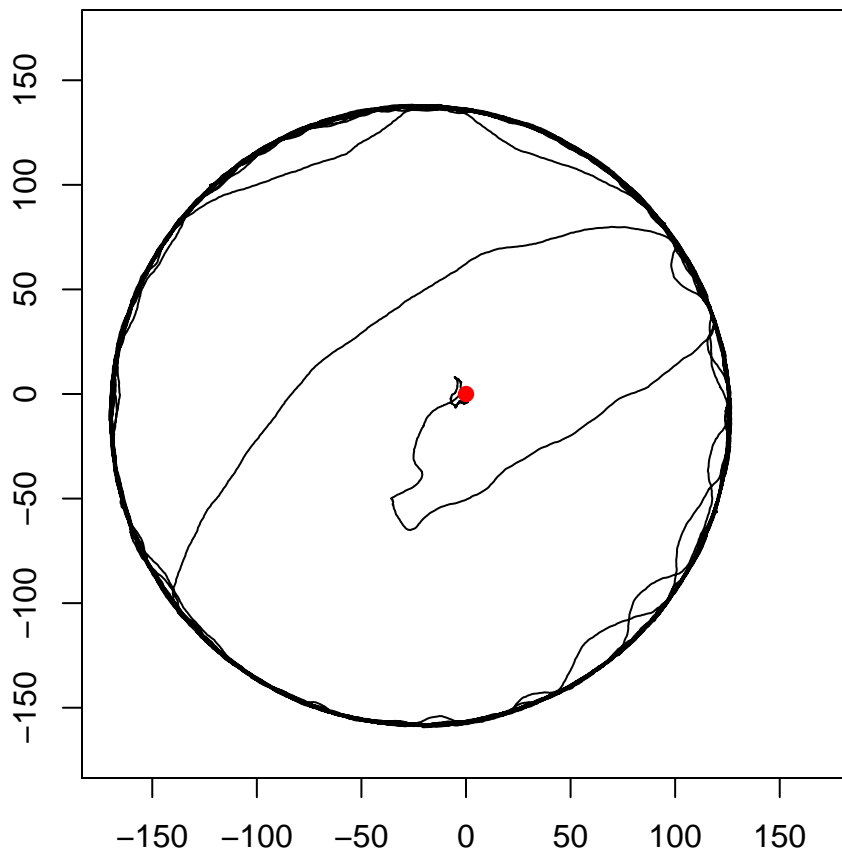

Supplement: S1 Datasets — (ZIP) [file pone.0177480.s006.zip › ANTAM data/trajectories/Small arena (5FPS)/d_12.pdf]

**d\_13**

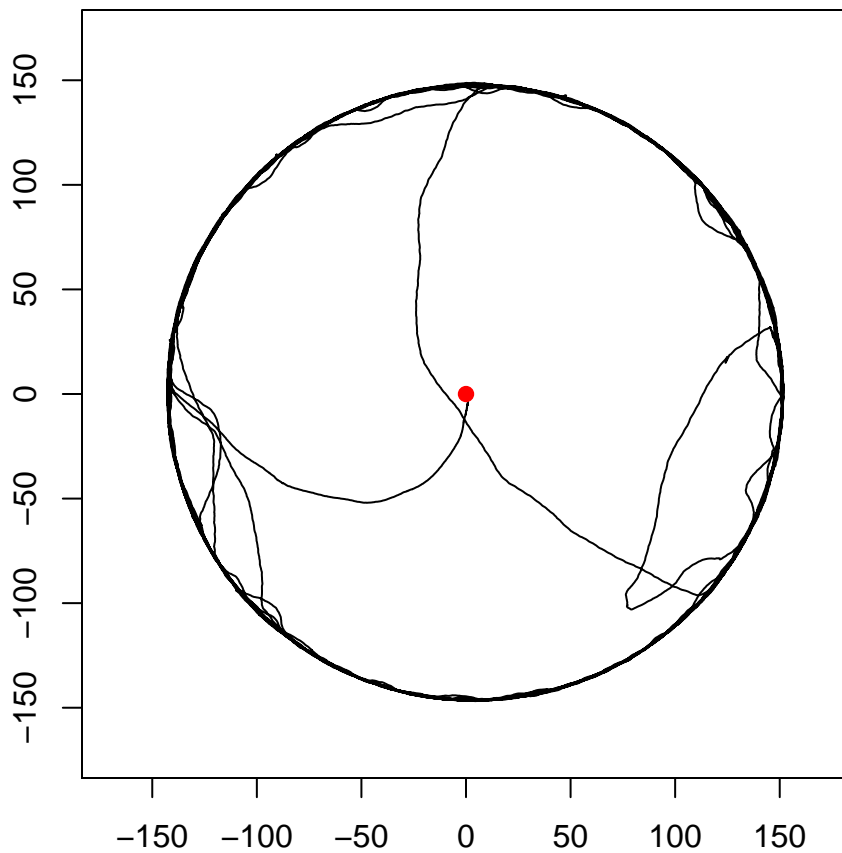

Supplement: S1 Datasets — (ZIP) [file pone.0177480.s006.zip › ANTAM data/trajectories/Small arena (5FPS)/d_13.pdf]

**d\_14**

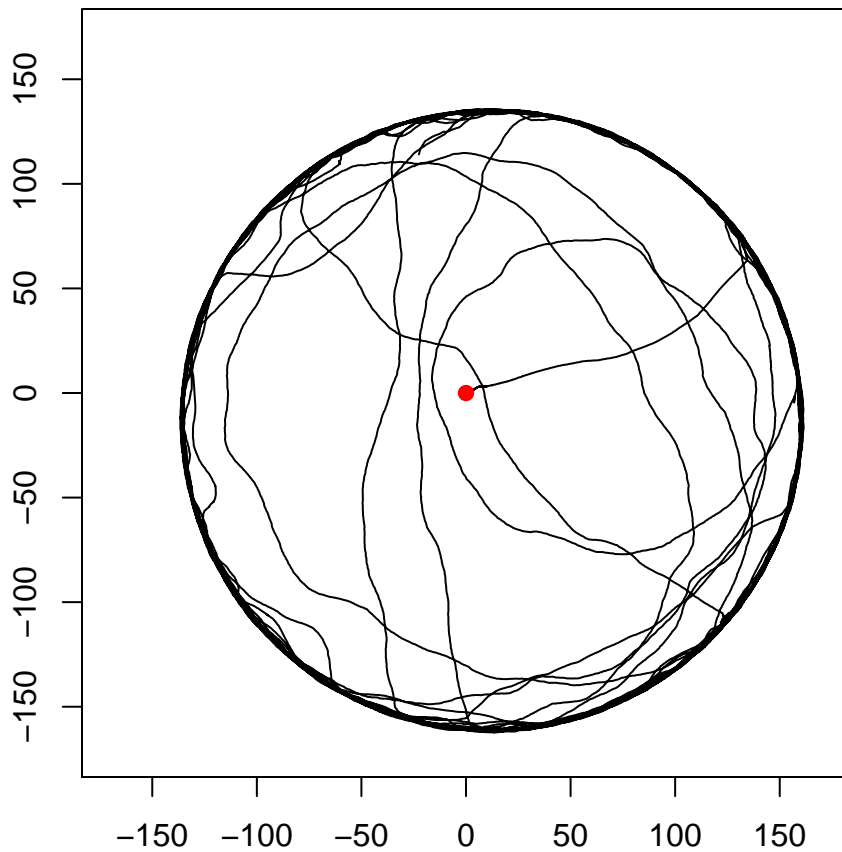

Supplement: S1 Datasets — (ZIP) [file pone.0177480.s006.zip › ANTAM data/trajectories/Small arena (5FPS)/d_14.pdf]

**d\_15**

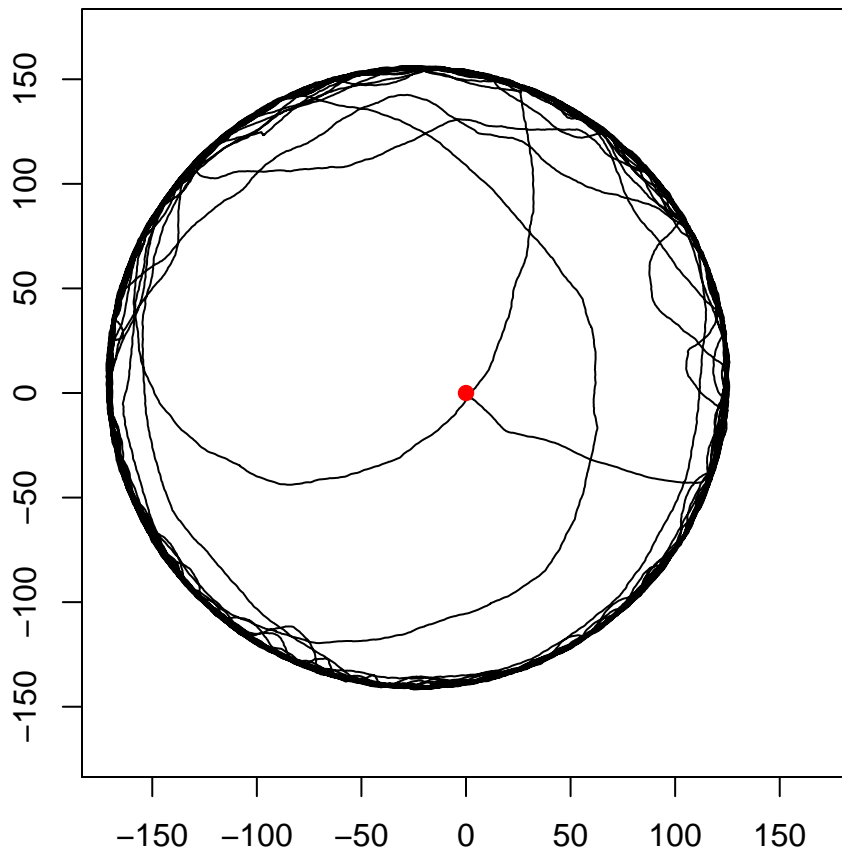

Supplement: S1 Datasets — (ZIP) [file pone.0177480.s006.zip › ANTAM data/trajectories/Small arena (5FPS)/d_15.pdf]

**d\_16**

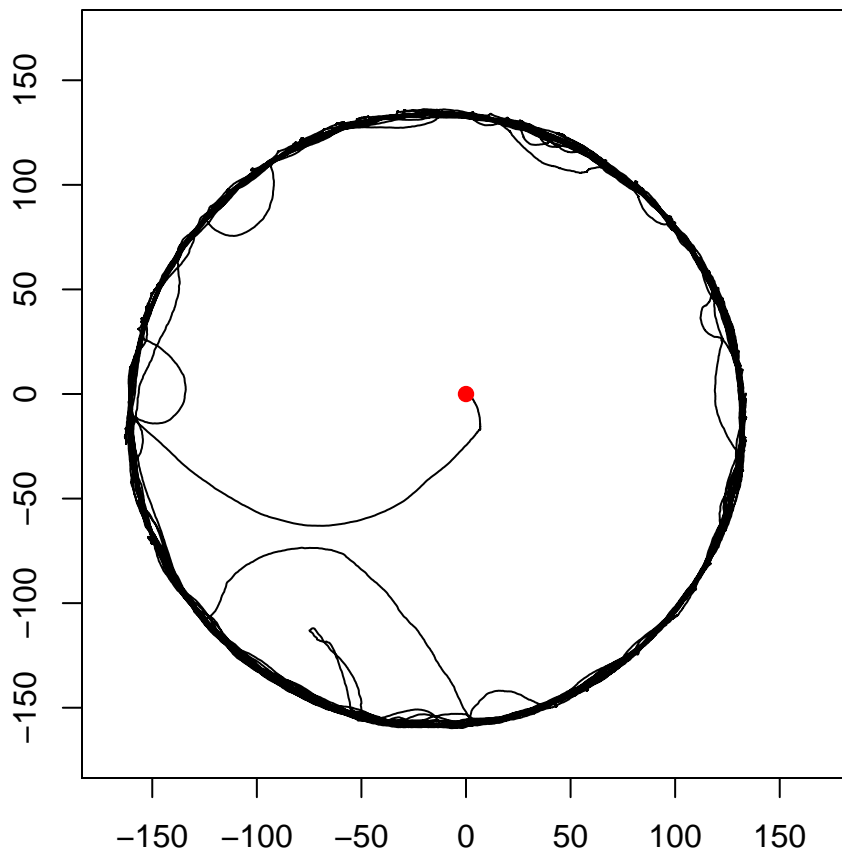

Supplement: S1 Datasets — (ZIP) [file pone.0177480.s006.zip › ANTAM data/trajectories/Small arena (5FPS)/d_16.pdf]

d\_17

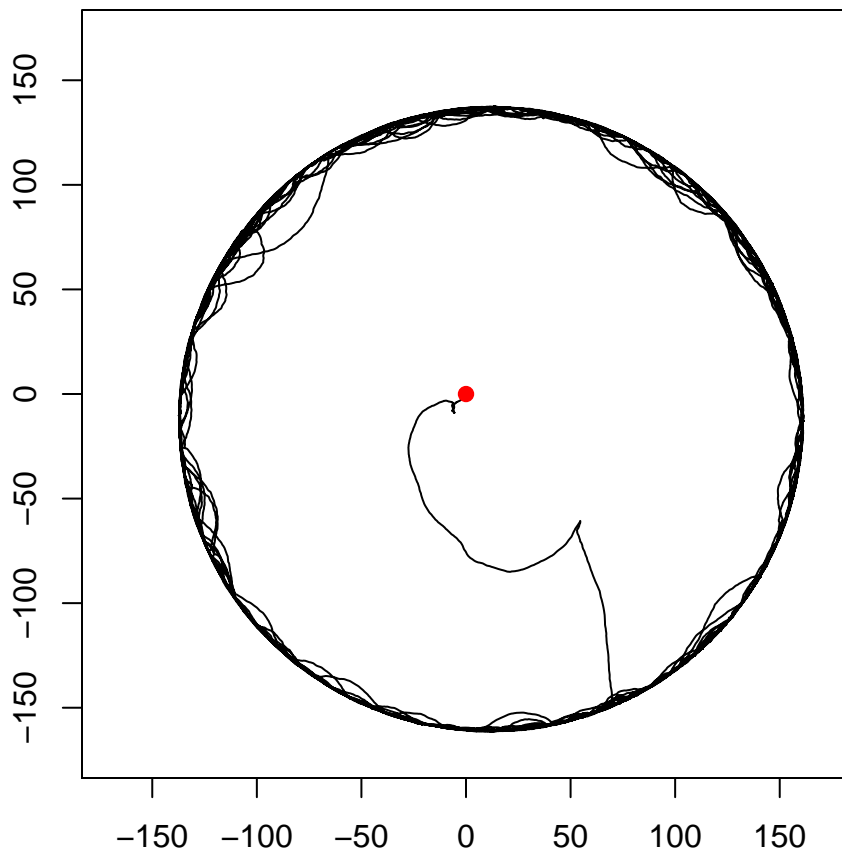

Supplement: S1 Datasets — (ZIP) [file pone.0177480.s006.zip › ANTAM data/trajectories/Small arena (5FPS)/d_17.pdf]

**d\_18**

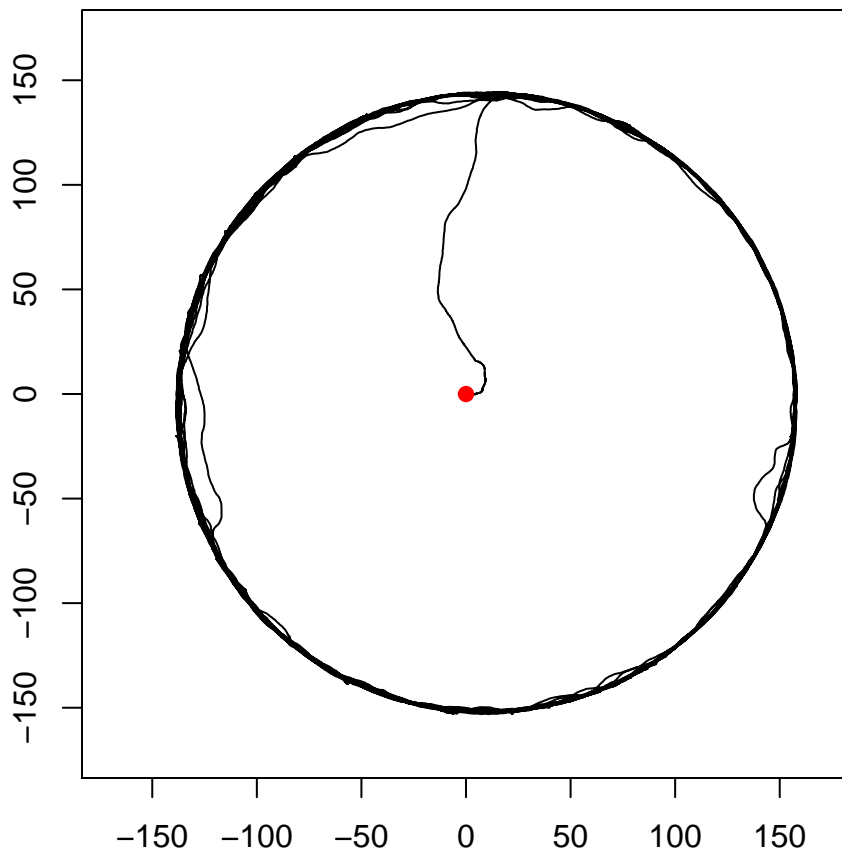

Supplement: S1 Datasets — (ZIP) [file pone.0177480.s006.zip › ANTAM data/trajectories/Small arena (5FPS)/d_18.pdf]

**d\_19**

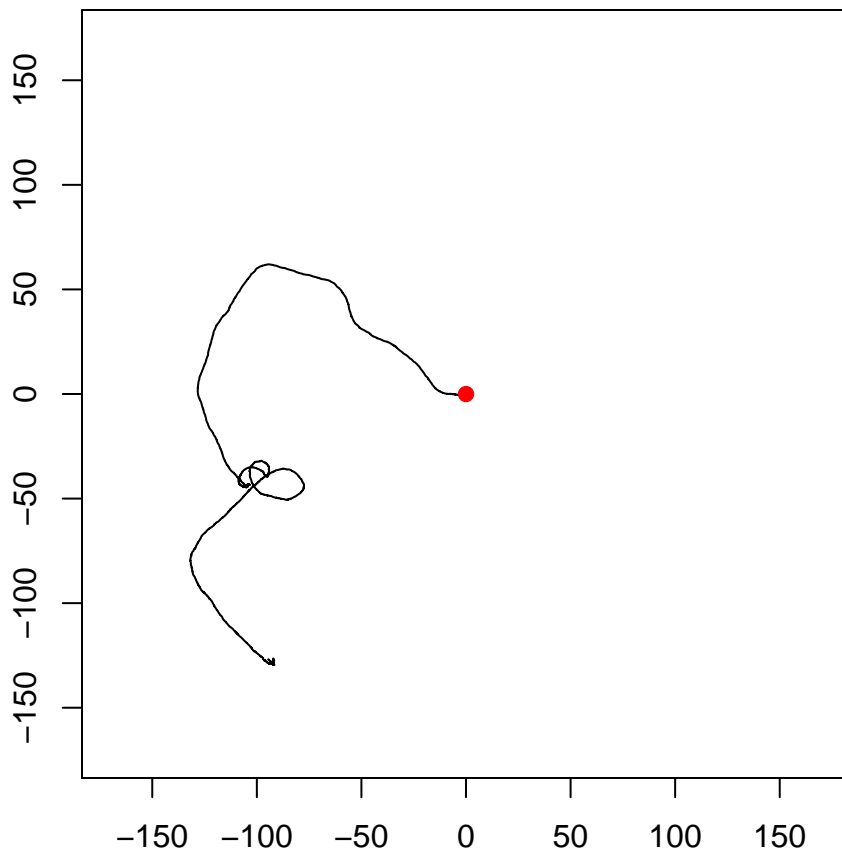

Supplement: S1 Datasets — (ZIP) [file pone.0177480.s006.zip › ANTAM data/trajectories/Small arena (5FPS)/d_19.pdf]

**d\_20**

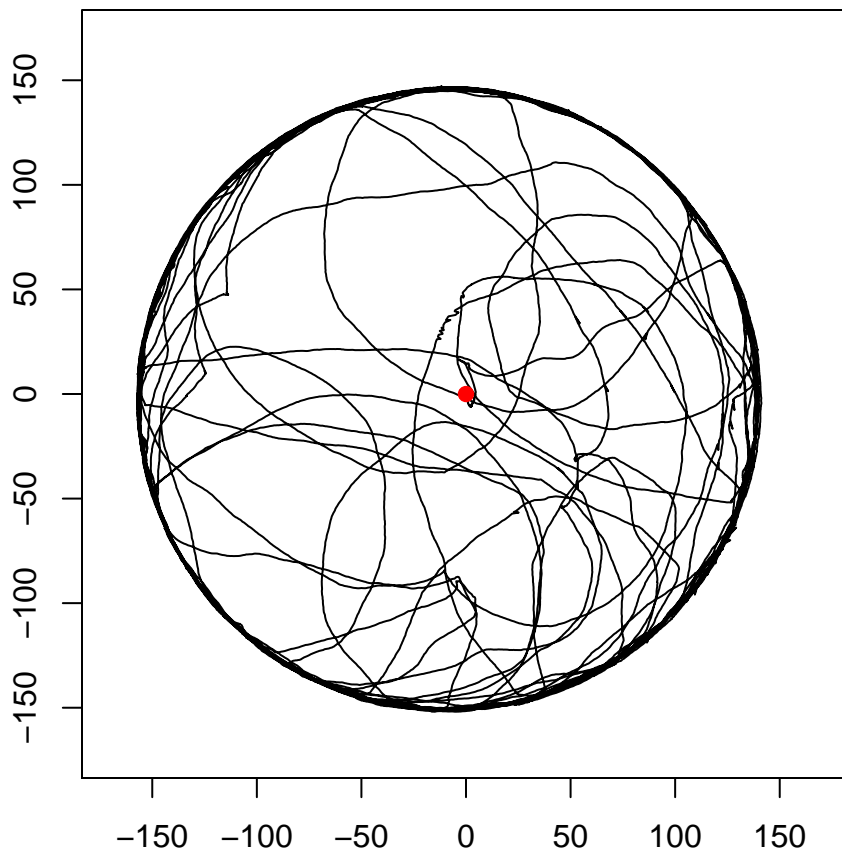

Supplement: S1 Datasets — (ZIP) [file pone.0177480.s006.zip › ANTAM data/trajectories/Small arena (5FPS)/d_20.pdf]

**d\_21**

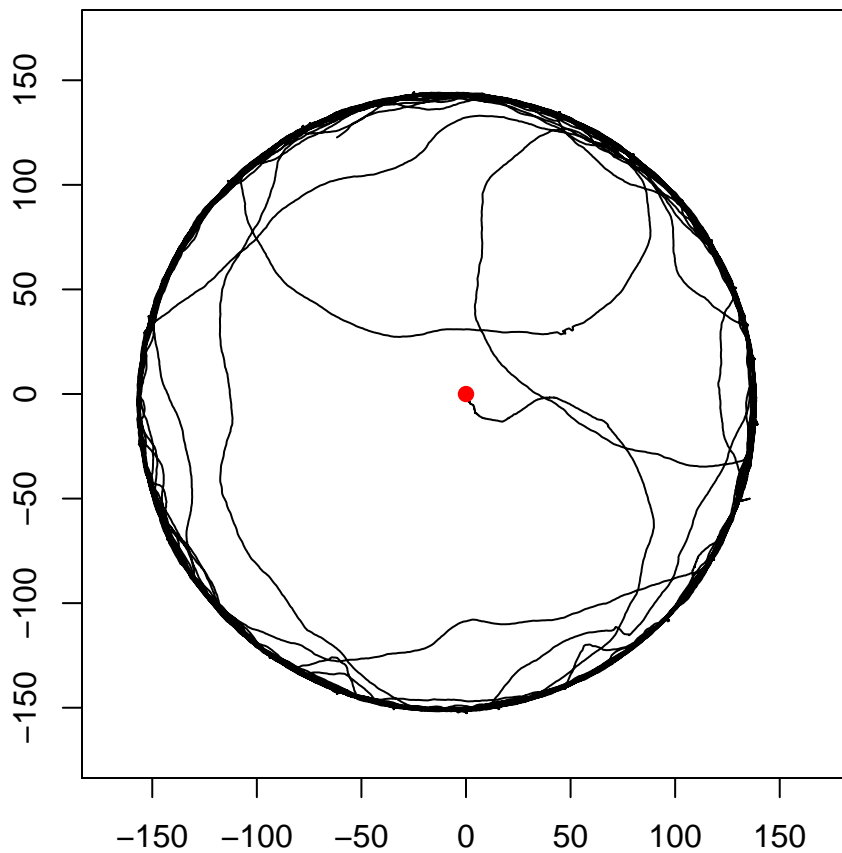

Supplement: S1 Datasets — (ZIP) [file pone.0177480.s006.zip › ANTAM data/trajectories/Small arena (5FPS)/d_21.pdf]

d\_22

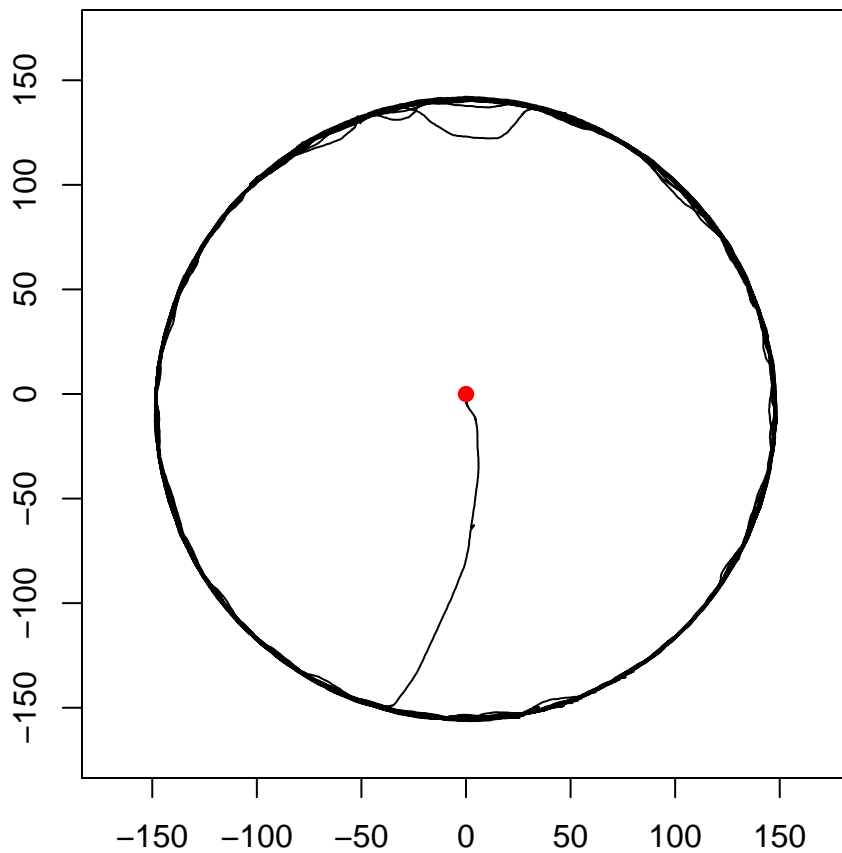

Supplement: S1 Datasets — (ZIP) [file pone.0177480.s006.zip › ANTAM data/trajectories/Small arena (5FPS)/d_22.pdf]

**d\_23**

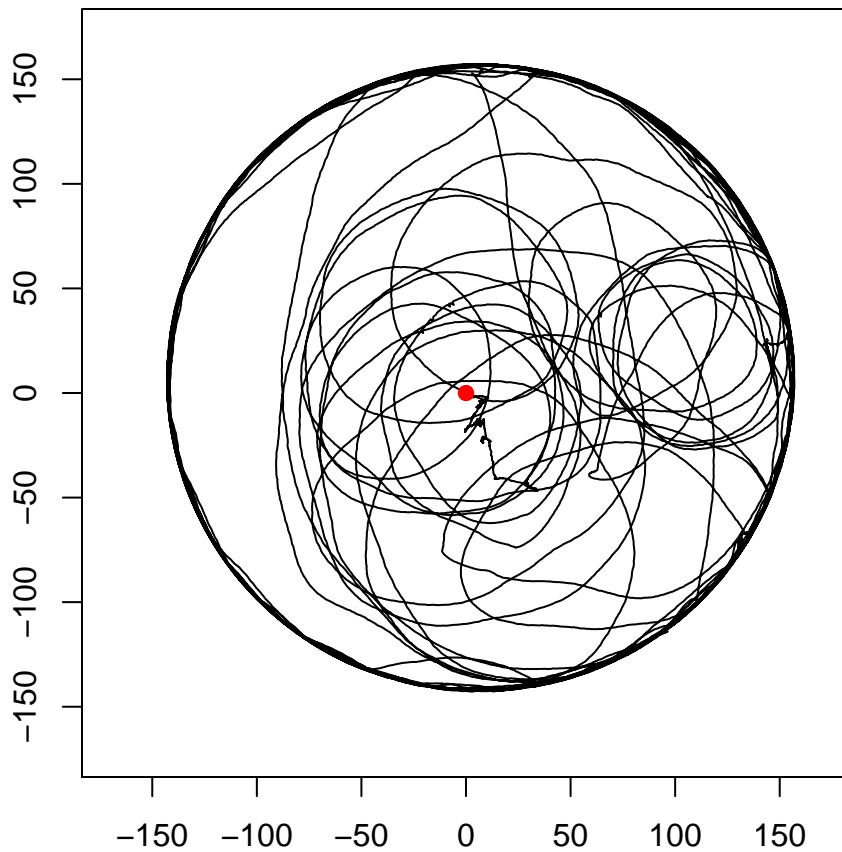

Supplement: S1 Datasets — (ZIP) [file pone.0177480.s006.zip › ANTAM data/trajectories/Small arena (5FPS)/d_23.pdf]

**d\_24**

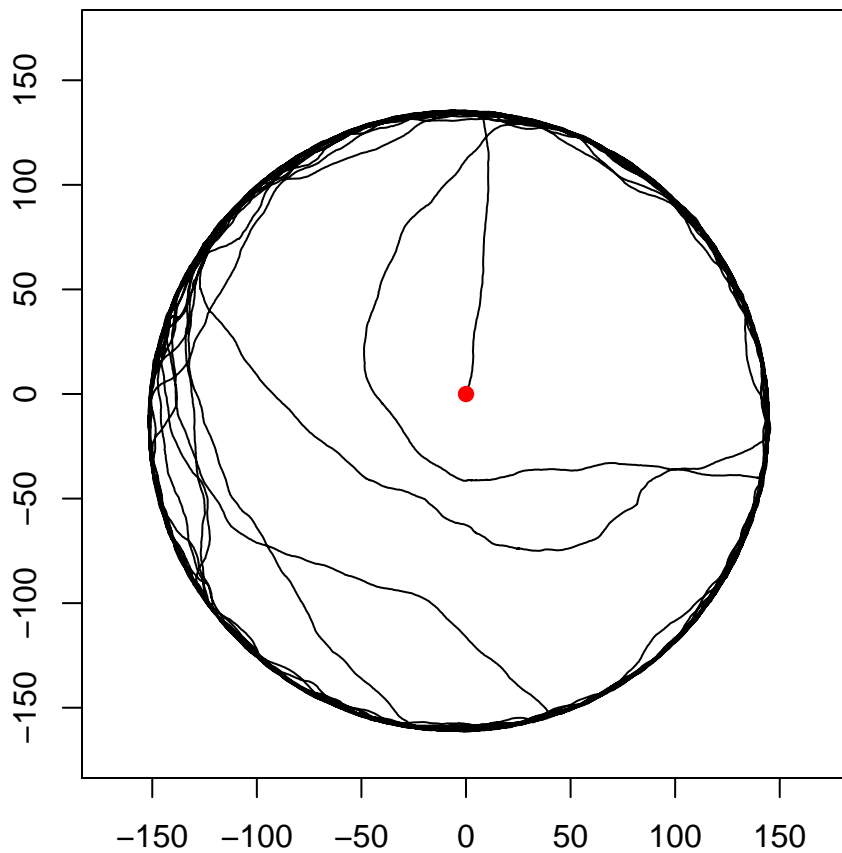

Supplement: S1 Datasets — (ZIP) [file pone.0177480.s006.zip › ANTAM data/trajectories/Small arena (5FPS)/d_24.pdf]

**d\_26**

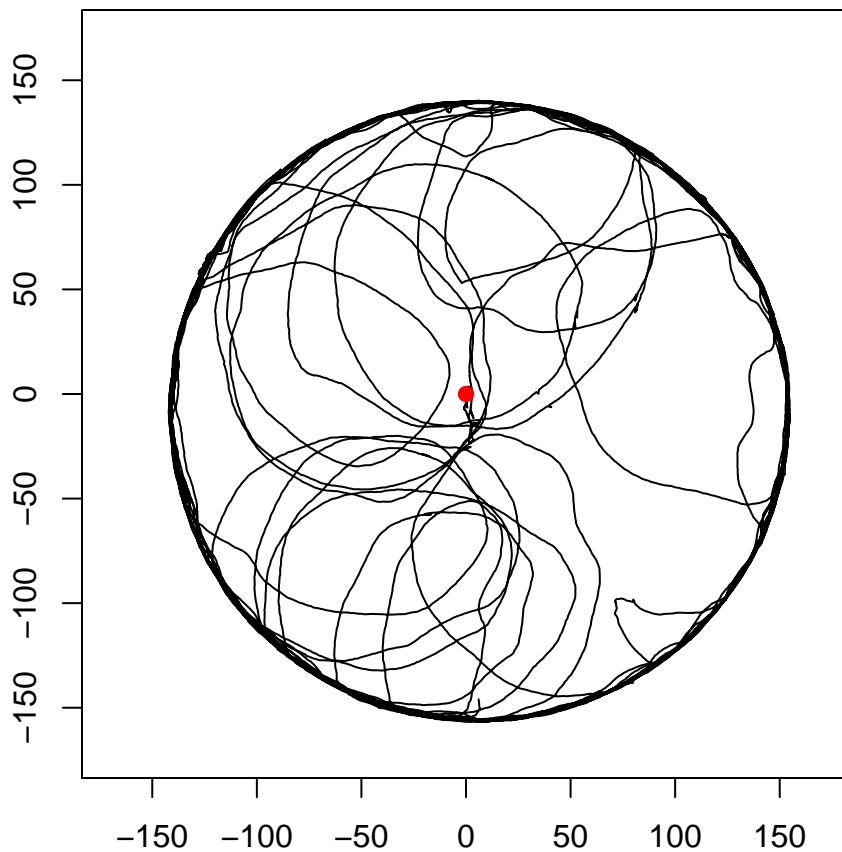

Supplement: S1 Datasets — (ZIP) [file pone.0177480.s006.zip › ANTAM data/trajectories/Small arena (5FPS)/d_26.pdf]

**d\_27**

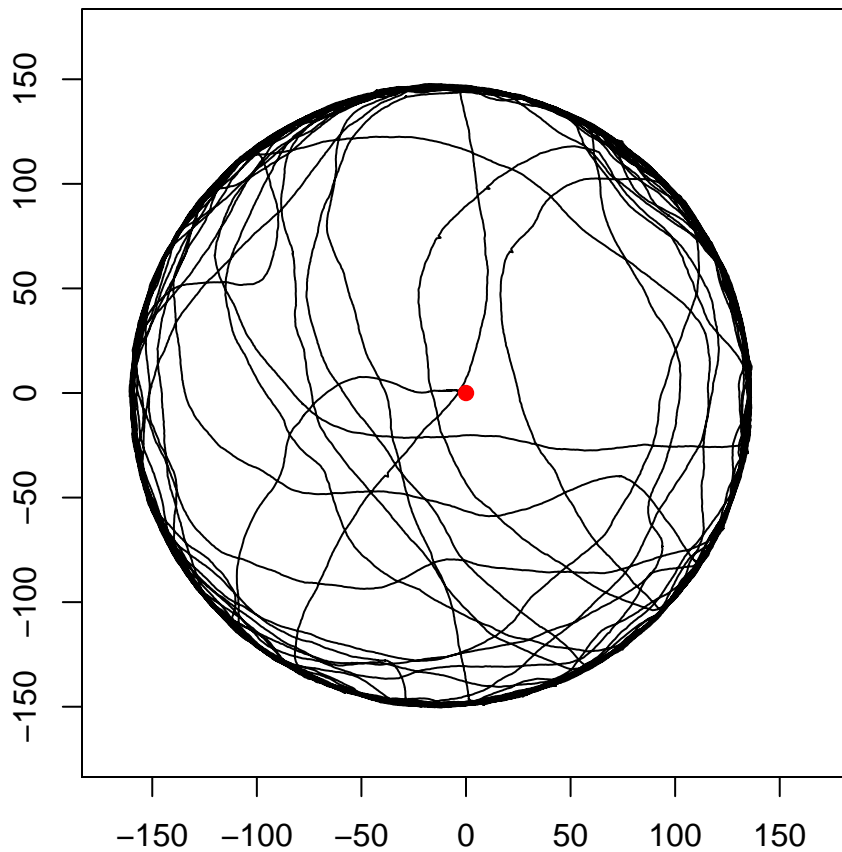

Supplement: S1 Datasets — (ZIP) [file pone.0177480.s006.zip › ANTAM data/trajectories/Small arena (5FPS)/d_27.pdf]

**d\_28**

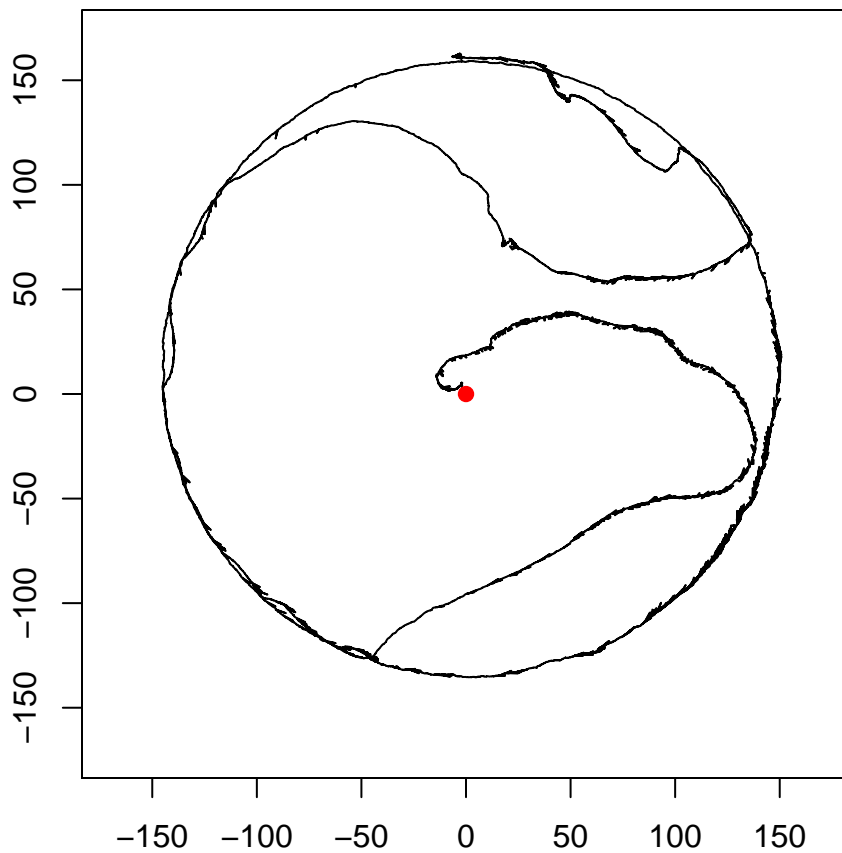

Supplement: S1 Datasets — (ZIP) [file pone.0177480.s006.zip › ANTAM data/trajectories/Small arena (5FPS)/d_28.pdf]

**d\_29**

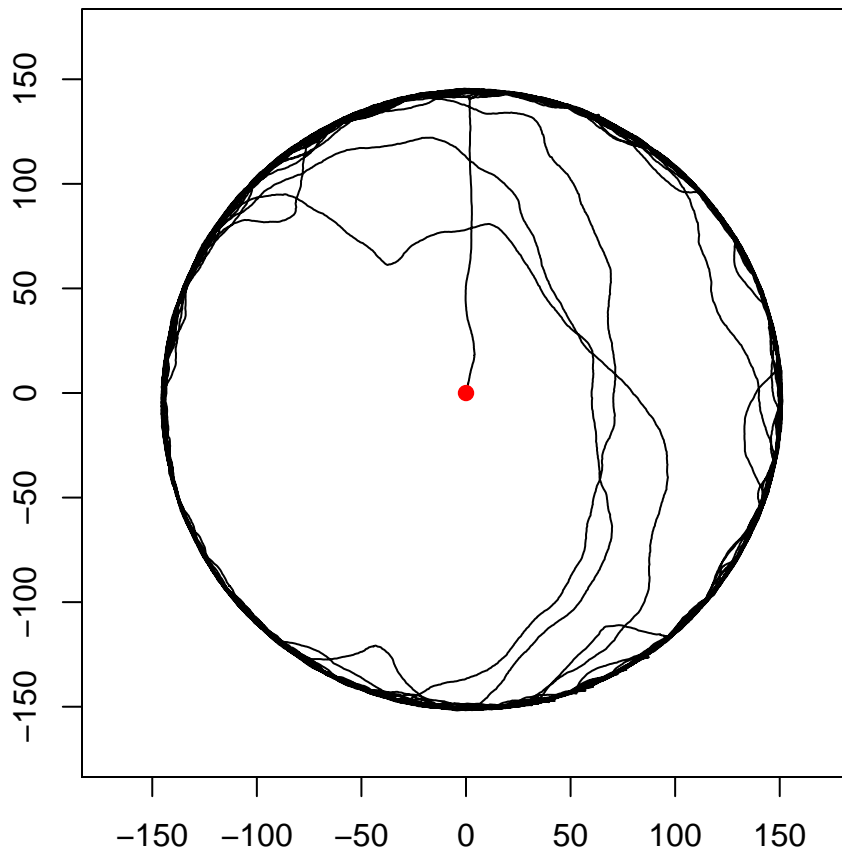

Supplement: S1 Datasets — (ZIP) [file pone.0177480.s006.zip › ANTAM data/trajectories/Small arena (5FPS)/d_29.pdf]
