# Supplementary material for: Anomalous diffusion on the servosphere: A potential tool for detecting inherent organismal movement patterns
Source: PLoS One. 2017 Jun 1;12(6):e0177480. doi: 10.1371/journal.pone.0177480 (PMC5453419; doi:10.1371/journal.pone.0177480)
Supplement: S2 Fig — Combinations of a letter and a number indicates the analyzed axis and the individuals. Black dots are observations, red and blue dashed lines are truncated Pareto and competing exponential distributions, respectively, fitted to the data. (PDF) [file pone.0177480.s002.pdf]

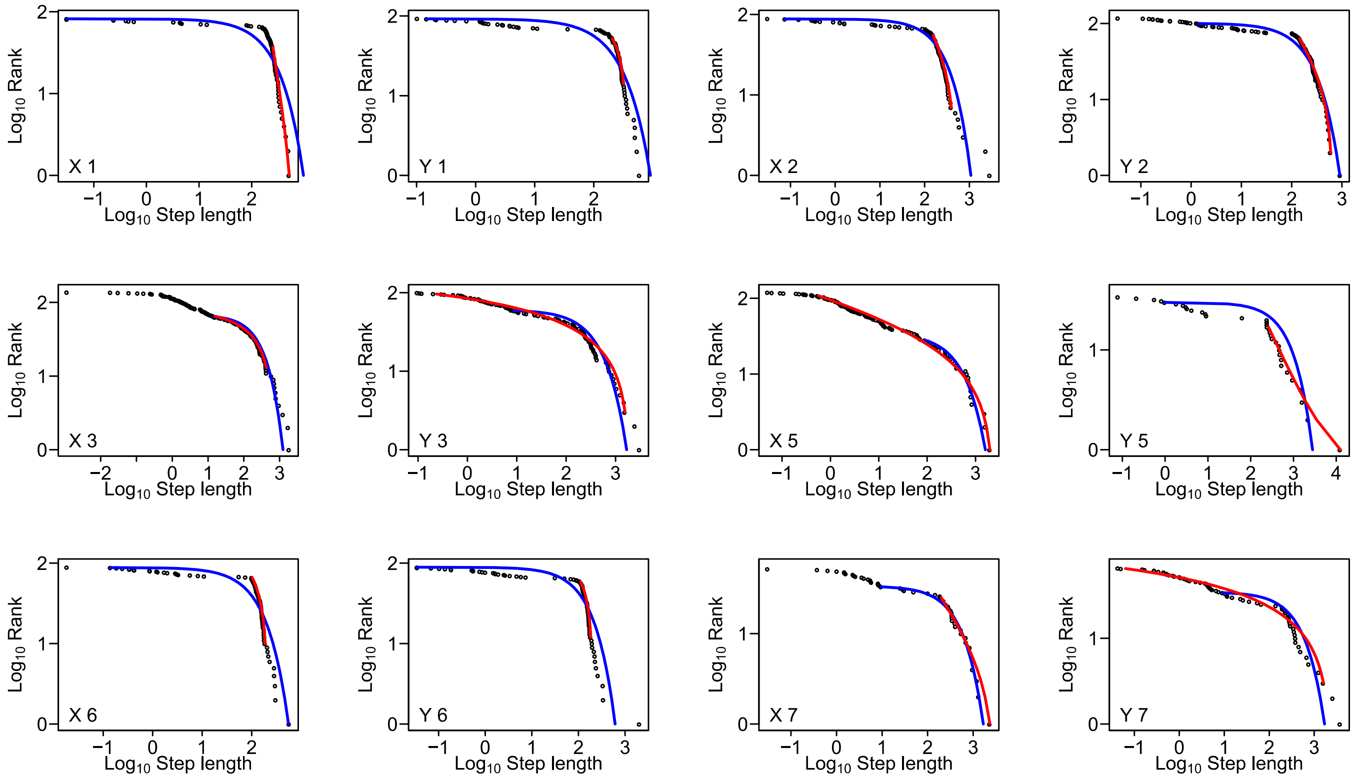

S2 Fig. The distribution of step lengths for all individuals.

Combinations of a letter and a number indicates the analyzed axis and the individuals. Black dots are observations, red and blue dashed lines are truncated Pareto and competing exponential distributions, respectively, fitted to the data.

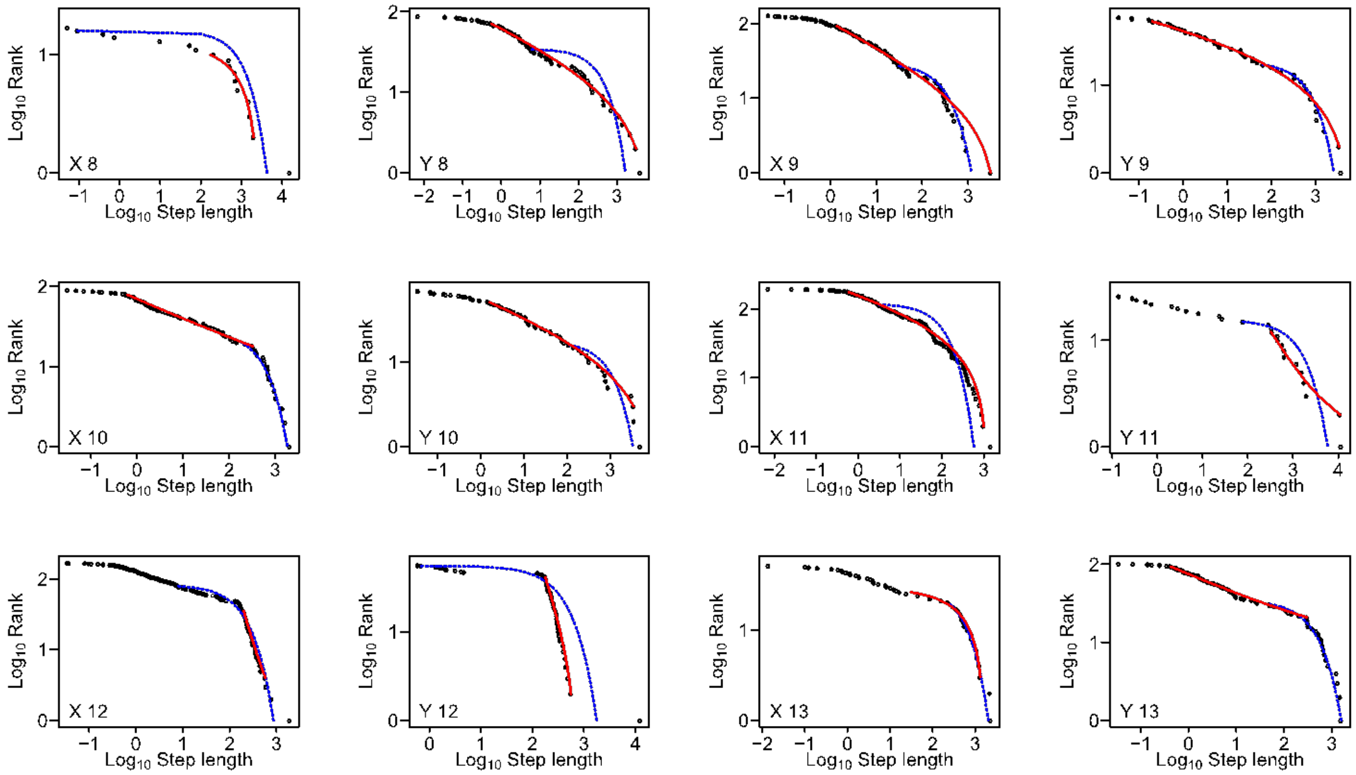

X 8
Y 8
X 9
Y 9
X 10
Y 10
X 11
Y 11
X 12
Y 12
X 13
Y 13
Log10 Rank
Log10 Step length

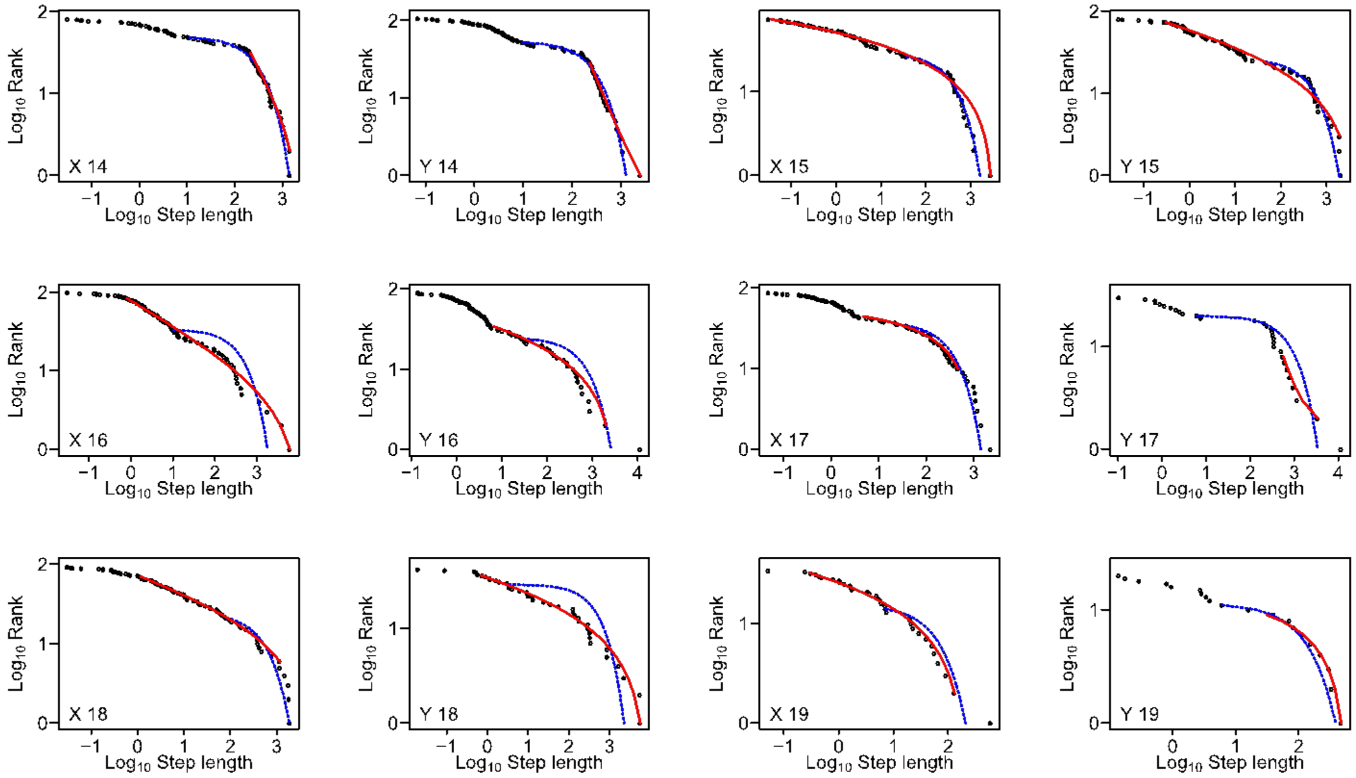

X 14
Y 14
X 15
Y 15
X 16
Y 16
X 17
Y 17
X 18
Y 18
X 19
Y 19
Log10 Rank
Log10 Step length

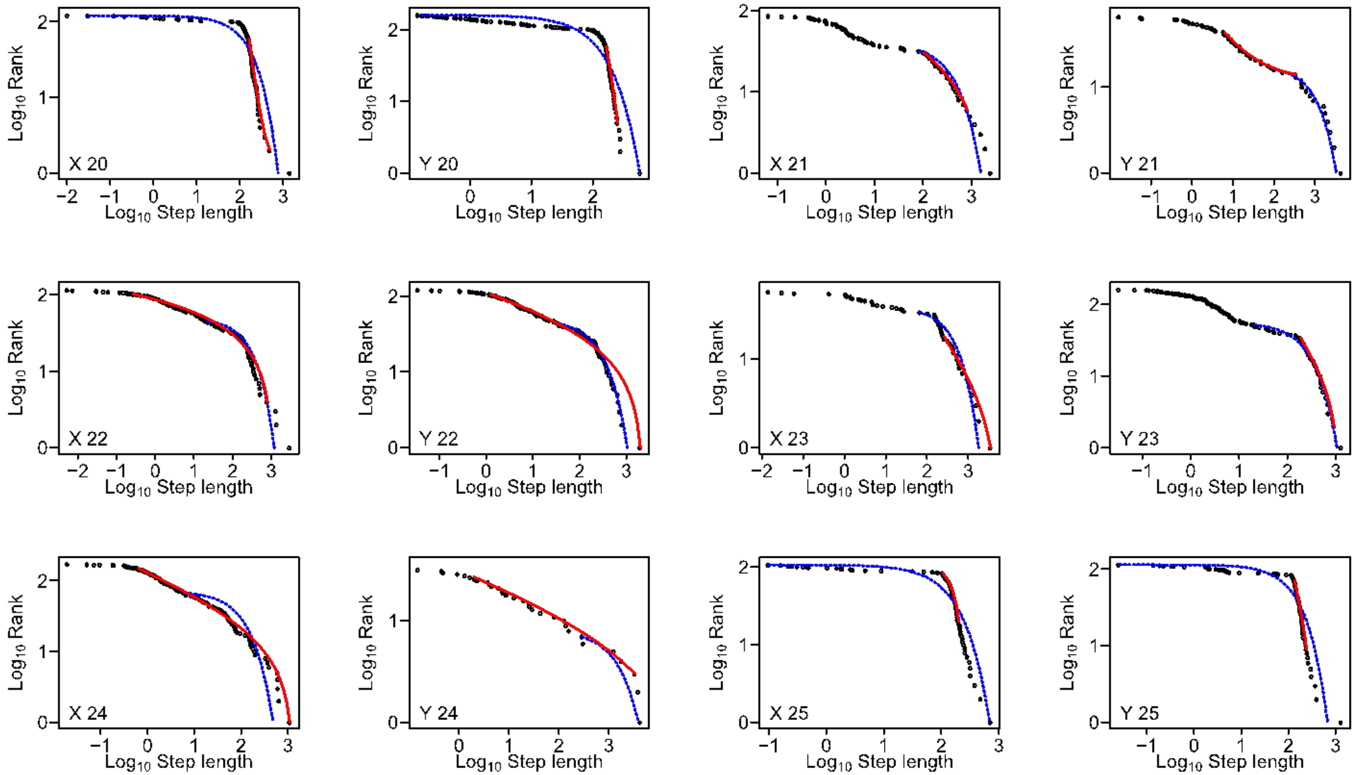

X 20
Y 20
X 21
Y 21
X 22
Y 22
X 23
Y 23
X 24
Y 24
X 25
Y 25
Log10 Rank
Log10 Step length

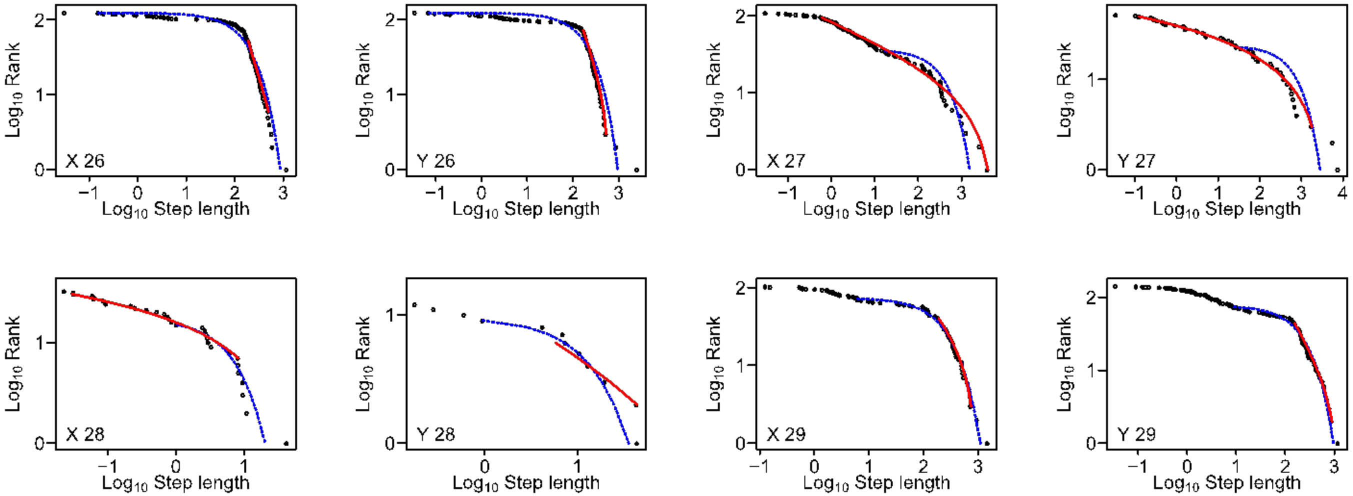
X 26
Y 26
X 27
Y 27
X 28
Y 28
X 29
Y 29
Log10 Rank
Log10 Step length
