# Supplementary material for: Anomalous diffusion on the servosphere: A potential tool for detecting inherent organismal movement patterns
Source: PLoS One. 2017 Jun 1;12(6):e0177480. doi: 10.1371/journal.pone.0177480 (PMC5453419; doi:10.1371/journal.pone.0177480)
Supplement: S1 Table — TP is truncated Pareto, Exp is exponential; OOM is order of magnitude. (PDF) [file pone.0177480.s003.pdf]

S1 Table. Maximum likelihood estimation analysis results for the individuals whose trajectories are determined as TP for both x and y axis.

TP is truncated Pareto, Exp is exponential; OOM is order of magnitude.

ID dimension	*Steps*	*Min step*	*Max step*	*TP min*	*TP max*	*TP Exponent*	*TP fitted steps*	*TP fit*	*Exp fit*	*Exp AICw*	*Exp CompAICw*	*TP AICw*	*TP CompAICw*	*TP OOM*	*Exp min*	*Exp exponent*	*Exp fitted steps*	*Exp OOM*	*judgement*
2 X	89	0.03	2895.37	153.52	394.66	1.77	50	0.05	0.14	1.00	0.00	0.00	1.00	0.41	0.08	4.E-03	88	4.58	TP
2 Y	118	0.04	927.80	141.71	596.46	1.87	64	0.08	0.16	0.00	1.00	1.00	0.00	0.62	1.29	5.E-03	101	2.86	TP
5 X	120	0.05	1992.76	0.55	1992.76	1.17	108	0.06	0.15	0.09	0.91	0.91	0.09	3.56	72.91	2.E-03	29	1.44	TP
5 Y	34	0.08	12472.56	245.28	12472.56	1.95	18	0.16	0.33	0.00	1.00	1.00	0.00	1.71	0.84	1.E-03	31	4.17	TP
9 X	128	0.04	3122.31	1.35	3122.31	1.31	94	0.05	0.35	0.00	1.00	1.00	0.00	3.37	31.70	3.E-03	27	1.99	TP
9 Y	59	0.04	3813.51	0.18	3422.39	1.09	54	0.06	0.21	0.06	0.94	0.94	0.06	4.29	64.48	1.E-03	17	1.77	TP
10 X	90	0.03	2091.14	0.58	323.07	1.26	63	0.07	0.16	0.13	0.87	0.87	0.13	2.75	86.40	2.E-03	24	1.38	TP
10 Y	70	0.04	4999.35	1.47	3487.10	1.19	50	0.06	0.41	0.00	1.00	1.00	0.00	3.37	110.65	9.E-04	16	1.65	TP
11 X	195	0.01	1473.58	0.60	995.74	1.16	175	0.05	0.31	0.00	1.00	1.00	0.00	3.22	3.13	8.E-03	115	2.67	TP
11 Y	26	0.14	11635.49	289.84	10926.40	1.74	13	0.17	0.42	0.00	1.00	1.00	0.00	1.58	27.36	5.E-04	16	2.63	TP
14 X	81	0.03	1439.71	209.77	1438.23	2.02	31	0.11	0.12	0.44	0.56	0.56	0.44	0.84	11.35	3.E-03	48	2.10	TP
14 Y	106	0.07	2471.69	225.09	2471.69	2.51	30	0.09	0.12	1.00	0.00	0.00	1.00	1.04	9.72	3.E-03	52	2.41	TP
15 X	73	0.03	2836.96	0.03	2836.96	1.01	73	0.06	0.12	0.95	0.05	0.05	0.95	4.96	31.17	2.E-03	27	1.96	TP
15 Y	80	0.03	2089.26	0.29	1941.83	1.13	72	0.08	0.17	0.01	0.99	0.99	0.01	3.83	24.49	2.E-03	25	1.93	TP
16 X	99	0.03	6128.97	0.78	6128.97	1.30	85	0.07	0.44	0.00	1.00	1.00	0.00	3.90	10.34	2.E-03	33	2.77	TP
16 Y	90	0.14	11463.86	5.87	2002.78	1.08	33	0.10	0.44	0.00	1.00	1.00	0.00	2.53	26.27	1.E-03	25	2.64	TP
18 X	94	0.03	1922.05	1.09	1160.55	1.19	66	0.05	0.25	0.00	1.00	1.00	0.00	3.03	77.14	2.E-03	22	1.40	TP
18 Y	42	0.02	5746.83	0.59	5746.83	1.07	37	0.10	0.43	0.00	1.00	1.00	0.00	3.99	3.35	1.E-03	29	3.23	TP
21 X	86	0.07	2495.66	100.28	812.97	1.29	26	0.09	0.16	0.85	0.15	0.15	0.85	0.91	42.14	2.E-03	33	1.77	TP
21 Y	66	0.02	4384.07	5.97	345.35	1.70	31	0.09	0.23	0.08	0.92	0.92	0.08	1.76	103.16	9.E-04	16	1.63	TP
23 X	58	0.01	3506.26	243.26	3506.26	1.65	19	0.13	0.20	1.00	0.00	0.00	1.00	1.16	28.47	2.E-03	35	2.09	TP
23 Y	161	0.03	1295.94	183.72	923.94	1.96	33	0.09	0.15	0.99	0.01	0.01	0.99	0.70	20.94	4.E-03	52	1.79	TP
24 X	171	0.02	1107.25	0.62	1107.25	1.28	150	0.04	0.30	0.00	1.00	1.00	0.00	3.25	5.98	9.E-03	68	2.27	TP
24 Y	32	0.15	4277.11	1.70	3360.35	1.18	25	0.07	0.49	0.00	1.00	1.00	0.00	3.30	161.96	6.E-04	8	1.42	TP
27 X	110	0.03	4032.82	0.59	4032.82	1.23	96	0.06	0.28	0.00	1.00	1.00	0.00	3.83	17.30	2.E-03	35	2.37	TP
27 Y	52	0.04	7646.19	0.11	1771.61	1.01	49	0.06	0.37	0.00	1.00	1.00	0.00	4.22	27.35	1.E-03	23	2.45	TP
29 X	103	0.13	1508.27	185.87	738.79	1.72	39	0.08	0.10	1.00	0.00	0.00	1.00	0.60	6.19	4.E-03	72	2.39	TP
29 Y	143	0.04	1207.30	154.53	897.75	2.13	44	0.10	0.13	1.00	0.00	0.00	1.00	0.76	8.69	5.E-03	75	2.14	TP
