# Supplementary material for: Anomalous diffusion on the servosphere: A potential tool for detecting inherent organismal movement patterns
Source: PLoS One. 2017 Jun 1;12(6):e0177480. doi: 10.1371/journal.pone.0177480 (PMC5453419; doi:10.1371/journal.pone.0177480)
Supplement: S2 Table — TP is truncated Pareto, Exp is exponential; OOM is order of magnitude. (PDF) [file pone.0177480.s004.pdf]

S2 Table. Maximum likelihood estimation analysis results for the individuals whose trajectories are determined as TP for either x or y axis.

TP is truncated Pareto, Exp is exponential; OOM is order of magnitude.

ID dimension	*Steps*	*Min step*	*Max step*	*TP min*	*TP max*	*TP Exponent*	*TP fitted steps*	*TP fit*	*Exp fit*	*Exp AICw*	*Exp CompAICw*	*TP AICw*	*TP CompAICw*	*TP OOM*	*Exp min*	*Exp exponent*	*Exp fitted steps*	*Exp OOM*	*judgement*
7 X	52	0.03	2336.57	189.59	2336.57	1.69	26	0.09	0.11	0.97	0.03	0.03	0.97	1.09	9.05	2.17E-03	34	2.41	TP
8 Y	88	0.01	4029.37	0.53	3125.92	1.22	71	0.08	0.43	0.00	1.00	1.00	0.00	3.77	5.66	2.21E-03	35	2.85	TP
12 Y	56	0.60	12445.61	176.13	560.67	2.75	41	0.08	0.33	0.00	1.00	1.00	0.00	0.50	0.60	2.31E-03	56	4.32	TP
13 Y	102	0.04	1571.35	0.42	298.91	1.28	75	0.08	0.14	0.00	1.00	1.00	0.00	2.86	50.25	2.24E-03	31	1.50	TP
17 Y	30	0.11	11583.43	513.93	3392.62	2.55	8	0.23	0.33	0.00	1.00	1.00	0.00	0.82	3.00	9.20E-04	21	3.59	TP
19 X	34	0.05	599.88	0.25	130.42	1.03	32	0.09	0.32	0.00	1.00	1.00	0.00	2.72	7.29	1.32E-02	14	1.92	TP
22 Y	120	0.04	1982.05	1.20	1982.05	1.16	106	0.06	0.09	1.00	0.00	0.00	1.00	3.22	36.16	3.70E-03	43	1.74	TP
26 Y	124	0.04	2553.07	167.37	529.55	2.63	70	0.07	0.16	1.00	0.00	0.00	1.00	0.50	0.07	4.98E-03	122	4.56	TP
28 Y	12	0.18	44.42	4.24	43.82	1.38	7	0.19	0.18	0.04	0.96	0.96	0.04	1.01	0.95	6.30E-02	9	1.67	TP
7 X	52	0.03	2336.57	189.59	2336.57	1.69	26	0.09	0.11	0.97	0.03	0.03	0.97	1.09	9.05	2.17E-03	34	2.41	TP
8 Y	88	0.01	4029.37	0.53	3125.92	1.22	71	0.08	0.43	0.00	1.00	1.00	0.00	3.77	5.66	2.21E-03	35	2.85	TP
12 Y	56	0.60	12445.61	176.13	560.67	2.75	41	0.08	0.33	0.00	1.00	1.00	0.00	0.50	0.60	2.31E-03	56	4.32	TP
13 Y	102	0.04	1571.35	0.42	298.91	1.28	75	0.08	0.14	0.00	1.00	1.00	0.00	2.86	50.25	2.24E-03	31	1.50	TP
17 Y	30	0.11	11583.43	513.93	3392.62	2.55	8	0.23	0.33	0.00	1.00	1.00	0.00	0.82	3.00	9.20E-04	21	3.59	TP
19 X	34	0.05	599.88	0.25	130.42	1.03	32	0.09	0.32	0.00	1.00	1.00	0.00	2.72	7.29	1.32E-02	14	1.92	TP
