# Supplementary material for: Anomalous diffusion on the servosphere: A potential tool for detecting inherent organismal movement patterns
Source: PLoS One. 2017 Jun 1;12(6):e0177480. doi: 10.1371/journal.pone.0177480 (PMC5453419; doi:10.1371/journal.pone.0177480)
Supplement: S3 Table — TP is truncated Pareto, Exp is exponential; OOM is order of magnitude. (PDF) [file pone.0177480.s005.pdf]

S3 Table. Maximum likelihood estimation analysis results for the individuals whose trajectories are determined as unclassified.

TP is truncated Pareto, Exp is exponential; OOM is order of magnitude.

ID dimension	*Steps*	*Min step*	*Max step*	*TP min*	*TP max*	*TP Exponent*	*TP fitted steps*	*TP fit*	*Exp fit*	*Exp AICw*	*Exp CompAICw*	*TP AICw*	*TP CompAICw*	*TP OOM*	*Exp min*	*Exp exponent*	*Exp fitted steps*	*Exp OOM*	*judgement*
1 X	82	0.03	518.69	251.09	518.69	5.11	38	0.09	0.32	0.00	1.00	1.00	0.00	0.32	0.03	4.57E-03	82	4.22	unclassified
1 Y	93	0.10	597.40	202.60	320.39	0.01	41	0.10	0.24	0.00	1.00	1.00	0.00	0.20	0.10	5.02E-03	93	3.76	unclassified
3 X	138	0.00	1866.99	13.83	422.60	0.61	54	0.07	0.11	0.95	0.05	0.05	0.95	1.49	14.01	3.43E-03	65	2.12	unclassified
3 Y	100	0.10	3078.83	0.21	1557.39	0.93	95	0.07	0.16	0.00	1.00	1.00	0.00	3.88	7.81	2.47E-03	60	2.60	unclassified
6 X	89	0.02	575.72	101.67	187.89	0.64	57	0.07	0.30	0.00	1.00	1.00	0.00	0.27	0.02	8.14E-03	89	4.49	unclassified
6 Y	89	0.04	2049.76	109.28	183.53	0.01	48	0.09	0.21	0.34	0.66	0.66	0.34	0.23	0.04	7.33E-03	89	4.76	unclassified
7 Y	66	0.04	3811.55	0.05	1614.17	0.95	63	0.09	0.25	0.00	1.00	1.00	0.00	4.50	7.63	2.14E-03	35	2.70	unclassified
8 X	17	0.05	15573.51	78.57	2007.89	0.45	10	0.20	0.34	0.00	1.00	1.00	0.00	1.41	0.09	6.56E-04	16	5.22	unclassified
12 X	169	0.03	1935.36	198.18	582.76	3.30	33	0.11	0.16	1.00	0.00	0.00	1.00	0.47	8.11	5.13E-03	79	2.38	unclassified
13 X	51	0.01	2325.84	16.84	1322.19	0.47	24	0.10	0.10	0.10	0.90	0.90	0.10	1.90	230.70	1.65E-03	20	1.00	unclassified
17 X	87	0.05	2276.66	3.75	461.76	0.76	35	0.08	0.20	0.01	0.99	0.99	0.01	2.09	25.35	2.55E-03	35	1.95	unclassified
19 Y	20	0.14	475.01	16.21	475.01	0.64	10	0.13	0.15	0.03	0.97	0.97	0.03	1.47	6.14	6.26E-03	11	1.89	unclassified
20 X	121	0.01	1429.74	161.86	489.89	5.04	59	0.09	0.27	1.00	0.00	0.00	1.00	0.48	0.01	6.32E-03	121	5.14	unclassified
20 Y	160	0.14	595.78	166.05	251.93	5.89	55	0.07	0.23	0.00	1.00	1.00	0.00	0.18	0.14	8.78E-03	160	3.62	unclassified
22 X	115	0.01	3009.23	0.25	790.05	0.99	100	0.06	0.16	0.29	0.71	0.71	0.29	3.50	22.04	3.27E-03	45	2.14	unclassified
25 X	107	0.10	726.77	105.95	200.88	0.11	65	0.06	0.29	1.00	0.00	0.00	1.00	0.28	0.10	6.72E-03	107	3.87	unclassified
25 Y	114	0.03	1316.11	134.61	242.05	3.72	65	0.07	0.30	1.00	0.00	0.00	1.00	0.25	0.03	7.01E-03	114	4.71	unclassified
26 X	125	0.03	1192.07	188.60	497.36	3.14	48	0.07	0.15	1.00	0.00	0.00	1.00	0.42	0.03	5.55E-03	125	4.58	unclassified
28 X	32	0.02	43.00	0.02	8.26	0.99	26	0.12	0.24	0.59	0.41	0.41	0.59	2.56	1.03	1.38E-01	15	1.62	unclassified
1 X	82	0.03	518.69	251.09	518.69	5.11	38	0.09	0.32	0.00	1.00	1.00	0.00	0.32	0.03	4.57E-03	82	4.22	unclassified
1 Y	93	0.10	597.40	202.60	320.39	0.01	41	0.10	0.24	0.00	1.00	1.00	0.00	0.20	0.10	5.02E-03	93	3.76	unclassified
3 X	138	0.00	1866.99	13.83	422.60	0.61	54	0.07	0.11	0.95	0.05	0.05	0.95	1.49	14.01	3.43E-03	65	2.12	unclassified
3 Y	100	0.10	3078.83	0.21	1557.39	0.93	95	0.07	0.16	0.00	1.00	1.00	0.00	3.88	7.81	2.47E-03	60	2.60	unclassified

6 X	89	0.02	575.72	101.67	187.89	0.64	57	0.07	0.30	0.00	1.00	1.00	0.00	0.27	0.02	8.14E-03	89	4.49	unclassified
6 Y	89	0.04	2049.76	109.28	183.53	0.01	48	0.09	0.21	0.34	0.66	0.66	0.34	0.23	0.04	7.33E-03	89	4.76	unclassified
7 Y	66	0.04	3811.55	0.05	1614.17	0.95	63	0.09	0.25	0.00	1.00	1.00	0.00	4.50	7.63	2.14E-03	35	2.70	unclassified
8 X	17	0.05	15573.51	78.57	2007.89	0.45	10	0.20	0.34	0.00	1.00	1.00	0.00	1.41	0.09	6.56E-04	16	5.22	unclassified
12 X	169	0.03	1935.36	198.18	582.76	3.30	33	0.11	0.16	1.00	0.00	0.00	1.00	0.47	8.11	5.13E-03	79	2.38	unclassified
